# A practical problem with Egger regression in Mendelian randomization

**DOI:** 10.1371/journal.pgen.1010166

**Published:** 2022-05-04

**Authors:** Zhaotong Lin, Isaac Pan, Wei Pan

**Affiliations:** 1 Division of Biostatistics, University of Minnesota, Minneapolis, Minnesota, United States of America; 2 Pomona College, Claremont, California, United States of America; University Hospital of the Canton Vaud (CHUV), SWITZERLAND

## Abstract

Mendelian randomization (MR) is an instrumental variable (IV) method using genetic variants such as single nucleotide polymorphisms (SNPs) as IVs to disentangle the causal relationship between an exposure and an outcome. Since any causal conclusion critically depends on the three valid IV assumptions, which will likely be violated in practice, MR methods robust to the IV assumptions are greatly needed. As such a method, Egger regression stands out as one of the most widely used due to its easy use and perceived robustness. Although Egger regression is claimed to be robust to directional pleiotropy under the instrument strength independent of direct effect (InSIDE) assumption, it is known to be dependent on the orientations/coding schemes of SNPs (i.e. which allele of an SNP is selected as the reference group). The current practice, as recommended as the default setting in some popular MR software packages, is to orientate the SNPs to be all positively associated with the exposure, which however, to our knowledge, has not been fully studied to assess its robustness and potential impact. We use both numerical examples (with both real data and simulated data) and analytical results to demonstrate the practical problem of Egger regression with respect to its heavy dependence on the SNP orientations. Under the assumption that InSIDE holds for some specific (and *unknown*) coding scheme of the SNPs, we analytically show that other coding schemes would in general lead to the violation of InSIDE. Other related MR and IV regression methods may suffer from the same problem. Cautions should be taken when applying Egger regression (and related MR and IV regression methods) in practice.

## Author summary

Egger regression (MR-Egger) has been increasingly applied in Mendelian randomization (MR) analyses for its easy use and perceived robustness, though MR-Egger requires the InSIDE assumption, which in turn depends on the orientation of SNPs. The implications of this dependence to its practical use may not be well understood yet. In particular, it is unrealistic to assume that the InSIDE assumption holds for many or all arbitrarily chosen coding schemes of the SNPs; instead, it is more reasonable to assume that InSIDE holds for only one specific, but usually unknown, coding scheme of SNPs, under which, however, we show that use of other coding schemes of SNPs in general leads to the violation of InSIDE, and thus to biased causal estimates. The technical reason is due to the seemingly non-restrictive assumption of the random direct effects with a non-zero mean (i.e. directional pleiotropy) in MR-Egger (and related methods), which depends on the orientation of SNPs. This problem persists for many other related MR and instrumental variable regression methods, regardless whether GWAS summary data or individual-level data are used. Given the popularity of MR-Egger in practice, one should be aware of this issue and hence be cautious when applying MR-Egger.

## Introduction

With the increasing availability of large-scale GWAS summary data nowadays, Mendelian randomization (MR) has become a useful tool in epidemiologic studies for identifying determinants or causes of a complex trait or disease [1–3]. In particular, the validity of MR findings relies on three important instrumental variable (IV) assumptions, in which a valid IV used in MR must be

(i) associated with the exposure *X* (relevance assumption);
(ii) not associated with any hidden confounder *U* (independence assumption);
(iii) not associated with the outcome *Y* conditional on the exposure and hidden confounder (exclusion restriction).

While assumption (i) is more likely to hold by selecting IVs strongly associated with the exposure, violations of assumptions (ii) and/or (iii) are more common in practice due to the wide-spread horizontal pleiotropy. In particular, violation of assumption (ii) introduces the so-called correlated pleiotropy (i.e. the pleiotropic effects of SNPs on *Y* are correlated with their effects on *X*); uncorrelated pleiotropy results if assumption (iii) is violated and the direct effects on *Y* are uncorrelated with those on *X*. Egger regression is an MR method that could give a consistent estimate when the exclusion restriction assumption is violated for *all* IVs, but requiring a milder so-called InSIDE assumption, that is, Instrument Strength Independent of Direct Effect [4]. In general, the InSIDE assumption does not hold if assumption (ii) is violated. But other reasons such as a bidirectional relationship between *X* and *Y* could also cause the violation of InSIDE assumption [5]. Due to both its simplicity and weaker assumptions (in allowing all IVs to have direct effects on the outcome with directional pleiotropy), Egger regression has become one of the most popular MR methods: as one evidence, the number of the citations of one key reference [4] has been increasing every year, totaling over 2000, since its publication in 2015.

Despite its claimed robustness to (uncorrelated) pleiotropy, some authors have noted that Egger regression is dependent on the orientation (or coding) of each SNP/IV [6]. If a (usually biallelic) SNP has two alleles, say an A allele and a G allele, its association value with a trait using A allele as the reference allele would be the opposite of that using G as the reference. Usually we do not expect the analysis conclusion to vary with the coding of a SNP (or any other variables). This property of Egger regression is both surprising and undesirable; we will show in this paper that it is indeed problematic. As to be shown in the real data analysis, when we applied Egger regression to 48 risk factor-disease pairs, the results can be largely different with various orientations or coding schemes of the SNPs being used. One may wonder whether this phenomenon is just due to finite sample sizes. We point out that this problem exists even for large samples; the GWAS sample sizes in our example of the Height-CAD pair are 253288 and 547261 respectively, and the number of IVs used is 986. We will confirm this analytically and via simulations.

Some authors recognized this problem, mainly from its influence on estimating and interpreting the intercept term (i.e. average pleiotropic effect) in the Egger regression model, and thus proposed the *default* orientation of the SNPs so that they are all positively associated with the exposure as recommended and implemented in some popular MR software packages [7, 8], while (implicitly) imposing the InSIDE assumption being satisfied with this *specific* default orientation [6]. We’d argue however that perhaps this problem is not yet as fully and widely appreciated as it should be, including its implications in practice, as the InSIDE assumption being used needs to be clarified. We believe that it is more realistic to assume that the InSIDE assumption holds only for some unknown *oracle* coding; under this assumption, how would various orientations of SNPs impact the causal estimate? If there are biases, are they going to disappear as the sample size increases? In this paper, we will investigate this problem using simulation studies, followed by analytical explanations, and hopefully raise attention to this problem. We show that the problem carries over to similar IV regression methods with individual-level data [9].

## Methods

### Data and model

**Fig 1:**
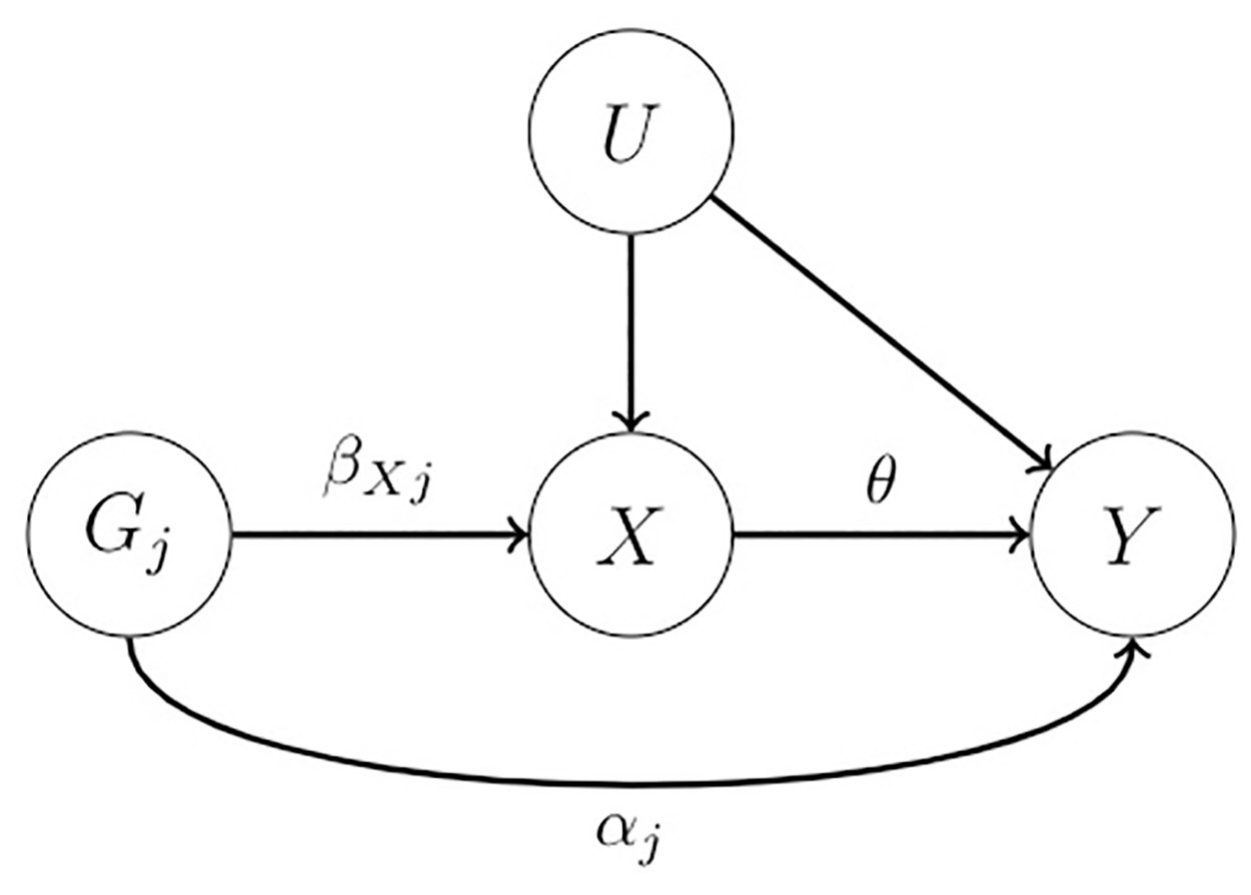
The true causal diagram.

Let $\left({\hat{\beta }}_{Xj},{\hat{\sigma }}_{Xj}\right)$ and $\left({\hat{\beta }}_{Yj},{\hat{\sigma }}_{Yj}\right)$ denote the estimate and its standard error for SNP-exposure and SNP-outcome associations respectively from two independent GWAS summary datasets. By default we assume that the second IV assumption (independence assumption) holds unless specified otherwise. We consider the following true causal model (Fig 1):

$$\begin{array}{cc} X= & \sum_{j=1}^{m}\beta_{Xj}G_{j}+U+\epsilon_{X},\\ Y= & \sum_{j=1}^{m}\alpha_{j}G_{j}+\theta X+U+\epsilon_{Y}, \end{array}$$ (1)

where *ϵ*_*X*_ and *ϵ*_*Y*_ are independent random errors, and (*ϵ*_*X*_, *ϵ*_*Y*_) ⫫ (*G*_1_, …, *G*_*m*_, *U*), *U* is an unmeasured (aggregated) confounder, independent with *ϵ*_*X*_ and *ϵ*_*Y*_; *θ* is the causal effect of interest, and *α*_*j*_ is the direct or pleiotropic effect of *G*_*j*_ on the outcome *Y* not mediated through the exposure *X*. Throughout the paper, we assume that the *m* SNPs are mutually independent. The association between SNP *G*_*j*_ and the outcome *Y*, *β*_*Yj*_, can be decomposed as:

$$\begin{array}{c} \beta_{Yj}=\alpha_{j}+\theta \beta_{Xj}. \end{array}$$ (2)

SNP *G*_*j*_ is an invalid IV with a pleiotropic/direct effect if *α*_*j*_ ≠ 0.

The GWAS summary statistics ${\hat{\beta }}_{Xj}$ and ${\hat{\beta }}_{Yj}$, *j* = 1, …, *m*, are usually computed from simple linear regressions: ${\hat{\beta }}_{Xj}=\hat{\text{cov}}\left(X,G_{j}\right)/\hat{\text{var}}\left(G_{j}\right)$ and ${\hat{\beta }}_{Yj}=\hat{\text{cov}}\left(Y,G_{j}\right)/\hat{\text{var}}\left(G_{j}\right)$, where $\hat{\text{cov}}\left(\right)$ and $\hat{\text{var}}\left(\right)$ are the sample covariance and sample variance respectively. When *G*_1_, …, *G*_*m*_ are mutually independent, we obtain that ${\hat{\beta }}_{Xj}$ and ${\hat{\beta }}_{Yj}$ follow the asymptotic normal distributions with means *β*_*Xj*_ and *β*_*Yj*_ respectively. Given large sample sizes of GWAS as usual, we have

$$\begin{array}{c} {\hat{\beta }}_{Xj}=\beta_{Xj}+\epsilon_{Xj},\;\epsilon_{Xj}∼N\left(0,\sigma_{Xj}^{2}\right),\;j=1,\ldots ,m, \end{array}$$ (3)

$$\begin{array}{c} {\hat{\beta }}_{Yj}=\alpha_{j}+\theta \beta_{Xj}+\epsilon_{Yj},\;\epsilon_{Yj}∼N\left(0,\sigma_{Yj}^{2}\right),\;j=1,\ldots ,m. \end{array}$$ (4)

Throughout the paper, as usual for Egger regression, we impose the no-measurement error (NOME) assumption on the SNP-exposure estimates, i.e., *σ*_*Xj*_ = 0 and ${\hat{\beta }}_{Xj}=\beta_{Xj}$. We also assume that *σ*_*Yj*_ is known or well estimated as ${\hat{\sigma }}_{Yj}$.

### Inverse-variance weighted (IVW) method

The inverse-variance weighted (IVW) method can be viewed as a meta-analysis of the ratio estimates of the causal parameter *θ*, ${\hat{\beta }}_{Yj}/{\hat{\beta }}_{Xj}$, across all SNPs [10]. Under the NOME assumption on SNP-exposure associations, the overall estimator of the causal effect can be obtained by averaging across all *m* SNPs using inverse-variance weighting with weights ${\hat{\beta }}_{Xj}^{2}/{\hat{\sigma }}_{Yj}^{2}$:

$$\begin{array}{c} {\hat{\theta }}_{IVW}=\frac{\sum_{j=1}^{m}{\hat{\beta }}_{Yj}{\hat{\beta }}_{Xj}{\hat{\sigma }}_{Yj}^{-2}}{\sum_{j}{\hat{\beta }}_{Xj}^{2}{\hat{\sigma }}_{Yj}^{-2}}. \end{array}$$ (5)

The same estimator can be also obtained from a weighted linear regression of ${\hat{\beta }}_{Yj}$ on ${\hat{\beta }}_{Xj}$ with weights (${\hat{\sigma }}_{Yj}^{-2}$) and with the intercept constrained to be zero. When there is no heterogeneity in the Wald ratio estimates of *θ* based on the individual IVs, the variance of ${\hat{\theta }}_{IVW}$ is $1/\sum_{j}{\hat{\beta }}_{Xj}^{2}{\hat{\sigma }}_{Yj}^{-2}$, which corresponds to a fixed-effect (FE) meta-analysis, denoted IVW(FE). Otherwise, a multiplicative random-effect model, or IVW(RE), should be preferred and the variance of ${\hat{\theta }}_{IVW}$ is $\sigma_{I}/\sum_{j}{\hat{\beta }}_{Xj}^{2}{\hat{\sigma }}_{Yj}^{-2}$, where *σ*_*I*_ is an overdispersion parameter to be estimated from the residuals in the weighted linear regression described above [6, 11]. In the presence of (balanced) pleiotropy, the over-dispersion parameter (*σ*_*I*_) allows the variance of ${\hat{\theta }}_{IVW}$ to increase so that IVW(RE) could control the type-I error, but the point estimates of IVW(FE) and IVW(RE) are still the same [11].

While requiring the InSIDE assumption, IVW(RE), as another popular MR method closely related to Egger regression, is however invariant to and thus has no problem with various orientations of SNPs. This can be seen from Eq (5) that both the numerator and the denominator are invariant to different orientations of SNPs. This advantage of IVW(RE) and other approaches in modeling the mean of *α*_*j*_’s as 0 has been noted by others [12].

### Egger regression (MR-Egger)

Egger regression (MR-Egger) is a simple modification of IVW(RE) by adding an intercept term to capture the non-zero (weighted) average pleiotropic effect [4]:

$$\begin{array}{c} {\hat{\beta }}_{Yj}=r+\theta {\hat{\beta }}_{Xj}+\epsilon_{Ej};\;\epsilon_{Ej}∼N\left(0,\sigma_{E}^{2}{\hat{\sigma }}_{Yj}^{2}\right), \end{array}$$ (6)

where *ϵ*_*Ej*_ is a random error and $\sigma_{E}^{2}\geq 1$ is an unknown overdispersion parameter (as $\sigma_{I}^{2}$ used in IVW(RE)). The model can be derived from Eqs (3) and (4): under the NOME assumption, we have

$${\hat{\beta }}_{Yj}=\alpha_{j}+\theta {\hat{\beta }}_{Xj}+\epsilon_{Yj}.$$

Treating *α*_*j*_ as random, we have

$$E\left({\hat{\beta }}_{Yj}|{\hat{\beta }}_{Xj}\right)=E\left(\alpha_{j}|{\hat{\beta }}_{Xj}\right)+\theta {\hat{\beta }}_{Xj}=E\left(\alpha_{j}\right)+\theta {\hat{\beta }}_{Xj}=r+\theta {\hat{\beta }}_{Xj},$$

where the second equality follows from the InSIDE assumption, and the third from the definition of *E*(*α*_*j*_) = *r*. Each ${\hat{\beta }}_{Xj}$ is treated as fixed (as a covariate) in fitting the Egger regression Eq (6). It is also clear that other parameters *θ* and *r* are treated as fixed.

When the intercept term *r* is equal to zero, that is, the average pleiotropic effect is zero (known as balanced pleiotropy), the MR-Egger and IVW estimators coincide and both are consistent estimators of the causal effect under the InSIDE assumption, which will be discussed next. When the average pleiotropic effect is not zero (known as directional pleiotropy), it is known that the IVW estimator is not consistent anymore while the MR-Egger estimator is consistent under the InSIDE assumption [4, 6].

### The InSIDE assumption and orientation of SNPs

Throughout this paper, the InSIDE assumption is defined in model Eq (4) for a fixed number of IVs; specifically, the InSIDE assumption holds if the *sample* weighted covariance between the pleiotropic effects and SNP-exposure associations equals to zero for the set of SNPs used in the MR analysis [6, 13]:

$$\begin{array}{c} {\text{cov}}_{w}\left(\alpha ,\beta_{X}\right)=\frac{\sum_{j=1}^{m}{\hat{\sigma }}_{Yj}^{-2}\left(\alpha_{j}-\bar{\alpha }\right)\left(\beta_{Xj}-{\bar{\beta }}_{X}\right)}{\sum_{j=1}^{m}{\hat{\sigma }}_{Yj}^{-2}}=0, \end{array}$$ (7)

where $\bar{\alpha }$ and ${\bar{\beta }}_{X}$ are the weighted sample averages of *α*_*j*_ and *β*_*Xj*_ respectively, with weights equal to $1/{\hat{\sigma }}_{Yj}^{2}$. The MR-Egger estimator is consistent under this InSIDE assumption [6]:

$$\begin{array}{c} {\hat{\theta }}_{Egger}=\frac{{\text{cov}}_{w}\left({\hat{\beta }}_{Y},{\hat{\beta }}_{X}\right)}{{\text{var}}_{w}\left({\hat{\beta }}_{X}\right)}\overset{n\to \infty }{\to }\theta +\frac{{\text{cov}}_{w}\left(\alpha ,\beta_{X}\right)}{{\text{var}}_{w}\left(\beta_{X}\right)}=\theta , \end{array}$$ (8)

where *n* is the sample size of the GWAS data.

In Eq (2), if we use the other allele of SNP *G*_*j*_ as the reference, i.e., if we flip the coding of SNP *G*_*j*_, we will have its associated $\beta_{Xj}^{*}=-\beta_{Xj}$ and $\beta_{Yj}^{*}=-\beta_{Yj}$, and the pleiotropic effect will also change accordingly: $\alpha_{j}^{*}=-\alpha_{j}$. Consequently, the average pleiotropy (i.e., $\bar{\alpha }$), and thus possibly whether there is balanced or directional pleiotropy, will change. That is, the definition of directional pleiotropy depends on the SNP coding. More importantly, it will impact whether the InSIDE assumption holds or not in Eq (4). It is clear that the InSIDE assumption is defined with respect to a *specific* coding scheme of the set of SNPs. With the intercept constrained to be 0 in IVW, flipping the coding of any SNPs will not change the resulting IVW estimate, but it will in general impact the resulting MR-Egger estimates of the causal parameter (i.e. the slope parameter in Eq (6)) as well as the intercept. One exception is when we flip the coding of *all* SNPs, the causal estimate from MR-Egger will remain the same. This issue has been recognized by some authors; accordingly they proposed re-orientating all the SNPs to be positively associated with the exposure [6, 14]. Although this current practice of MR-Egger allows the users to obtain the same causal estimate under the same coding scheme for the same GWAS data, notably it requires the assumption that InSIDE holds for *this* specific default orientation of the SNPs; if this assumption does not hold, MR-Egger may not perform well as to be shown in simulations. In the real analysis, we will show that the results of MR-Egger largely depend on the specific coding being used. We can also see from Eq (8) that the variance of the causal estimate in MR-Egger is inversely proportional to the weighted variance of ${\hat{\beta }}_{X}$, which also depends on the orientation of SNPs.

### The NOME assumption and orientations of SNPs

The standard implementations of IVW and Egger regression as discussed in Inverse-variance weighted (IVW) method and Egger regression (MR-Egger) both assume no-measurement-errors (NOME) for the SNP-exposure associations. However, in practice with finite samples, this could never hold, which can lead to biased causal estimates, even when InSIDE holds. The impact of violation of NOME assumption on IVW and MR-Egger has been studied extensively before [13, 15, 16]. In particular, for an unweighted Egger regression (when ${\hat{\sigma }}_{Yj}^{2}\text{s}$ are the same), as shown in Eq.(3) in [13], $E\left({\hat{\theta }}_{Egger}\right)\approx \theta \text{var}(\beta_{X})/\text{var}({\hat{\beta }}_{X}),$ where var() is the sample variance calculated on the set of *m* IVs used in the analysis. [13] proposed to use *I*^2^ = (*Q* − (*m* − 1))/*Q* to estimate the ratio $\text{var}(\beta_{X})/\text{var}({\hat{\beta }}_{X})$, where $Q=\sum_{j=1}^{m}{\left({\hat{\beta }}_{Xj}-{\bar{\hat{\beta }}}_{X}\right)}^{2}/{\hat{\sigma }}_{Xj}^{2}$ and ${\bar{\hat{\beta }}}_{X}$ is the mean of ${\hat{\beta }}_{X}$ weighted by $1/{\hat{\sigma }}_{Xj}^{2}$. It is clear that the degree of NOME violation in MR-Egger also depends on the orientations of SNPs. As ${\hat{\beta }}_{Xj}$’s are more widely dispersed, *I*^2^ is closer to one, and the impact of NOME violation is smaller. However, the default coding makes all ${\hat{\beta }}_{Xj}$ to be positive and thus *I*^2^ would be the smallest among all SNP coding schemes. As to be shown later in simulations, this will lead to larger biases in the causal estimates using the default coding even when the InSIDE assumption is satisfied.

### Radial Egger regression

A closely related method, Radial-Egger [17], introduces a new form of MR-Egger:

$$\begin{array}{c} {\hat{\theta }}_{j}\sqrt{w_{j}}=r^{\prime }+\theta \sqrt{w_{j}}+\epsilon_{Rj};\;\epsilon_{Rj}∼N\left(0,\sigma_{R}^{2}\right), \end{array}$$

where ${\hat{\theta }}_{j}={\hat{\beta }}_{Yj}/{\hat{\beta }}_{Xj}$ and we used the first-order weights $w_{j}={\hat{\beta }}_{Xj}^{2}/{\hat{\sigma }}_{Yj}^{2}$. To derive the above model, as for MR-Egger, we start from the true model Eqs (3) and (4) with the NOME assumption, obtaining

$${\hat{\theta }}_{j}\sqrt{w_{j}}=\alpha_{j}\sqrt{w_{j}}/{\hat{\beta }}_{Xj}+\theta \sqrt{w_{j}}+\epsilon_{Yj}\sqrt{w_{j}}/{\hat{\beta }}_{Xj},$$

$$E\left({\hat{\theta }}_{j}\sqrt{w_{j}}|\sqrt{w_{j}}\right)=E\left(\alpha_{j}\sqrt{w_{j}}/{\hat{\beta }}_{Xj}|\sqrt{w_{j}}\right)+\theta \sqrt{w_{j}},$$

which reduces to the Radial-Egger regression model if

$$E\left(\alpha_{j}\sqrt{w_{j}}/{\hat{\beta }}_{Xj}|\sqrt{w_{j}}\right)=E\left(\alpha_{j}\text{sign}\left({\hat{\beta }}_{Xj}\right){\hat{\sigma }}_{Yj}^{-1}|\sqrt{w_{j}}\right)=E\left(\alpha_{j}\text{sign}\left({\hat{\beta }}_{Xj}\right){\hat{\sigma }}_{Yj}^{-1}\right)=r^{\prime },$$

where the second equality requires a new form of the InSIDE assumption that the (weighted) pleiotropic effects (with respect to the default exposure-increasing coding as adopted here) are independent of the Radial weights [17] (and as usual assuming that ${\hat{\sigma }}_{Yj}^{-1}$ is fixed). This InSIDE assumption is similar to the one used (for the default coding) in MR-Egger. As to be shown in the simulation, Radial-Egger indeed performed similarly to MR-Egger with the default coding.

### GWAS summary data

We examined the SNP coding issue of Egger regression on 48 risk factor-disease pairs, including 12 cardiometabolic risk factors and 4 diseases. The 12 risk factors were triglycerides (TG), low-density lipoprotein cholesterol (LDL), high-density lipoprotein cholesterol (HDL) [18], Height [19], body-mass index (BMI) [20], body fat percentage (BF) [21], birth weight (BW) [22], diastolic blood pressure (DBP), systolic blood pressure (SBP) [23], fasting glucose (FG) [24], Smoke and Alcohol [25]. The 4 diseases were coronary artery disease (CAD) [26], stroke [27], type 2 diabetes (T2D) [28] and asthma [29].

The sample sizes for the 16 GWAS datasets ranged from 46 186 to 1 232 091, with a median of 220 933. The numbers of IVs used in the 48 MR analyses ranged from 9 to 1345, with a median of 126.

### Simulation set-ups

We simulated data according to the true causal model Eq (1) with *G*_*j*_ ∼ Binomial(2, 0.3), and $U,\epsilon_{X},\epsilon_{Y}∼N\left(0,1\right)$ independently. We simulated *β*_*Xj*_ from (a) a uniform distribution on (−0.2, −0.1) ∪ (0.1, 0.2); (b) a uniform distribution on (−0.1, −0.03) ∪ (0.1, 0.2) and (c) a uniform distribution on (0.1, 0.3). We will refer them to Simulation (a), (b) and (c) respectively later. In all three simulation set-ups, we considered 0%, 30%, 70% or 100% invalid IVs with balanced pleiotropy $\alpha_{j}∼N\left(0,0.1^{2}\right)$, or with directional pleiotropy $\alpha_{j}∼N\left(0.1,0.1^{2}\right)$. It is noted that for each simulation set-up, we generated IV strengths *β*_*Xj*_’s and direct effects *α*_*j*_’s from *independent* distributions. Although for each specific simulated dataset, the sample covariance of *β*_*Xj*_ and *α*_*j*_ (*j* = 1, …, *m*) (Eq (7)) might not be exactly equal to zero, across all simulated datasets, the average sample covariance between *β*_*Xj*_ and *α*_*j*_ will be (nearly) zero (see S1 Text). Following [13], we refer this as *‘weak’* InSIDE assumption.

The causal effect *θ* was set to 0 or 0.2, and the number of IVs, *m*, to 30 or 100. For Simulation (b), we also considered 500 IVs. The summary data for genetic associations were calculated for the exposure and the outcome on non-overlapping samples of individuals, each consisting of *n* = 50 000 or 100 000 individuals. The *oracle* coding referred to the specific coding that was used to generate the simulated data under the (weak) InSIDE assumption.

In Simulation (a), the mean of SNP-exposure associations was zero, a special case we will discuss later; Simulation (b) was more general with both positive and negative SNP-exposure associations with a non-zero mean; Simulation (c) was also a special case with all SNP-exposure associations being positive. Here the SNP-exposure associations as well as the pleiotropy were defined with respect to the *oracle* coding scheme.

We ran 1000 replications for each simulation set-up. For each simulated dataset, we applied MR-Egger with (i) the *default* coding (as adopted in the current practice): we orientated SNPs so that ${\hat{\beta }}_{Xj}$ were all positive in Eq (6); (ii) the *oracle* coding: we used the coding generating the simulated data, under which the weak InSIDE assumption was satisfied; (iii) the *random* coding: we randomly flipped the coding of some SNPs. We also applied IVW(RE) and Radial-Egger for comparison.

## Results

### Real data example

**Fig 2:**
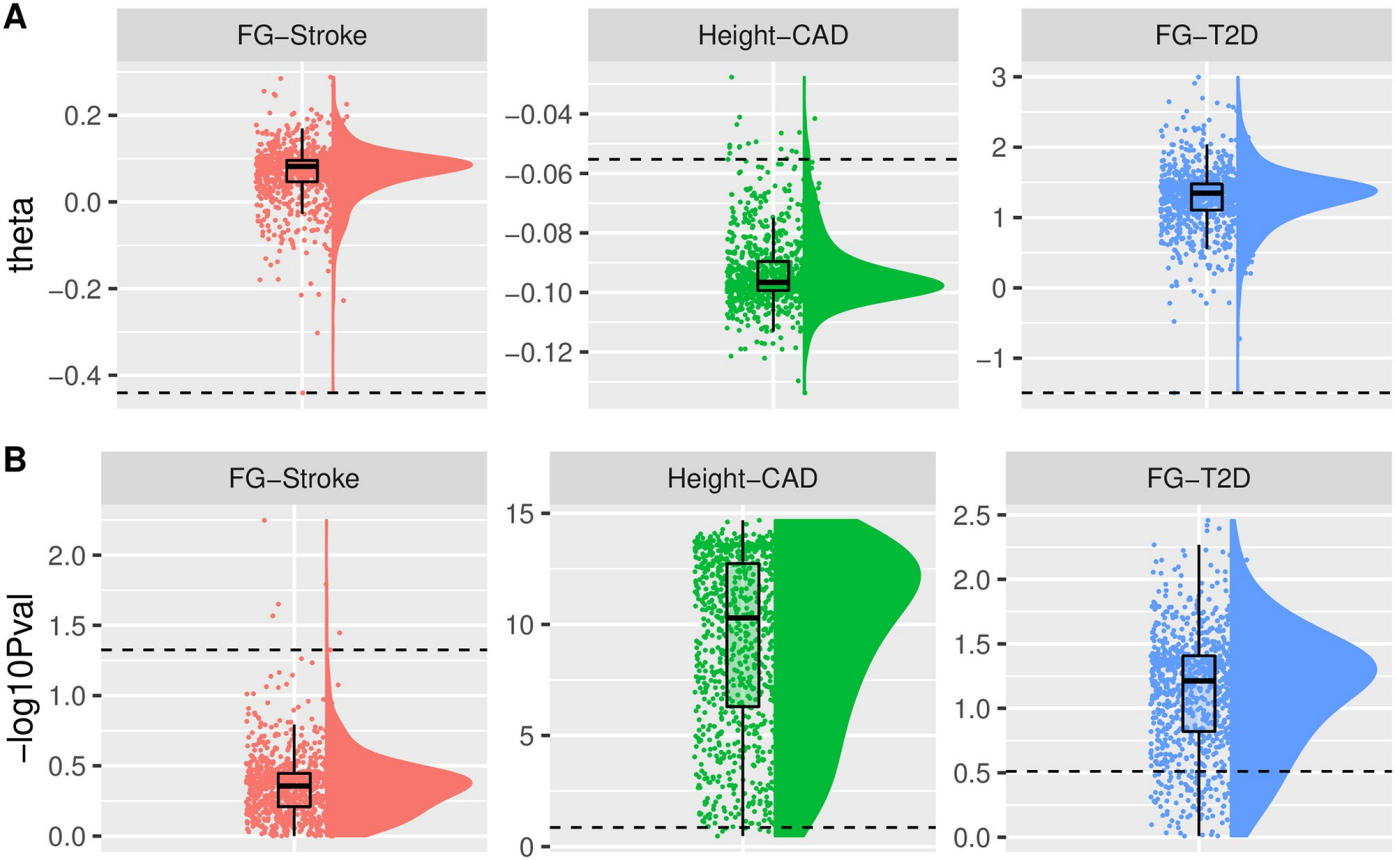
Three real data examples showing different results from different coding schemes in Egger regression. Panel A: estimates of the causal effect; Panel B: -log_10_(p-value)’s of the causal effect. The dashed line in each plot corresponds to the result from the *default* coding.

As a motivating example, we applied MR-Egger to some large-scale GWAS summary data of 48 risk factor-disease pairs [30] using the *default* coding scheme (i.e., we orientated the SNPs so that they were all positively associated with the exposure as recommended and implemented in the popular TwoSampleMR software [7]) and a *random* coding scheme (i.e. we randomly selected the reference allele for each SNP). With the default coding, Egger regression identified 7 significant pairs, whereas with the whatever coding given in the original GWAS datasets from [30], Egger regression identified 17 significant pairs (after the Bonferroni correction). Fig 2 shows some representative results for three pairs: Fasting glucose (FG)-Stroke, body height-coronary artery disease (CAD) and FG-type 2 diabetes (T2D), in which we tried 999 random (and unique) codings plus the default one (as the dashed lines in the plot). We can see that with different coding schemes in Egger regression, not only the point estimates of the causal parameter varied (even from negative to positive), but also the p-values (from insignificant to highly significant), giving possibly opposite conclusions. For example, for the FG-Stroke pair, using the default coding suggested a negative relationship ($\hat{\theta }=-0.44$ with p-value = 0.047) while using the original coding in the GWAS dataset suggested a positive relationship ($\hat{\theta }=0.17$ with p-value = 0.010). These results clearly demonstrate the critical and possibly dramatic dependence of MR-Egger on the orientation of SNPs.

We also investigated whether a larger intercept estimate (in absolute value) was obtained when the default (i.e. exposure-increasing) coding was used in MR-Egger. We found that using the default coding often yielded more extreme intercept estimates than using other (random) coding schemes, but not necessarily more significant p-values (because the standard errors of the intercept estimates were usually larger under the default coding). The details are given in S1 Text.

### Simulation results

#### Simulation (a): SNP-exposure associations with mean 0

**Fig 3:**
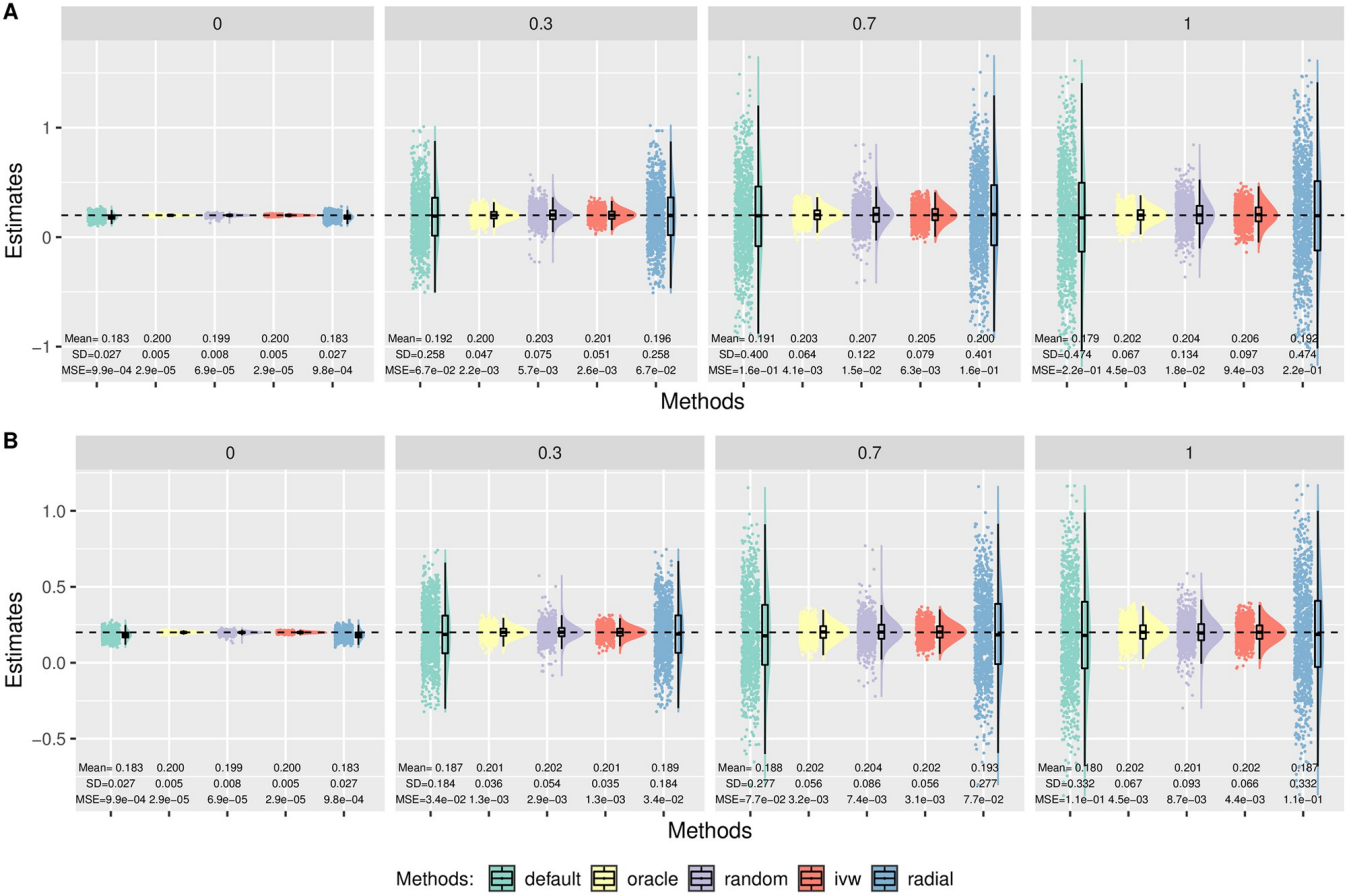
Simulation (a) results with *n* = 100 000, *θ* = 0.2, *m* = 100. Empirical distributions of the estimates of the causal effect *θ* by the methods. Each column corresponds to 0%, 30%, 70% or 100% invalid IVs. A: Directional pleiotropy. B: Balanced pleiotropy.

In Simulation (a), we generated *β*_*Xj*_ from a uniform distribution on (−0.2, −0.1) ∪ (0.1, 0.2). Here we only show some representative results while others are given in S1 Text. Fig 3 shows the distributions of the causal estimates by each method with *m* = 100, *n* = 100 000, *θ* = 0.2 in the presence of directional (Panel A) and balanced pleiotropy (Panel B). First, MR-Egger with the oracle coding performed the best with unbiased estimates and smallest variances across all different scenarios. In the case of balanced pleiotropy, MR-Egger with the oracle coding and IVW coincided with each other as expected. In the meantime, MR-Egger with the default coding gave slightly biased estimates towards the null. This was perhaps due to the violation of NOME assumption with the average estimated *I*^2^ statistic about 0.921. On the other hand, the average estimated *I*^2^ statistic was 0.997 with the oracle coding. (More simulation results studying the NOME assumption are given in S1 Text.) Despite the slight bias due to violation of NOME assumption, we note that the (weak) InSIDE assumption still held under the default coding in this simulation set-up, which will be shown in Analysis. As for Radial-Egger, we can see that it performed similarly to the default coding in MR-Egger. Also, perhaps surprisingly, IVW yielded the unbiased estimates even in the case of directional pleiotropy (Fig 3A), and we will show the reason later. In addition, even though the (weak) InSIDE assumption still held, using the default coding magnified the extent of the violation of NOME assumption, leading to larger finite-sample biases. Finally, MR-Egger with the default coding yielded the largest variance for the causal estimate, implying its low power.

#### Simulation (b): SNP-exposure associations with a non-zero mean

**Fig 4:**
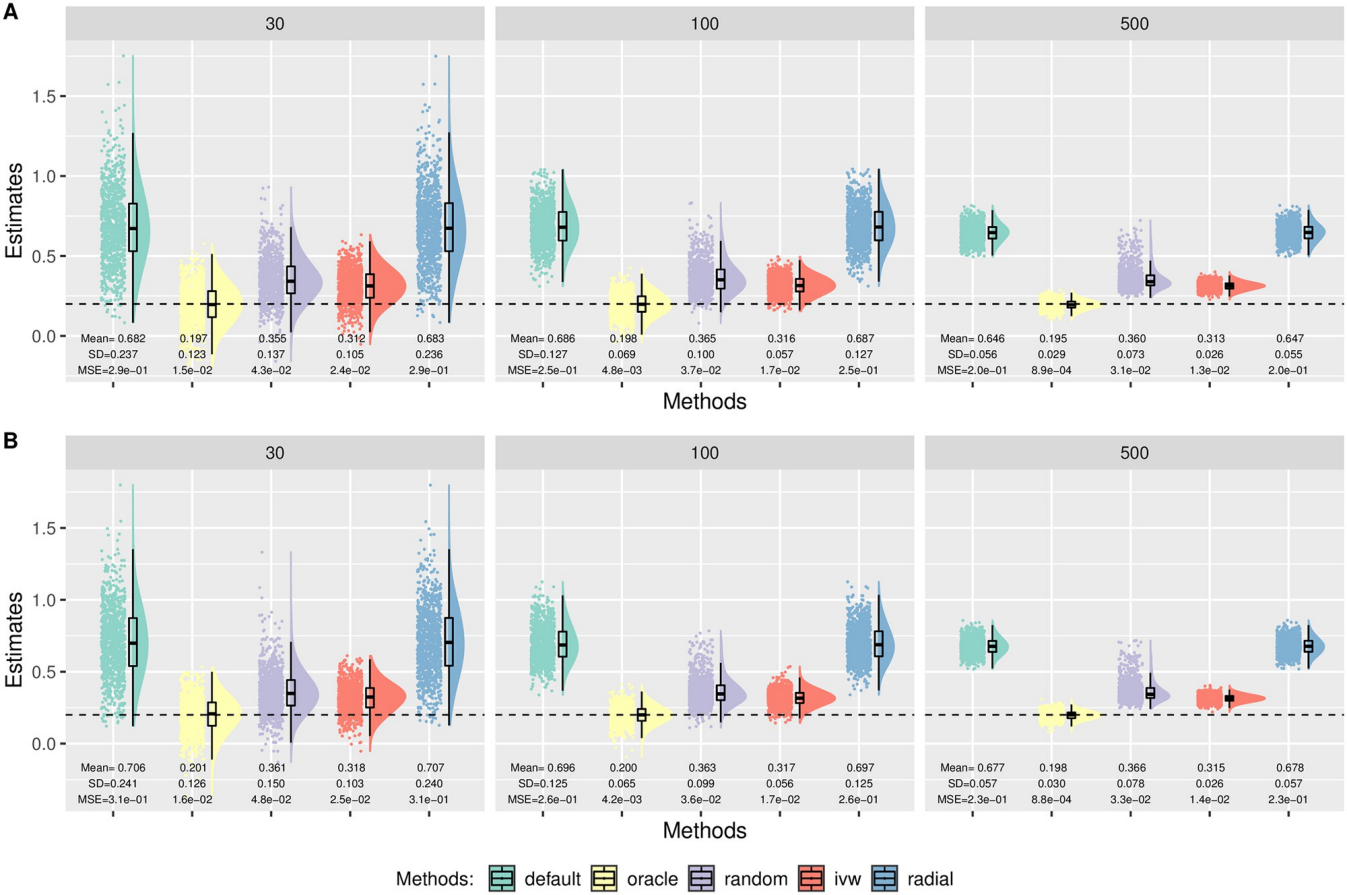
Simulation (b) results with directional pleiotropy. Empirical distributions of the estimates of the causal effect *θ* by the methods with 30% invalid IVs and *θ* = 0.2. Each column corresponds to *m* = 30, 100 or 500 IVs. A: *n* = 50 000. B: *n* = 100 000.

In Simulation (b), we generated *β*_*Xj*_ from a uniform distribution on (−0.1, −0.03) ∪ (0.1, 0.2). Fig 4 shows the distributions of the causal estimates by each method with *θ* = 0.2 in the presence of directional pleiotropy with 30% invalid IVs. The top row corresponds to *n* = 50 000 and the bottom corresponds to a larger sample size of *n* = 100 000. As we can see, only MR-Egger with the oracle coding gave unbiased estimates. Radial-Egger and MR-Egger with the default coding performed similarly with the largest bias. Moreover, the bias did not disappear as the sample size and the number of IVs increased. This result may seem to contradict the common belief that MR-Egger is robust to directional pleiotropy (under InSIDE), but we will show later that the current practice of flipping SNPs actually led to the violation of the InSIDE assumption in this scenario, thus yielding the biased causal parameter estimates. Furthermore, this was *not* due to the violation of the NOME assumption either. When we used the true *β*_*Xj*_, instead of the estimated ${\hat{\beta }}_{Xj}$, the bias still persisted for the default coding in MR-Egger; the details are given in S1 Text.

**Fig 5:**
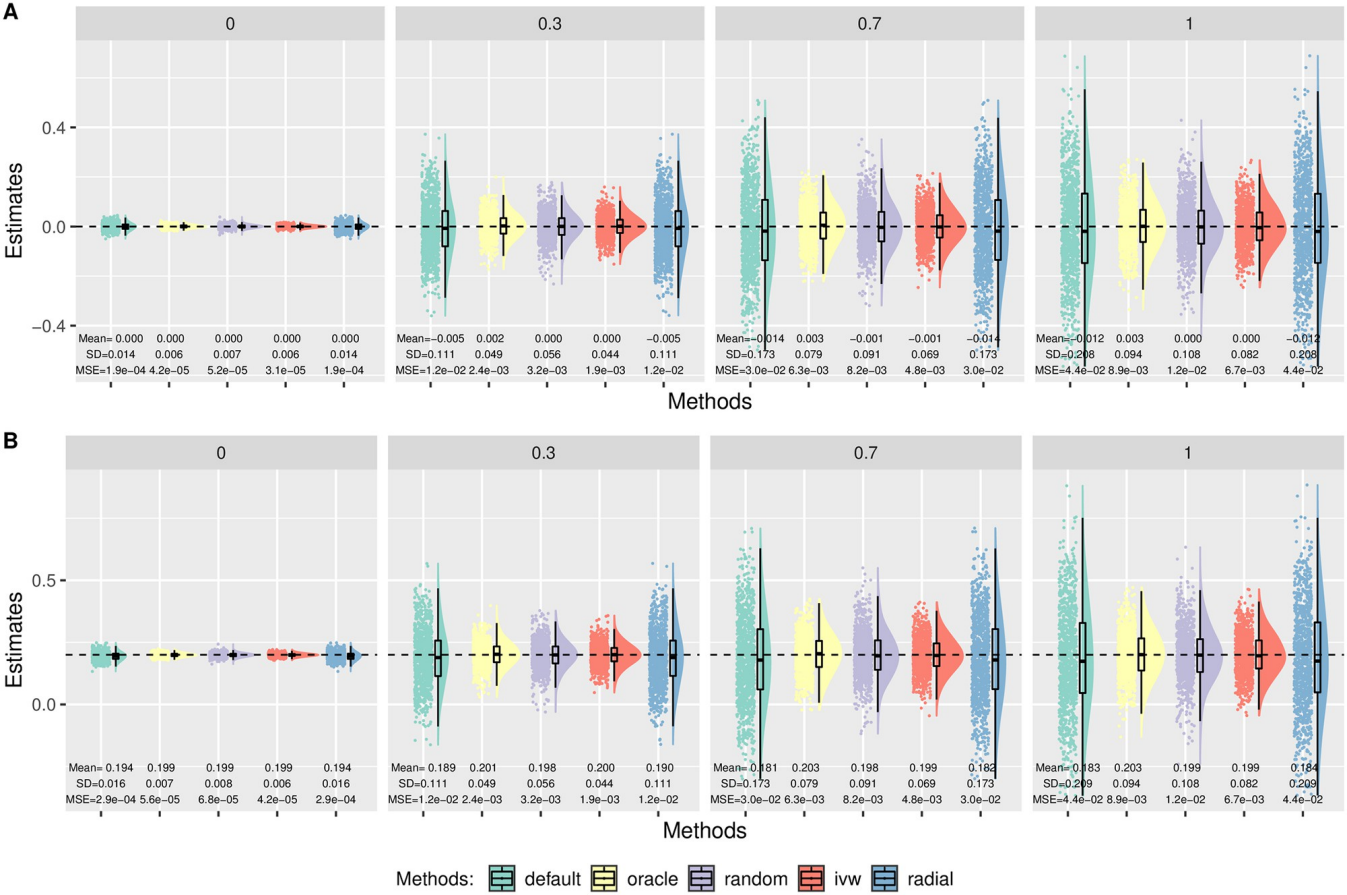
Simulation (b) results with balanced pleiotropy, *n* = 100 000, *m* = 100. Empirical distributions of the estimates of the causal effect *θ* by the methods. Each column corresponds to 0%, 30%, 70% or 100% invalid IVs. A: *θ* = 0. B: *θ* = 0.2.

Fig 5 shows the results with *m* = 100 and *n* = 100 000 in the case of balanced pleiotropy. In this case, MR-Egger with the oracle coding and IVW yielded unbiased estimates with the smallest variance. Again, though the (weak) InSIDE assumption held under the default coding here (with balanced pleiotropy) as to be shown in Analysis, it yielded slight under-estimation perhaps due to the violation of NOME.

#### Simulation (c): All positive SNP-exposure associations

**Fig 6:**
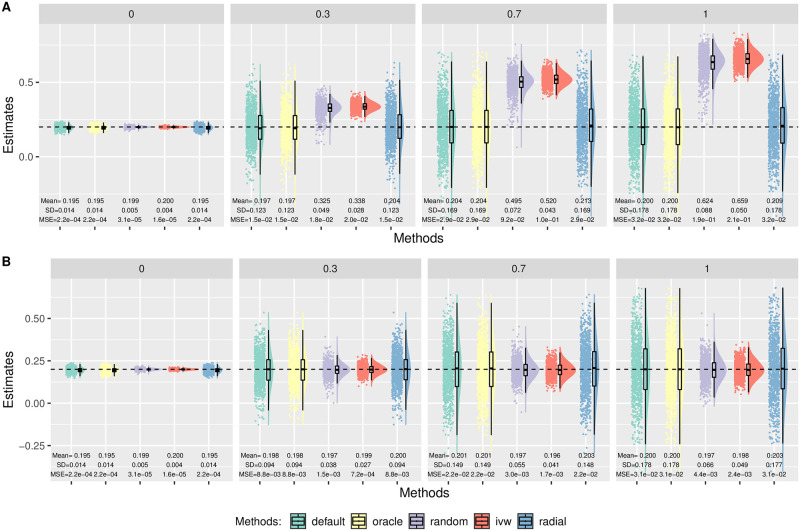
Simulation (c) results with *n* = 100 000, *θ* = 0.2, *m* = 100. Empirical distributions of the estimates of the causal effect *θ* by the methods. Each column corresponds to 0%, 30%, 70% or 100% invalid IVs. A: Directional pleiotropy. B: Balanced pleiotropy.

In Simulation (c), we generated *β*_*Xj*_ to be all positive from a uniform distribution on (0.1, 0.3). As a result, the default coding coincided with the oracle coding in this case. As shown in Fig 6, the default and oracle codings in MR-Egger had the same results with approximately unbiased estimates, as expected. Again, Radial-Egger gave the similar results to that of the default coding in MR-Egger. On the other hand, IVW yielded biased estimates in the presence of directional pleiotropy (panel A), but gave unbiased estimates with balanced pleiotropy (panel B).

### Analysis

In this session, we will dive into the relationship between the orientation of SNPs and the InSIDE assumption, and the impact on the IVW and MR-Egger estimates of the causal parameter. For simplicity of notation, we assume that the *m* SNPs have the same minor allele frequency so that ${\hat{\sigma }}_{Yj}^{2}$ are the same and thus no need to use the weighted covariance in the definition of InSIDE; a similar argument carries over for the general case. First, the InSIDE assumption in Eq (7) for the oracle coding becomes:

$$\begin{array}{c} m\cdot \text{cov}\left(\alpha ,\beta_{X}\right)=\sum_{j=1}^{m}\alpha_{j}\beta_{Xj}-m\bar{\alpha }{\bar{\beta }}_{X}=0 \end{array}$$ (9)

If we flip the coding of some SNPs, say the first 0 < *k* < *m* SNPs, and denote the new data as $\left(\beta_{Xj}^{*},\beta_{Yj}^{*}\right)$. That is, for *j* = 1, …, *k*, $\beta_{Xj}^{*}=-\beta_{Xj}$, $\beta_{Yj}^{*}=-\beta_{Yj}$ and $\alpha_{j}^{*}=-\alpha_{j}$, while for others, $\beta_{Xj}^{*}=\beta_{Xj}$, $\beta_{Yj}^{*}=\beta_{Yj}$ and $\alpha_{j}^{*}=\alpha_{j}$. Then in general we have

$$\begin{array}{c} m\cdot \text{cov}\left(\alpha^{*},\beta_{X}^{*}\right)=\sum_{j=1}^{m}\alpha_{j}^{*}\beta_{Xj}^{*}-m{\bar{\alpha }}^{*}{\bar{\beta }}_{X}^{*}=\sum_{j=1}^{m}\alpha_{j}\beta_{Xj}-m{\bar{\alpha }}^{*}{\bar{\beta }}_{X}^{*}\neq 0, \end{array}$$ (10)

where the last inequality is due to $\sum_{j}\alpha_{j}\beta_{Xj}=m\bar{\alpha }{\bar{\beta }}_{X}\neq m{\bar{\alpha }}^{*}{\bar{\beta }}_{X}^{*}$ unless under some special situations. This suggests that the default coding or an arbitrarily chosen coding in MR-Egger is likely to lead to the violation of the InSIDE assumption, and by (8) and (10), to inconsistent estimates.

In general, the (asymptotic) bias of the MR-Egger estimate will not diminish even for a large *m* after flipping the coding of *k* SNPs on the basis of the oracle coding. Under the theoretical model that both *α*_*j*_’s and *β*_*Xj*_’s are iid from two continuous distributions each of a bounded domain and non-zero mean, and that *k*/*m* → *c* ∈ (0, 1) as *m* → ∞. It is easy to verify that as *m* → ∞, we have $\bar{\alpha }\to \mu_{\alpha }$ say, and ${\bar{\alpha }}^{*}\to \left(1-2c\right)\mu_{\alpha }\neq \mu_{\alpha }$, and similarly for ${\bar{\beta }}_{X}$ and ${\bar{\beta }}_{X}^{*}$. Then with probability one we have $\bar{\alpha }{\bar{\beta }}_{X}-{\bar{\alpha }}^{*}{\bar{\beta }}_{X}^{*}↛0$ as *m* → ∞.

In contrast, the IVW estimate in Eq (5) after flipping the coding becomes

$$\begin{array}{c} {\hat{\theta }}_{IVW}^{*}=\frac{\sum_{j=1}^{m}{\hat{\beta }}_{Yj}^{*}{\hat{\beta }}_{Xj}^{*}}{\sum_{j=1}^{m}{\left({\hat{\beta }}_{Xj}^{*}\right)}^{2}}=\frac{\sum_{j=1}^{m}{\hat{\beta }}_{Yj}{\hat{\beta }}_{Xj}}{\sum_{j=1}^{m}{\hat{\beta }}_{Xj}^{2}}={\hat{\theta }}_{IVW}\overset{n\to \infty }{\to }\theta +\frac{\sum_{j=1}^{m}\alpha_{j}\beta_{Xj}}{\sum_{j=1}^{m}\beta_{Xj}^{2}}, \end{array}$$ (11)

showing that the IVW estimate is invariant to re-orientation of SNPs, and it is consistent when ∑_*j*_ *α*_*j*_ *β*_*Xj*_ is zero. Under the InSIDE assumption in Eq (9), this holds when $\bar{\alpha }=0$, i.e., balanced pleiotropy, and/or ${\bar{\beta }}_{X}=0$. The latter condition explains why IVW still yielded unbiased estimates even in the case of directional pleiotropy in Simulation (a), where we generated *β*_*Xj*_ from a uniform distribution on (−0.2, −0.1) ∪ (0.1, 0.2).

In summary, the key issue here is that even though the InSIDE assumption holds for some unknown oracle coding, in general it does not for the default coding or an arbitrarily chosen coding, leading to inconsistent estimates in MR-Egger. Notably the InSIDE assumption is difficult to check [31, 32].

### The problem remains with the use of individual-level data

Instead of applying MR to GWAS summary data, one can apply IV regression to model (1) with individual-level data. With high-dimensional IVs, i.e. a large *m*, the InSIDE assumption is required [9]: $\text{cov}(\alpha ,\beta_{X})=0$ (or more generally, → 0 as *m* → ∞) for some oracle coding. Using the same argument as before, if the SNPs/IVs are recoded with the corresponding $\alpha^{*}$ and $\beta_{X}^{*}$, under general conditions we have $\text{cov}(\alpha^{*},\beta_{X}^{*})\neq 0$ (or ↛ 0), leading to the violation of the InSIDE assumption and thus an inconsistent estimate. Corresponding to MR-Egger, we can implement the 2-stage IV regression by imposing $\alpha_{j}∼N\left(r,\sigma_{\alpha }^{2}\right)$ iid. As shown in S1 Text, it was confirmed in the simulations that when applied to individual-level data, such an IV regression method behaved similarly to MR-Egger, yielding biased estimates of *θ* for non-oracle coding schemes. In contrast, the method imposing *r* = 0 performed similarly to IVW(RE), invariant to re-orientations of the SNPs.

### Results for other related methods

We note that other methods that incorporate Egger regression, such as MV-MR-Egger [14], LDA MR-Egger [33], MV-IWAS-Egger [34], PMR-Egger [35], mixIE [36], strictly speaking, would also inherit the limitations of MR-Egger, but possibly to varying extents. We applied both PMR-Egger and mixIE to simulated data as detailed in S1 Text. Under some general conditions PMR-Egger might not perform well. On the other hand, mixIE was much more robust to various orientations of SNPs except when the proportion of invalid IVs was extremely high (e.g. close to 100%), which might be rare in practice. This is because mixIE depends on the IVW estimate (based on detected valid IVs) to a larger degree than on the MR-Egger estimate (based on detected invalid IVs), and often it could correctly identify valid IVs. Furthermore, mixIE is also more robust to mild to moderate violations of the InSIDE assumption [36].

We also applied some robust MR methods that are invariant to allele coding, including MR-cML [37] and MR-RAPS [12]. In simulations these methods performed well when the proportion of invalid IVs were not high; otherwise, e.g. when all IVs were invalid, only MR-Egger (with the oracle coding) performed well. The results are shown in S1 Text.

### Irrelevant IVs

Albeit not the main point here, we point out a related issue that MR-Egger, or more specifically the InSIDE assumption, is not robust to the presence of irrelevant IVs (i.e. with the IV relevance assumption violated). Suppose that we mistakenly use *m*_0_ ≥ 1 irrelevant IVs with *β*_*Xj*_ = 0 for *j* = *m* + 1, …, *m* + *m*_0_, in addition to *m* IVs used before. Even if the oracle coding is known and the InSIDE holds for the first *m* IVs, we have

$$\begin{array}{c} \left(m+m_{0}\right)\text{cov}\left(\alpha ,\beta_{X}\right)=\sum_{j=1}^{m+m_{0}}\alpha_{j}\beta_{Xj}-\left(m+m_{0}\right)\bar{\alpha }{\bar{\beta }}_{X}=\sum_{j=1}^{m}\alpha_{j}\beta_{Xj}-\left(m+m_{0}\right)\bar{\alpha }{\bar{\beta }}_{X}\neq 0, \end{array}$$ (12)

because, by (9), $\sum_{j=1}^{m}\alpha_{j}\beta_{Xj}=\left(\sum_{j=1}^{m}\alpha_{j}\right)\left(\sum_{j=1}^{m}\beta_{Xj}\right)/m\neq \left(\sum_{j=1}^{m+m_{0}}\alpha_{j}\right)\left(\sum_{j=1}^{m}\beta_{Xj}\right)/\left(m+m_{0}\right)$ in general unless under special cases such as $\sum_{j=1}^{m}\beta_{Xj}=0$, or $\sum_{j=1}^{m}\alpha_{j}=\sum_{j=1}^{m+m_{0}}\alpha_{j}=0$. Note that this conclusion holds regardless whether the irrelevant SNPs have direct effects or not (unless under some special cases). Hence, in general the InSIDE assumption would be violated if all *m* + *m*_0_ IVs are used, leading to an inconsistent estimate; this was confirmed in the simulations detailed in S1 Text. Section G. This non-robustness property of MR-Egger is in contrast to some other methods, such as MR-RAPS [12] and MR-cML [37], whose consistency will not be influenced by the presence of a few irrelevant IVs (without and with pleiotropy respectively); see the conditions for Theorem 3.3 in [12], which also holds for cML. This is relevant because some authors [12] have advocated using various larger sets of IVs, possibly including some weak or irrelevant IVs, to increase the estimation efficiency and assess the robustness of a causal conclusion in an MR analysis, including from MR-Egger [38].

### Testing the intercept in MR-Egger

We also performed simulations to study the performance of testing the intercept term with the null hypothesis *H*_0_: *r* = 0 versus *H*_1_: *r* ≠ 0 in MR-Egger Eq (6) using different SNP coding schemes. This test can be useful in suggesting the presence of invalid IVs with pleiotropic effect. We summarize our main findings here with all details given in S1 Text. Section H. First, when all IVs are valid, the intercept term in Eq (6) is expected to be zero, no matter what coding scheme is used. Thus using any coding scheme could maintain a correct type I error rate. Second, the estimated intercept in MR-Egger based on the default coding tended to be larger (in absolute values) than those using other coding schemes, but the power could still be relatively low because of the lower precision of the estimate. Third, a non-zero intercept could mean several different things, such as the presence of correlated pleiotropy, the presence of uncorrelated directional pleiotropy, or both. In general the intercept term should not be simply interpreted as the average pleiotropic effect in practice [6]. As shown in Section H.3 in S1 Text, in the presence of correlated pleiotropy, even in the scenario where the pleiotropic effects of all invalid IVs were positive under the default coding, the estimated intercept could still be negative.

## Discussion

Although the phenomenon that MR-Egger depends on the coding of SNPs has been noticed and the default coding as a remedy has been recommended and widely applied [6, 14], to our best knowledge, there has been no other assessment and analysis of its implications and impact. In this paper, we have examined the influence of SNPs’ coding on MR-Egger. Our findings could be summarized as follows. First, the current practice of orientating SNPs to be all positively associated with the exposure (referred as the default coding in MR-Egger) will be problematic unless its corresponding and coding-specific InSIDE assumption holds. Assuming that there is a true oracle coding under which the InSIDE assumption holds, the InSIDE assumption under the default (or another) coding will still hold and the current practice will yield a consistent estimate in the case of balanced pleiotropy (with respect to the oracle coding) and/or the SNP-exposure associations under the oracle coding have mean zero. When the SNP-exposure associations under the oracle coding have the same sign (all positive or negative), the default coding coincides with the oracle coding, thus will also give a consistent estimate; this is what is imposed in the current practice of MR-Egger, different from that the InSIDE assumption holds for some unknown oracle coding, under which, more generally and more likely, the current practice of applying MR-Egger will yield biased estimates. We also point out that this is not a finite-sample problem. In addition to our analysis, we have shown in the motivating real data examples and simulation studies that even with large *n* (and *m*), this issue persisted. Second, even in the (special and unlikely) case when using the default coding in MR-Egger could still give consistent estimates, its variance is usually large because of the small ranges of SNP-exposure association effects after reorientation [6]. This also contributes to the low power with the default coding as noticed previously [37]. A small range of SNP-exposure associations would also magnify the degree of NOME violation, leading to a larger bias. Third, compared with another popular method IVW(RE), as shown in our simulation studies and analysis, the current practice of MR-Egger would only have an advantage when the default coding is the oracle coding and there is directional pleiotropy with respect to the default (oracle) coding. Fourth, most importantly, in practice we don’t know the oracle coding under which the InSIDE assumption holds (if so) and the InSIDE assumption is very difficult to test, hence cautions should be taken when applying MR-Egger.

One may wonder whether the oracle coding, under which the InSIDE assumption holds, can be identified in practice. We tried a model selection approach to select the “best” coding for the Egger regression model (Eq (6)), but it did not always work, sometimes not only failing to select the best model but also leading to inflated type I errors. Alternatively, we tried to choose a coding scheme giving the minimum evidence of the violation of the InSIDE assumption. This turned out to be quite challenging too, involving a circular reasoning—to test whether a coding scheme under which the InSIDE assumption is satisfied requires a reliable/valid causal estimate, which however relies on the InSIDE assumption. It is noted that, under any non-oracle coding, although MR-Egger may give a biased estimate, the corresponding regression model is indeed “correct” in the usual sense that the specified regression model may still fit well the given data. A possible approach is to use one of other robust MR methods that do not require the InSIDE assumption to obtain a reliable causal estimate, such as MR-cML [37] and MRMix [39]. Then we could use this estimate to assess the InSIDE assumption for a given coding of SNPs. However, those methods have their own assumptions. In particular, when all the SNPs are invalid with directional pleiotropy, those methods all break down, while MR-Egger with the oracle coding still works, as shown in S1 Text. Furthermore, such a practice appears unnecessary if we feel confident in having already obtained a reliable causal estimate via another method.

Treating some nuisance parameters as random is a common and often effective way to reduce the number of the parameters to be estimated. For example, by modeling the direct effects *α*_*j*_’s as Normal random effects in MR-Egger, we do not need to estimate them but their mean *r* (and variance). In longitudinal and clustered data analysis, subject-specific effects are modeled as random in generalized linear mixed-effects models (GLMMs). However, modeling nuisance parameters as random usually imposes another important but largely neglected assumption: the distribution of the random effects is independent of other covariates; in MR-Egger, the other covariates are SNP-exposure associations ${\hat{\beta }}_{Xj}$, and the assumption is equivalent to the InSIDE assumption. The violation of this assumption can happen, leading to biased estimates in GLMMs [40]. [40] also showed that, by treating the subject-specific effects as fixed, instead of random, then applying a conditional likelihood approach (that eliminates the subject-specific effects from the conditional likelihood by conditioning on their sufficient statistics) avoids the problem. In the current context, if we treat the direct effects as fixed, the model is over-specified and the parameters are not estimable while it is unclear how to apply a conditioning argument; however, under other assumptions, notably that some *α*_*j*_ = 0 in the framework of MR-cML, one can allow the violation of the InSIDE assumption (and more generally allow correlated pleiotropy) for a subset of the IVs [37]. Furthermore, with a mean zero assumption on the random effects in GLMMs and IVW(RE), there is no issue of the dependence of the result on the coding of the covariates/SNPs; in contrast, it becomes problematic by assuming a non-zero mean of the random effects in MR-Egger.

In summary, we have studied the impact of SNP coding/orientation on Egger regression (and similar IV regression methods requiring the InSIDE assumption [9]). We emphasize that, since the InSIDE assumption is defined with respect to a specific coding scheme of the SNPs, even if it holds for some unknown (oracle) coding scheme, generally it does not hold for the default (exposure-increasing) coding (and many other codings) unless under some special and unlikely scenarios (such as when the default coding coincides with the oracle coding). The violation of the InSIDE assumption leads to the inconsistent estimator of the causal effect in MR-Egger. Thus, it is important for practitioners to keep in mind that, when applying MR-Egger with the default exposure-increasing allele coding, the interpretation of the causal effect estimate depends crucially on the non-violation of the InSIDE assumption under the default coding. We suggest that this SNP coding-specific assumption should be stated clearly when interpreting the results. How to fix the problem does not appear obvious since the InSIDE assumption is difficult to test and selecting the oracle coding is challenging. We have also confirmed that SNP coding in MR-Egger impacts the precision of the causal estimate as well as the extent of the NOME violation. The default coding gives the smallest range of IV-exposure associations, which tends to increase the variance of the causal estimate and magnify the degree of the NOME violation. Moreover, MR-Egger is not robust to outliers due to its use of the squared error loss function; it will be more robust to use other robust loss functions [12]. Until a better solution appears, we should be cautious when applying MR-Egger (and other related MR and IV regression methods for either GWAS summary or individual-level data) in data analysis.

## Supporting information

S1 Text Supplementary file with additional real data analysis results and additional simulation results. (PDF)
