# Supplementary material for: A practical problem with Egger regression in Mendelian randomization
Source: PLoS Genet. 2022 May 4;18(5):e1010166. doi: 10.1371/journal.pgen.1010166 (PMC9109933; doi:10.1371/journal.pgen.1010166)
Supplement: S1 Text — (PDF) [file pgen.1010166.s001.pdf]

Supplementary materials: A practical problem with Egger regression in Mendelian randomization

Contents

A Additional results for real data analysis **2**

B Main simulation results **7**

B.1 Simulation (a): directional pleiotropy 7

B.2 Simulation (a): balanced pleiotropy 15

B.3 Simulation (b): directional pleiotropy 21

B.4 Simulation (b): balanced pleiotropy 30

B.5 Simulation (c): directional pleiotropy 36

B.6 Simulation (c): balanced pleiotropy 45

B.7 Figures for assessing the weak InSIDE assumption in simulations 50

C Additional simulation results under the NOME assumption **52**

D Additional simulations evaluating the impact of SNP coding on violation of NOME assumption **53**

E Simulations with individual-level data **54**

E.1 Simulation (a): directional pleiotropy 57

E.2 Simulation (a): balanced pleiotropy 59

E.3 Simulation (b): directional pleiotropy 60

E.4 Simulation (b): balanced pleiotropy 62

F Additional simulations for PMR-Egger **63**

F.1 Simulation (a): directional pleiotropy 64

F.2 Simulation (b): directional pleiotropy 66

G Simulations with irrelevant IVs **67**

G.1 Simulation (a): directional pleiotropy 69

G.2 Simulation (b): directional pleiotropy 73

H Additional simulations for intercept testing in MR-Egger **76**

H.1 Simulation set-ups 78

H.2 Using the default coding controlled the type-I error of the intercept test when all IVs were valid 79

H.3 Using the default coding was more powerful than using random codings . 79

List of Figures

A Testing the intercept in MR-Egger for 12 risk factors and T2D. 3

B Testing the intercept in MR-Egger for 12 risk factors and CAD. 4

C Testing the intercept in MR-Egger for 12 risk factors and stroke. 5

D Testing the intercept in MR-Egger for 12 risk factors and asthma. 6

E MR-RAPS results under simulation (b) with directional pleiotropy. . . . 21

F MR-RAPS results under Simulation (c) results with directional pleiotropy. 36

G Distributions of $cov(\boldsymbol{\alpha}^*, \boldsymbol{\beta}_X^*)$ under the default and oracle codings. 51

H Simulation (b) results with directional pleiotropy under NOME. 52

I Comparison of the causal estimates from MR-Egger and TSLS-Egger. . . 56

J Simulation (a) results with directional pleiotropy and 10 weak IVs, $\theta = 0$. 69

K Simulation (a) results with directional pleiotropy and 10 weak IVs, $\theta = 0.2$. 70

L Simulation (a) results with directional pleiotropy and 30 weak IVs, $\theta = 0$. 71

M Simulation (a) results with directional pleiotropy and 30 weak IVs, $\theta = 0.2$. 72

N Simulation (b) results with directional pleiotropy and 10 weak IVs, $\theta = 0$. 73

O Simulation (b) results with directional pleiotropy and 10 weak IVs, $\theta = 0.2$. 74

P Simulation (b) results with directional pleiotropy and 30 weak IVs, $\theta = 0$. 75

Q Simulation (b) results with directional pleiotropy and 30 weak IVs, $\theta = 0.2$. 76

R A general causal diagram. 77

S Type-I error of testing the intercept in MR-Egger in the absence of invalid IV. . . . 79
T Empirical power of testing the intercept in MR-Egger in the presence of uncorrelated and/or correlated pleiotropy. . . . 81
U Estimates of the intercept in MR-Egger in the presence of uncorrelated and/or correlated pleiotropy. . . . 82
V Empirical power of testing the intercept in MR-Egger in the presence of correlated pleiotropy. . . . 83
W Estimates of the intercept in MR-Egger in the presence of correlated pleiotropy. 84

List of Tables

A Simulation (a) results with all valid IVs, $n = 50\,000$. . . . 7
B Simulation (a) results with directional pleiotropy, $n = 50\,000$, p_invalid=0.3. 8
C Simulation (a) results with directional pleiotropy, $n = 50\,000$, p_invalid=0.7. 9
D Simulation (a) results with directional pleiotropy, $n = 50\,000$, p_invalid=1. 10
E Simulation (a) results with all valid IVs, $n = 1e + 05$. . . . 11
F Simulation (a) results with directional pleiotropy, $n = 1e{+}05$, p_invalid=0.3. 12
G Simulation (a) results with directional pleiotropy, $n = 1e{+}05$, p_invalid=0.7. 13
H Simulation (a) results with directional pleiotropy, $n = 1e + 05$, p_invalid=1. 14
I Simulation (a) results with balanced pleiotropy, $n = 50\,000$, p_invalid=0.3. 15
J Simulation (a) results with balanced pleiotropy, $n = 50\,000$, p_invalid=0.7. 16
K Simulation (a) results with balanced pleiotropy, $n = 50\,000$, p_invalid=1. 17
L Simulation (a) results with balanced pleiotropy, $n = 1e + 05$, p_invalid=0.3. 18
M Simulation (a) results with balanced pleiotropy, $n = 1e + 05$, p_invalid=0.7. 19
N Simulation (a) results with balanced pleiotropy, $n = 1e + 05$, p_invalid=1. 20
O Simulation (b) results with all valid IVs, $n = 50\,000$. . . . 22
P Simulation (b) results with directional pleiotropy, $n = 50\,000$, p_invalid=0.3. 23
Q Simulation (b) results with directional pleiotropy, $n = 50\,000$, p_invalid=0.7. 24

R Simulation (b) results with directional pleiotropy, $n = 50\,000$, p_invalid=1. 25
S Simulation (b) results with all valid IVs, $n = 1e + 05$. 26
T Simulation (b) results with directional pleiotropy, $n = 1e{+}05$, p_invalid=0.3. 27
U Simulation (b) results with directional pleiotropy, $n = 1e{+}05$, p_invalid=0.7. 28
V Simulation (b) results with directional pleiotropy, $n = 1e + 05$, p_invalid=1. 29
W Simulation (b) results with balanced pleiotropy, $n = 50\,000$, p_invalid=0.3. 30
X Simulation (b) results with balanced pleiotropy, $n = 50\,000$, p_invalid=0.7. 31
Y Simulation (b) results with balanced pleiotropy, $n = 50\,000$, p_invalid=1. 32
Z Simulation (b) results with balanced pleiotropy, $n = 1e + 05$, p_invalid=0.3. 33
AA Simulation (b) results with balanced pleiotropy, $n = 1e + 05$, p_invalid=0.7. 34
AB Simulation (b) results with balanced pleiotropy, $n = 1e + 05$, p_invalid=1. 35
AC Simulation (c) results with all valid IVs, $n = 50\,000$. 37
AD Simulation (c) results with directional pleiotropy, $n = 50\,000$, p_invalid=0.3. 38
AE Simulation (c) results with directional pleiotropy, $n = 50\,000$, p_invalid=0.7. 39
AF Simulation (c) results with directional pleiotropy, $n = 50\,000$, p_invalid=1. 40
AG Simulation (c) results with all valid IVs, $n = 1e + 05$. 41
AH Simulation (c) results with directional pleiotropy, $n = 1e{+}05$, p_invalid=0.3. 42
AI Simulation (c) results with directional pleiotropy, $n = 1e{+}05$, p_invalid=0.7. 43
AJ Simulation (c) results with directional pleiotropy, $n = 1e + 05$, p_invalid=1. 44
AK Simulation (c) results with balanced pleiotropy, $n = 50\,000$, p_invalid=0.3. 45
AL Simulation (c) results with balanced pleiotropy, $n = 50\,000$, p_invalid=0.7. 46
AM Simulation (c) results with balanced pleiotropy, $n = 50\,000$, p_invalid=1. 47
AN Simulation (c) results with balanced pleiotropy, $n = 1e + 05$, p_invalid=0.3. 48
AO Simulation (c) results with balanced pleiotropy, $n = 1e + 05$, p_invalid=0.7. 49
AP Simulation (c) results with balanced pleiotropy, $n = 1e + 05$, p_invalid=1. 50
AQ Simulation results for different IV strengths distributions (i)-(iii). True causal effect $\theta = 1$. 54
AR Individual-level data: Simulation (a) with all valid IVs. 57
AS Individual-level data: Simulation (a) with directional pleiotropy and p_invalid=0.3. 57

AT Individual-level data: Simulation (a) with directional pleiotropy and p_invalid=0.7. 58
AU Individual-level data: Simulation (a) with directional pleiotropy and p_invalid=1. 58
AV Individual-level data: Simulation (a) with balanced pleiotropy and p_invalid=0.3. 59
AW Individual-level data: Simulation (a) with balanced pleiotropy and p_invalid=0.7. 59
AX Individual-level data: Simulation (a) with balanced pleiotropy and p_invalid=1. 60
AY Individual-level data: Simulation (b) with all valid IVs. 60
AZ Individual-level data: Simulation (b) with directional pleiotropy and p_invalid=0.3. 61
BA Individual-level data: Simulation (b) with directional pleiotropy and p_invalid=0.7. 61
BB Individual-level data: Simulation (b) with directional pleiotropy and p_invalid=1. 62
BC Individual-level data: Simulation (b) with balanced pleiotropy and p_invalid=0.3. 62
BD Individual-level data: Simulation (b) with balanced pleiotropy and p_invalid=0.7. 63
BE Individual-level data: Simulation (b) with balanced pleiotropy and p_invalid=1. 63
BF Simulation (a) with equal size directional pleiotropy and p_invalid=0.3. . 64
BG Simulation (a) with equal size directional pleiotropy and p_invalid=0.7. . 65
BH Simulation (a) with equal size directional pleiotropy and p_invalid=1. . . 65
BI Simulation (b) with equal size directional pleiotropy and p_invalid=0.3. . 66
BJ Simulation (b) with equal size directional pleiotropy and p_invalid=0.7. . 66
BK Simulation (b) with equal size directional pleiotropy and p_invalid=1. . . 67

A Additional results for real data analysis

We presented more results of the MR-Egger intercept estimates to see whether the default (i.e. exposure-increasing) coding resulted in the largest intercept estimate (in absolute value) for real data. We randomly flipped the SNP coding 1000 times (including the default coding) and applied MR-Egger. Figs. AA, BA, CA and DA show the empirical distributions of the MR-Egger intercept estimates, and Figs. AB, BB, CB and DB show the corresponding p-values across all unique random coding schemes between 12 risk factors and 4 diseases. We see that using the default coding often yielded more extreme intercept estimates in Egger regression than using other (random) coding schemes, but not necessarily more significant p-values because the standard errors of the intercept estimates were usually larger under the default coding.

A.

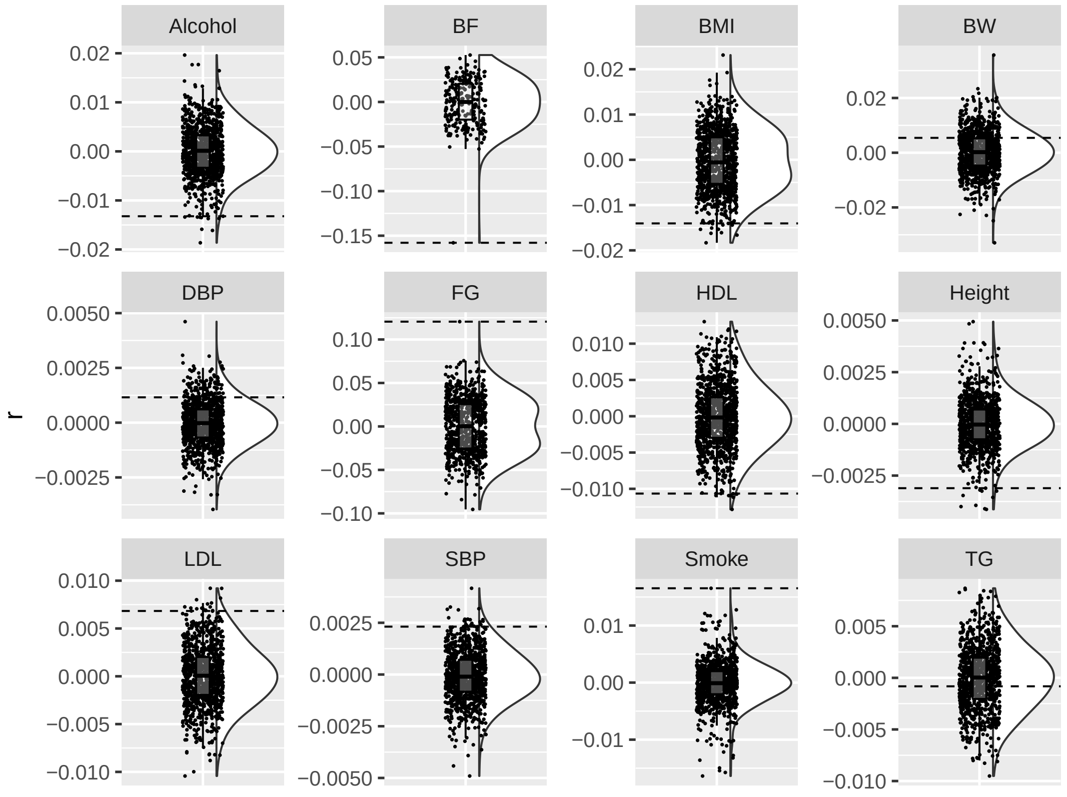

B.

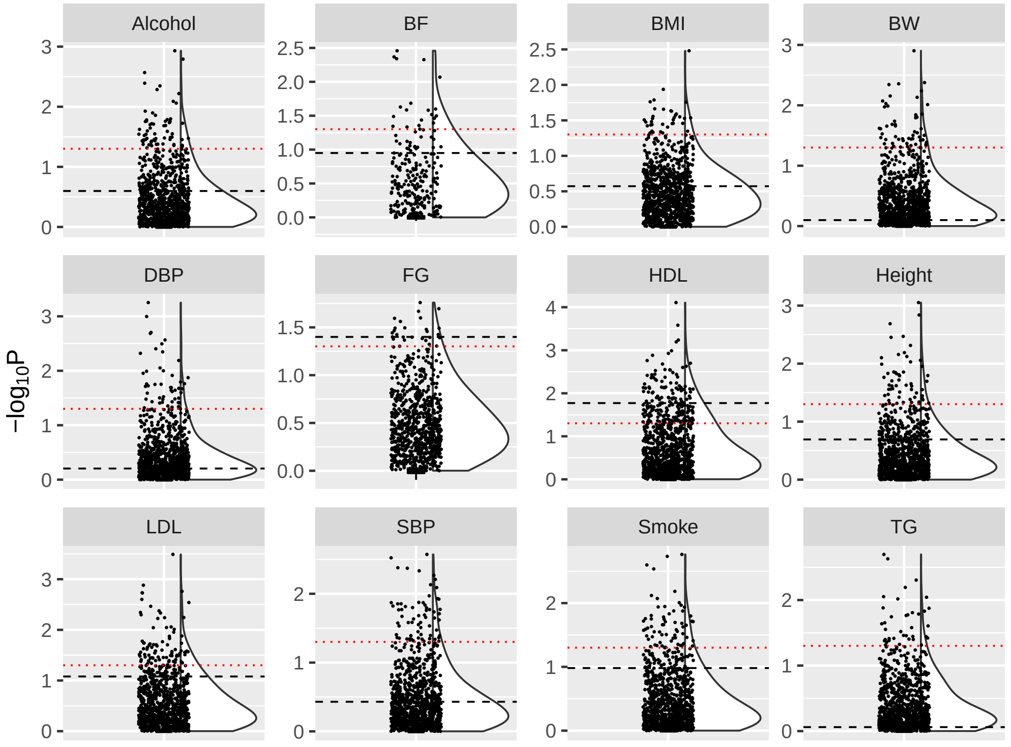

Fig A. Testing the intercept in MR-Egger for 12 risk factors and T2D. Panel A: Estimated intercepts. Panel B: $-\log_{10}$(p-value). The dashed line in each plot corresponds to the result from the *default* coding, and the red dotted line corresponds to a 0.05 p-value threshold.

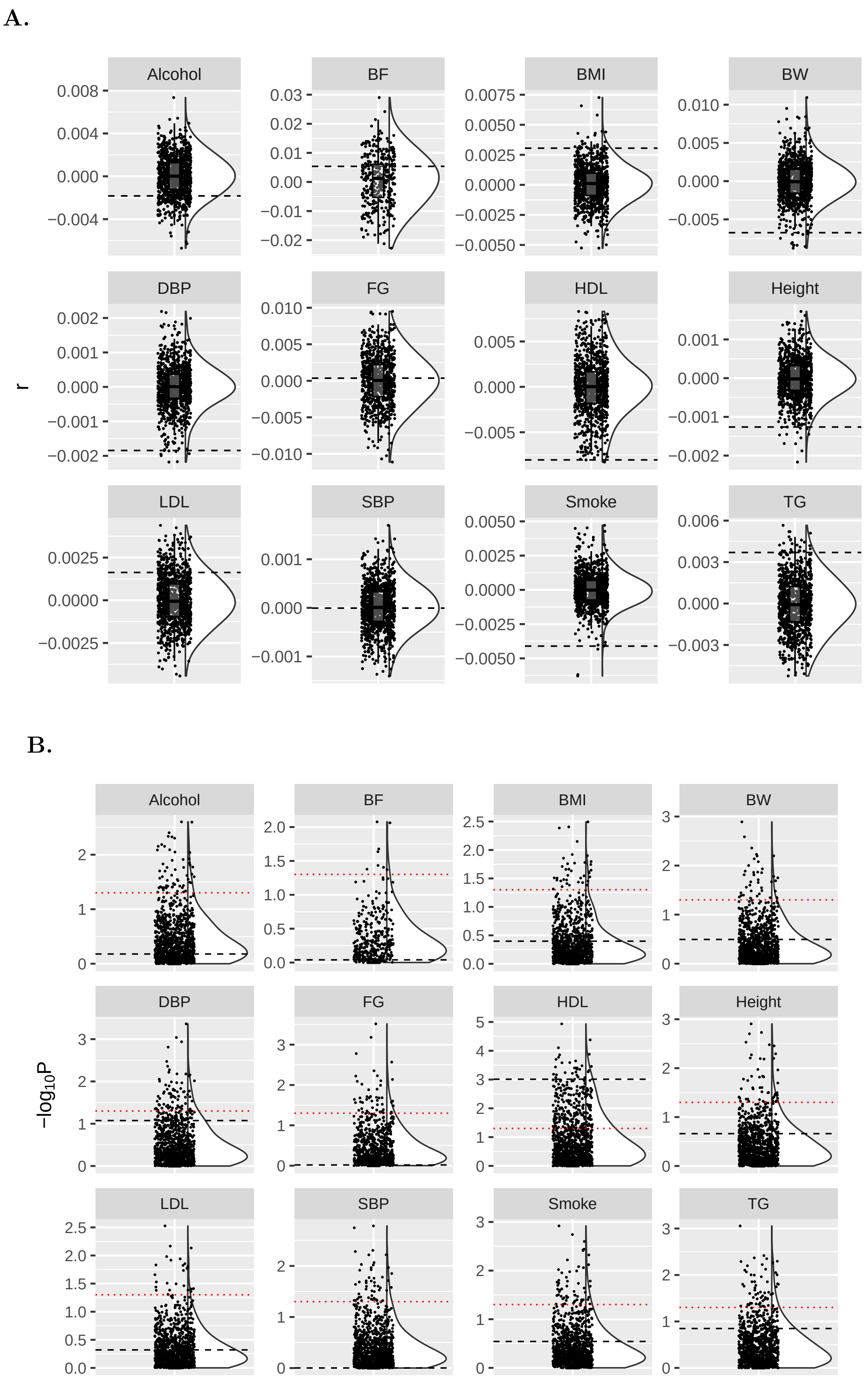

Fig B. Testing the intercept in MR-Egger for 12 risk factors and CAD. Panel A: Estimated intercepts. Panel B: $-\log_{10}$(p-value). The dashed line in each plot corresponds to the result from the *default* coding, and the red dotted line corresponds to a 0.05 p-value threshold.

A.

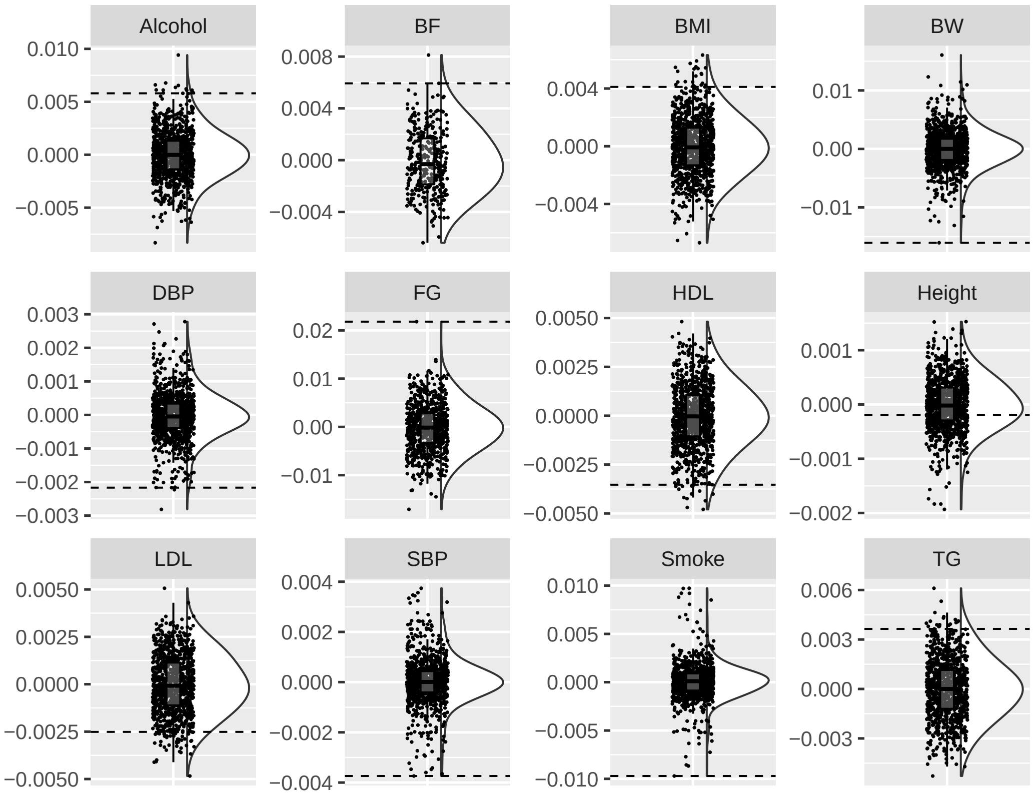

B.

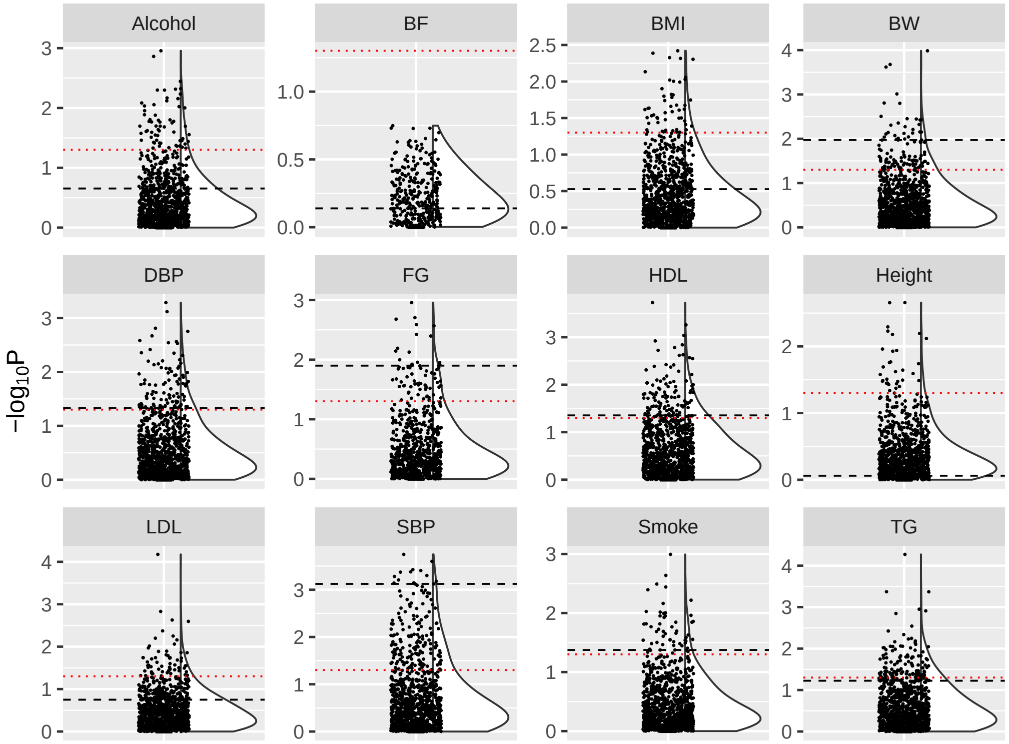

Fig C. Testing the intercept in MR-Egger for 12 risk factors and stroke. Panel A: Estimated intercepts. Panel B: $-\log_{10}$(p-value). The dashed line in each plot corresponds to the result from the *default* coding, and the red dotted line corresponds to a 0.05 p-value threshold.

A.

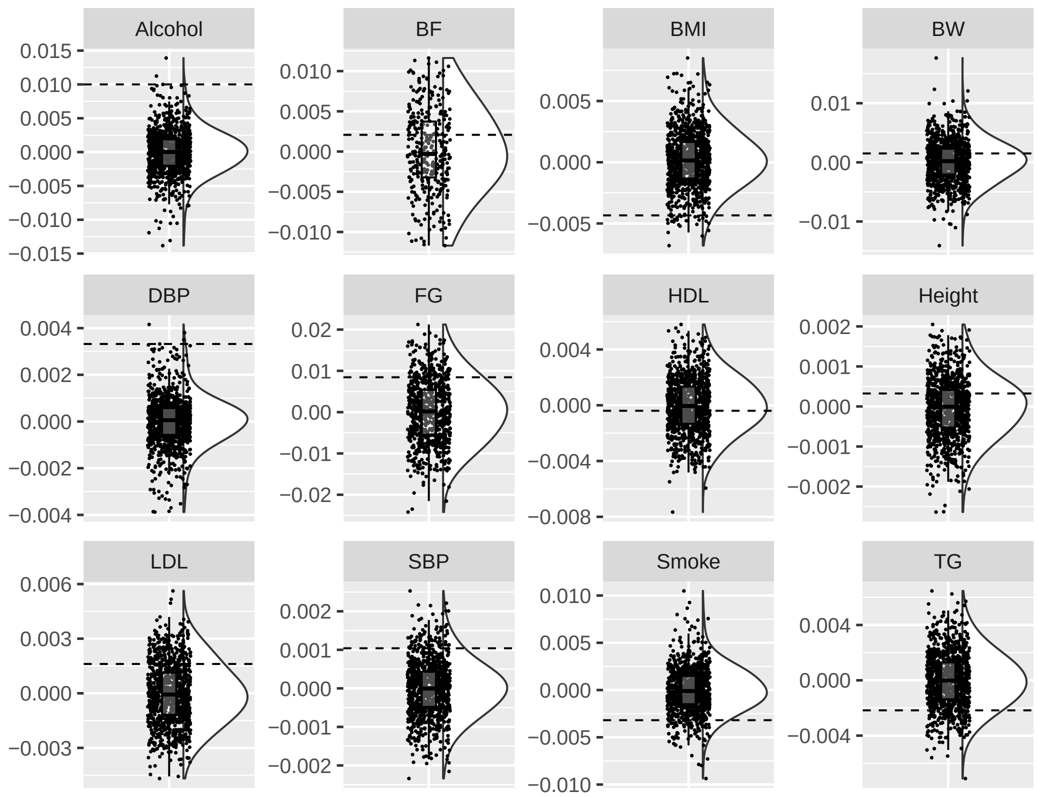

B.

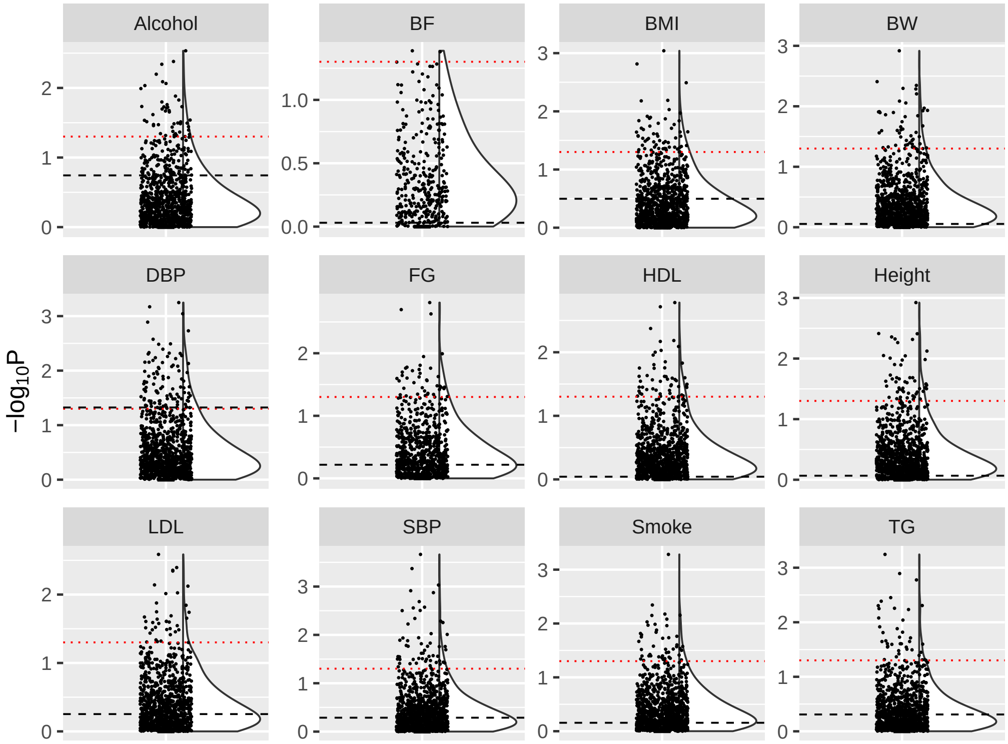

Fig D. Testing the intercept in MR-Egger for 12 risk factors and asthma. Panel A: Estimated intercepts. Panel B: $-\log_{10}$(p-value). The dashed line in each plot corresponds to the result from the *default* coding, and the red dotted line corresponds to a 0.05 p-value threshold.

B Main simulation results

B.1 Simulation (a): directional pleiotropy

Table A. In each cell, from top to bottom are empirical type-I error/power, mean($\hat{\theta}$), SD($\hat{\theta}$), mean(SE($\hat{\theta}$)), coverage rate, MSE, when $n = 50000$, p_invalid=0.

m	θ	oracle	default	random	ivw	radial	mixIE-MA-DP	cML-MA-DP
30	0.0	0.030<br>0.000<br>0.012<br>0.012<br>0.963<br>0.000	0.047<br>0.003<br>0.063<br>0.063<br>0.943<br>0.004	0.028<br>0.001<br>0.016<br>0.016<br>0.966<br>0.000	0.042<br>0.000<br>0.012<br>0.012<br>0.958<br>0.000	0.062<br>0.003<br>0.063<br>0.059<br>0.926<br>0.004	0.032<br>0.000<br>0.012<br>0.013<br>0.968<br>0.000	0.033<br>0.000<br>0.012<br>0.013<br>0.967<br>0.000
	0.2	1.000<br>0.200<br>0.013<br>0.014<br>0.961<br>0.000	0.718<br>0.181<br>0.072<br>0.071<br>0.929<br>0.006	1.000<br>0.199<br>0.018<br>0.018<br>0.962<br>0.000	1.000<br>0.200<br>0.013<br>0.014<br>0.957<br>0.000	0.742<br>0.181<br>0.072<br>0.067<br>0.912<br>0.006	1.000<br>0.199<br>0.013<br>0.015<br>0.966<br>0.000	1.000<br>0.199<br>0.014<br>0.015<br>0.963<br>0.000
100	0.0	0.046<br>0.000<br>0.006<br>0.007<br>0.953<br>0.000	0.034<br>0.000<br>0.032<br>0.032<br>0.959<br>0.001	0.042<br>0.000<br>0.009<br>0.009<br>0.956<br>0.000	0.052<br>0.000<br>0.006<br>0.007<br>0.948<br>0.000	0.046<br>0.000<br>0.032<br>0.032<br>0.952<br>0.001	0.046<br>0.000<br>0.006<br>0.007<br>0.954<br>0.000	0.031<br>0.000<br>0.006<br>0.007<br>0.969<br>0.000
	0.2	1.000<br>0.199<br>0.008<br>0.007<br>0.944<br>0.000	0.997<br>0.171<br>0.036<br>0.037<br>0.869<br>0.002	1.000<br>0.198<br>0.011<br>0.010<br>0.950<br>0.000	1.000<br>0.199<br>0.008<br>0.007<br>0.946<br>0.000	0.997<br>0.171<br>0.036<br>0.036<br>0.864<br>0.002	1.000<br>0.199<br>0.008<br>0.008<br>0.951<br>0.000	1.000<br>0.199<br>0.008<br>0.008<br>0.959<br>0.000

Table B. In each cell, from top to bottom are empirical type-I error/power, mean($\hat{\theta}$), SD($\hat{\theta}$), mean(SE($\hat{\theta}$)), coverage rate, MSE, when $n = 50000$, p_invalid=0.3.

m	θ	oracle	default	random	ivw	radial	mixIE-MA-DP	cML-MA-DP
30	0.0	0.047	0.037	0.049	0.067	0.037	0.036	0.030
		0.000	-0.040	0.002	0.001	-0.040	0.000	0.000
		0.087	0.467	0.128	0.095	0.467	0.016	0.014
		0.087	0.470	0.121	0.092	0.470	0.016	0.016
		0.944	0.956	0.934	0.933	0.958	0.964	0.970
		0.007	0.220	0.016	0.009	0.220	0.000	0.000
	0.2	0.601	0.048	0.427	0.597	0.049	1.000	1.000
		0.199	0.137	0.201	0.200	0.141	0.199	0.197
		0.087	0.467	0.128	0.095	0.467	0.018	0.018
		0.087	0.471	0.122	0.092	0.471	0.019	0.019
		0.943	0.952	0.934	0.933	0.953	0.955	0.954
		0.008	0.222	0.016	0.009	0.221	0.000	0.000
100	0.0	0.046	0.044	0.058	0.037	0.044	0.047	0.026
		0.001	-0.006	-0.002	0.000	-0.006	0.000	0.000
		0.048	0.244	0.077	0.051	0.244	0.009	0.008
		0.047	0.253	0.071	0.051	0.253	0.009	0.010
		0.953	0.953	0.939	0.963	0.952	0.953	0.974
		0.002	0.059	0.006	0.003	0.059	0.000	0.000
	0.2	0.983	0.081	0.791	0.965	0.086	1.000	1.000
		0.200	0.165	0.196	0.199	0.170	0.199	0.199
		0.048	0.243	0.077	0.051	0.243	0.010	0.010
		0.048	0.254	0.072	0.051	0.253	0.011	0.011
		0.952	0.956	0.940	0.962	0.957	0.953	0.961
		0.002	0.060	0.006	0.003	0.060	0.000	0.000

Table C. In each cell, from top to bottom are empirical type-I error/power, mean($\hat{\theta}$), SD($\hat{\theta}$), mean(SE($\hat{\theta}$)), coverage rate, MSE, when $n = 50000$, p_invalid=0.7.

m	θ	oracle	default	random	ivw	radial	mixIE-MA-DP	cML-MA-DP
30	0.0	0.050	0.053	0.054	0.065	0.052	0.032	0.048
		-0.006	-0.057	-0.014	-0.004	-0.058	-0.004	-0.002
		0.118	0.756	0.202	0.148	0.755	0.044	0.026
		0.117	0.729	0.184	0.141	0.729	0.044	0.027
		0.940	0.943	0.933	0.935	0.942	0.968	0.952
		0.014	0.574	0.041	0.022	0.573	0.002	0.001
	0.2	0.350	0.058	0.208	0.309	0.059	0.926	0.924
		0.194	0.123	0.185	0.196	0.133	0.192	0.177
		0.118	0.756	0.202	0.148	0.755	0.051	0.042
		0.117	0.729	0.184	0.141	0.729	0.051	0.041
		0.940	0.942	0.930	0.936	0.941	0.960	0.920
		0.014	0.576	0.041	0.022	0.574	0.003	0.002
100	0.0	0.048	0.038	0.037	0.039	0.037	0.018	0.028
		-0.004	-0.005	-0.005	-0.005	-0.005	0.000	0.000
		0.063	0.372	0.108	0.075	0.372	0.018	0.017
		0.063	0.384	0.106	0.078	0.384	0.021	0.018
		0.946	0.960	0.962	0.961	0.961	0.982	0.972
		0.004	0.138	0.012	0.006	0.138	0.000	0.000
	0.2	0.867	0.064	0.516	0.711	0.066	1.000	1.000
		0.196	0.167	0.194	0.194	0.176	0.198	0.194
		0.063	0.371	0.108	0.075	0.371	0.020	0.022
		0.063	0.385	0.106	0.078	0.384	0.024	0.022
		0.950	0.959	0.963	0.961	0.960	0.975	0.943
		0.004	0.139	0.012	0.006	0.138	0.000	0.001

Table D. In each cell, from top to bottom are empirical type-I error/power, mean($\hat{\theta}$), SD($\hat{\theta}$), mean(SE($\hat{\theta}$)), coverage rate, MSE, when $n = 50000$, p_invalid=1.

m	θ	oracle	default	random	ivw	radial	mixIE-MA-DP	cML-MA-DP
30	0.0	0.057	0.040	0.054	0.073	0.040	0.069	0.222
		-0.006	-0.040	-0.002	-0.003	-0.041	-0.029	-0.004
		0.123	0.864	0.243	0.178	0.864	0.491	0.151
		0.122	0.865	0.223	0.169	0.865	0.578	0.078
		0.937	0.951	0.933	0.927	0.951	0.931	0.778
		0.015	0.748	0.059	0.032	0.748	0.241	0.023
	0.2	0.334	0.041	0.162	0.242	0.041	0.076	0.248
		0.194	0.139	0.197	0.197	0.152	0.140	0.009
		0.123	0.864	0.243	0.178	0.863	0.514	0.159
		0.122	0.865	0.223	0.169	0.865	0.603	0.081
		0.937	0.949	0.932	0.929	0.950	0.939	0.314
		0.015	0.749	0.059	0.032	0.747	0.268	0.062
100	0.0	0.052	0.039	0.034	0.040	0.038	0.023	0.159
		-0.003	-0.019	-0.006	-0.005	-0.019	-0.019	-0.001
		0.067	0.434	0.130	0.088	0.434	0.367	0.114
		0.066	0.460	0.126	0.093	0.460	0.467	0.079
		0.945	0.958	0.962	0.960	0.957	0.977	0.841
		0.004	0.188	0.017	0.008	0.189	0.135	0.013
	0.2	0.840	0.055	0.402	0.569	0.059	0.024	0.152
		0.197	0.153	0.193	0.195	0.164	0.147	0.006
		0.067	0.433	0.130	0.088	0.434	0.382	0.129
		0.066	0.460	0.126	0.093	0.460	0.474	0.084
		0.946	0.961	0.963	0.960	0.961	0.973	0.334
		0.004	0.190	0.017	0.008	0.189	0.148	0.055

Table E. In each cell, from top to bottom are empirical type-I error/power, mean($\hat{\theta}$), SD($\hat{\theta}$), mean(SE($\hat{\theta}$)), coverage rate, MSE, when $n = 1e + 05$, p_invalid=0.

m	θ	oracle	default	random	ivw	radial	mixIE-MA-DP	cML-MA-DP
30	0.0	0.037	0.035	0.036	0.045	0.047	0.037	0.033
		0.000	0.000	0.000	0.000	0.000	0.000	0.000
		0.009	0.044	0.011	0.008	0.044	0.009	0.008
		0.009	0.046	0.011	0.009	0.043	0.009	0.009
		0.953	0.955	0.958	0.955	0.942	0.963	0.967
		0.000	0.002	0.000	0.000	0.002	0.000	0.000
	0.2	1.000	0.947	1.000	1.000	0.951	1.000	1.000
		0.200	0.189	0.199	0.200	0.189	0.199	0.199
		0.010	0.049	0.012	0.010	0.049	0.010	0.010
		0.010	0.051	0.013	0.010	0.049	0.010	0.010
		0.948	0.951	0.960	0.950	0.935	0.957	0.962
		0.000	0.003	0.000	0.000	0.003	0.000	0.000
100	0.0	0.042	0.045	0.045	0.045	0.052	0.049	0.032
		0.000	-0.001	0.000	0.000	-0.001	0.000	0.000
		0.005	0.023	0.007	0.005	0.023	0.005	0.005
		0.005	0.024	0.006	0.005	0.023	0.005	0.005
		0.954	0.952	0.951	0.955	0.945	0.951	0.968
		0.000	0.001	0.000	0.000	0.001	0.000	0.000
	0.2	1.000	1.000	1.000	1.000	1.000	1.000	1.000
		0.200	0.183	0.199	0.200	0.183	0.200	0.200
		0.005	0.027	0.008	0.005	0.027	0.005	0.005
		0.005	0.027	0.007	0.005	0.026	0.005	0.006
		0.951	0.902	0.942	0.949	0.896	0.946	0.966
		0.000	0.001	0.000	0.000	0.001	0.000	0.000

Table F. In each cell, from top to bottom are empirical type-I error/power, mean($\hat{\theta}$), SD($\hat{\theta}$), mean(SE($\hat{\theta}$)), coverage rate, MSE, when $n = 1e + 05$, p_invalid=0.3.

m	θ	oracle	default	random	ivw	radial	mixIE-MA-DP	cML-MA-DP
30	0.0	0.057<br>0.001<br>0.090<br>0.086<br>0.931<br>0.008	0.040<br>-0.007<br>0.492<br>0.479<br>0.945<br>0.242	0.063<br>0.002<br>0.143<br>0.122<br>0.923<br>0.020	0.071<br>0.000<br>0.095<br>0.091<br>0.929<br>0.009	0.041<br>-0.008<br>0.491<br>0.479<br>0.948<br>0.241	0.040<br>0.001<br>0.011<br>0.011<br>0.960<br>0.000	0.036<br>0.001<br>0.010<br>0.011<br>0.964<br>0.000
	0.2	0.615<br>0.201<br>0.090<br>0.086<br>0.926<br>0.008	0.062<br>0.181<br>0.493<br>0.479<br>0.941<br>0.243	0.420<br>0.202<br>0.143<br>0.122<br>0.923<br>0.020	0.608<br>0.200<br>0.095<br>0.091<br>0.930<br>0.009	0.064<br>0.185<br>0.492<br>0.479<br>0.944<br>0.242	1.000<br>0.201<br>0.012<br>0.013<br>0.960<br>0.000	1.000<br>0.200<br>0.012<br>0.013<br>0.961<br>0.000
100	0.0	0.042<br>0.000<br>0.047<br>0.047<br>0.954<br>0.002	0.055<br>0.007<br>0.258<br>0.258<br>0.939<br>0.067	0.052<br>0.004<br>0.075<br>0.069<br>0.945<br>0.006	0.050<br>0.001<br>0.051<br>0.050<br>0.950<br>0.003	0.056<br>0.007<br>0.258<br>0.258<br>0.941<br>0.067	0.046<br>0.000<br>0.006<br>0.006<br>0.954<br>0.000	0.032<br>0.000<br>0.006<br>0.007<br>0.968<br>0.000
	0.2	0.988<br>0.200<br>0.047<br>0.047<br>0.954<br>0.002	0.108<br>0.192<br>0.258<br>0.258<br>0.940<br>0.067	0.835<br>0.203<br>0.075<br>0.069<br>0.947<br>0.006	0.976<br>0.201<br>0.051<br>0.050<br>0.950<br>0.003	0.111<br>0.196<br>0.258<br>0.258<br>0.939<br>0.067	1.000<br>0.200<br>0.007<br>0.007<br>0.956<br>0.000	1.000<br>0.199<br>0.007<br>0.008<br>0.968<br>0.000

Table G. In each cell, from top to bottom are empirical type-I error/power, mean($\hat{\theta}$), SD($\hat{\theta}$), mean(SE($\hat{\theta}$)), coverage rate, MSE, when $n = 1e + 05$, p_invalid=0.7.

m	θ	oracle	default	random	ivw	radial	mixIE-MA-DP	cML-MA-DP
30	0.0	0.058	0.055	0.046	0.056	0.055	0.037	0.051
		-0.001	-0.001	0.001	-0.001	-0.002	0.001	0.000
		0.119	0.764	0.195	0.141	0.764	0.021	0.018
		0.116	0.737	0.185	0.140	0.737	0.026	0.018
		0.931	0.930	0.945	0.944	0.930	0.963	0.949
		0.014	0.583	0.038	0.020	0.582	0.000	0.000
	0.2	0.393	0.067	0.201	0.304	0.067	0.983	0.974
		0.199	0.187	0.201	0.199	0.197	0.200	0.188
		0.119	0.763	0.195	0.141	0.762	0.025	0.030
		0.116	0.737	0.185	0.140	0.737	0.030	0.029
		0.932	0.931	0.946	0.944	0.935	0.957	0.922
		0.014	0.582	0.038	0.020	0.580	0.001	0.001
100	0.0	0.057	0.053	0.064	0.063	0.052	0.030	0.048
		0.003	0.006	0.008	0.005	0.006	0.000	0.000
		0.064	0.401	0.122	0.079	0.401	0.012	0.012
		0.063	0.394	0.107	0.077	0.394	0.014	0.012
		0.941	0.945	0.931	0.937	0.943	0.970	0.952
		0.004	0.160	0.015	0.006	0.161	0.000	0.000
	0.2	0.883	0.077	0.563	0.760	0.080	1.000	1.000
		0.203	0.191	0.207	0.205	0.200	0.199	0.196
		0.064	0.400	0.122	0.079	0.401	0.014	0.014
		0.063	0.394	0.107	0.077	0.394	0.016	0.015
		0.939	0.942	0.933	0.938	0.940	0.966	0.942
		0.004	0.160	0.015	0.006	0.160	0.000	0.000

Table H. In each cell, from top to bottom are empirical type-I error/power, mean($\hat{\theta}$), SD($\hat{\theta}$), mean(SE($\hat{\theta}$)), coverage rate, MSE, when $n = 1e + 05$, p_invalid=1.

m	θ	oracle	default	random	ivw	radial	mixIE-MA-DP	cML-MA-DP
30	0.0	0.058<br>0.001<br>0.125<br>0.121<br>0.937<br>0.016	0.047<br>-0.014<br>0.907<br>0.889<br>0.941<br>0.823	0.051<br>-0.009<br>0.235<br>0.223<br>0.934<br>0.055	0.056<br>0.000<br>0.171<br>0.169<br>0.944<br>0.029	0.048<br>-0.015<br>0.907<br>0.889<br>0.941<br>0.822	0.149<br>-0.009<br>0.463<br>0.531<br>0.851<br>0.214	0.306<br>0.003<br>0.144<br>0.057<br>0.694<br>0.021
	0.2	0.369<br>0.201<br>0.125<br>0.121<br>0.938<br>0.016	0.052<br>0.176<br>0.907<br>0.889<br>0.943<br>0.822	0.167<br>0.191<br>0.235<br>0.222<br>0.934<br>0.055	0.235<br>0.200<br>0.171<br>0.169<br>0.945<br>0.029	0.051<br>0.190<br>0.906<br>0.889<br>0.944<br>0.821	0.128<br>0.119<br>0.456<br>0.532<br>0.855<br>0.214	0.260<br>0.005<br>0.149<br>0.062<br>0.189<br>0.060
100	0.0	0.054<br>0.002<br>0.067<br>0.066<br>0.945<br>0.005	0.055<br>-0.006<br>0.474<br>0.472<br>0.939<br>0.225	0.057<br>0.004<br>0.134<br>0.125<br>0.939<br>0.018	0.068<br>0.006<br>0.097<br>0.092<br>0.932<br>0.009	0.055<br>-0.005<br>0.475<br>0.472<br>0.939<br>0.225	0.093<br>-0.005<br>0.338<br>0.433<br>0.907<br>0.114	0.196<br>0.004<br>0.111<br>0.058<br>0.804<br>0.012
	0.2	0.862<br>0.202<br>0.067<br>0.066<br>0.945<br>0.005	0.072<br>0.179<br>0.474<br>0.472<br>0.942<br>0.225	0.438<br>0.204<br>0.134<br>0.125<br>0.941<br>0.018	0.611<br>0.206<br>0.097<br>0.092<br>0.932<br>0.009	0.072<br>0.192<br>0.474<br>0.472<br>0.941<br>0.224	0.072<br>0.111<br>0.329<br>0.438<br>0.924<br>0.116	0.195<br>0.008<br>0.117<br>0.062<br>0.212<br>0.050

B.2 Simulation (a): balanced pleiotropy

Table I. In each cell, from top to bottom are empirical type-I error/power, mean($\hat{\theta}$), SD($\hat{\theta}$), mean(SE($\hat{\theta}$)), coverage rate, MSE, when $n = 50000$, p_invalid=0.3.

m	θ	oracle	default	random	ivw	radial	mixIE-MA-DP	cML-MA-DP
30	0.0	0.055	0.051	0.060	0.065	0.051	0.048	0.034
		-0.001	-0.027	0.000	0.000	-0.027	0.000	0.000
		0.068	0.343	0.093	0.067	0.343	0.016	0.015
		0.066	0.332	0.086	0.065	0.332	0.017	0.016
		0.934	0.942	0.930	0.935	0.943	0.952	0.966
		0.005	0.118	0.009	0.005	0.118	0.000	0.000
	0.2	0.807	0.074	0.665	0.838	0.075	1.000	1.000
		0.199	0.149	0.199	0.199	0.152	0.199	0.197
		0.068	0.344	0.094	0.067	0.344	0.018	0.018
		0.066	0.334	0.086	0.065	0.334	0.020	0.020
		0.936	0.931	0.933	0.936	0.930	0.953	0.956
		0.005	0.121	0.009	0.005	0.120	0.000	0.000
100	0.0	0.043	0.053	0.052	0.048	0.053	0.051	0.034
		-0.001	-0.004	-0.002	-0.001	-0.004	0.000	0.000
		0.037	0.180	0.054	0.037	0.181	0.009	0.009
		0.036	0.180	0.051	0.036	0.180	0.009	0.009
		0.952	0.945	0.946	0.952	0.946	0.949	0.966
		0.001	0.033	0.003	0.001	0.033	0.000	0.000
	0.2	1.000	0.171	0.905	1.000	0.174	1.000	1.000
		0.198	0.167	0.195	0.198	0.170	0.199	0.199
		0.037	0.181	0.055	0.037	0.181	0.011	0.011
		0.037	0.181	0.051	0.036	0.181	0.011	0.011
		0.949	0.937	0.943	0.947	0.936	0.937	0.953
		0.001	0.034	0.003	0.001	0.034	0.000	0.000

Table J. In each cell, from top to bottom are empirical type-I error/power, mean($\hat{\theta}$), SD($\hat{\theta}$), mean(SE($\hat{\theta}$)), coverage rate, MSE, when $n = 50000$, p_invalid=0.7.

m	θ	oracle	default	random	ivw	radial	mixIE-MA-DP	cML-MA-DP
30	0.0	0.062	0.058	0.058	0.068	0.058	0.043	0.062
		-0.006	-0.030	-0.013	-0.005	-0.031	-0.004	-0.002
		0.104	0.555	0.142	0.102	0.554	0.043	0.028
		0.102	0.515	0.130	0.100	0.515	0.043	0.027
		0.934	0.935	0.935	0.932	0.935	0.957	0.938
		0.011	0.308	0.020	0.010	0.308	0.002	0.001
	0.2	0.442	0.074	0.331	0.489	0.075	0.927	0.900
		0.194	0.149	0.186	0.195	0.154	0.194	0.173
		0.104	0.556	0.142	0.102	0.555	0.045	0.047
		0.102	0.516	0.131	0.100	0.516	0.050	0.042
		0.932	0.935	0.932	0.932	0.935	0.954	0.866
		0.011	0.311	0.020	0.010	0.310	0.002	0.003
100	0.0	0.048	0.055	0.051	0.046	0.054	0.025	0.041
		-0.004	0.003	-0.004	-0.004	0.003	0.000	0.001
		0.055	0.277	0.079	0.055	0.277	0.019	0.018
		0.055	0.272	0.075	0.055	0.272	0.021	0.018
		0.952	0.945	0.947	0.954	0.945	0.975	0.959
		0.003	0.076	0.006	0.003	0.077	0.000	0.000
	0.2	0.947	0.098	0.776	0.950	0.101	1.000	1.000
		0.195	0.174	0.194	0.195	0.179	0.199	0.194
		0.055	0.277	0.080	0.055	0.277	0.021	0.023
		0.055	0.273	0.075	0.055	0.273	0.024	0.023
		0.952	0.941	0.946	0.956	0.940	0.974	0.943
		0.003	0.077	0.006	0.003	0.077	0.000	0.001

Table K. In each cell, from top to bottom are empirical type-I error/power, mean($\hat{\theta}$), SD($\hat{\theta}$), mean(SE($\hat{\theta}$)), coverage rate, MSE, when $n = 50000$, p_invalid=1.

m	θ	oracle	default	random	ivw	radial	mixIE-MA-DP	cML-MA-DP
30	0.0	0.055	0.045	0.050	0.062	0.046	0.060	0.173
		-0.006	-0.017	-0.005	-0.005	-0.018	0.007	0.000
		0.123	0.627	0.169	0.120	0.627	0.332	0.101
		0.122	0.612	0.158	0.119	0.612	0.416	0.067
		0.937	0.944	0.936	0.938	0.946	0.940	0.827
		0.015	0.393	0.028	0.015	0.393	0.110	0.010
	0.2	0.332	0.056	0.249	0.375	0.057	0.114	0.198
		0.194	0.161	0.194	0.195	0.168	0.194	0.041
		0.123	0.627	0.169	0.120	0.626	0.329	0.123
		0.122	0.612	0.158	0.120	0.612	0.420	0.072
		0.938	0.945	0.935	0.937	0.946	0.945	0.334
		0.015	0.394	0.029	0.015	0.393	0.108	0.040
100	0.0	0.054	0.052	0.046	0.055	0.054	0.012	0.121
		-0.003	0.001	-0.001	-0.003	0.001	0.001	0.001
		0.067	0.325	0.096	0.066	0.326	0.246	0.083
		0.066	0.325	0.089	0.066	0.325	0.322	0.063
		0.943	0.947	0.951	0.945	0.944	0.988	0.879
		0.004	0.106	0.009	0.004	0.106	0.060	0.007
	0.2	0.841	0.090	0.656	0.846	0.091	0.037	0.214
		0.197	0.172	0.197	0.196	0.179	0.177	0.062
		0.067	0.326	0.096	0.066	0.326	0.250	0.106
		0.066	0.325	0.089	0.066	0.325	0.328	0.071
		0.943	0.943	0.953	0.947	0.942	0.987	0.436
		0.004	0.107	0.009	0.004	0.106	0.063	0.030

Table L. In each cell, from top to bottom are empirical type-I error/power, mean($\hat{\theta}$), SD($\hat{\theta}$), mean(SE($\hat{\theta}$)), coverage rate, MSE, when $n = 1e + 05$, p_invalid=0.3.

m	θ	oracle	default	random	ivw	radial	mixIE-MA-DP	cML-MA-DP
30	0.0	0.045<br>0.001<br>0.066<br>0.065<br>0.940<br>0.004	0.046<br>0.004<br>0.338<br>0.335<br>0.951<br>0.114	0.055<br>0.002<br>0.096<br>0.085<br>0.931<br>0.009	0.068<br>0.001<br>0.066<br>0.064<br>0.932<br>0.004	0.046<br>0.004<br>0.338<br>0.336<br>0.950<br>0.114	0.044<br>0.001<br>0.011<br>0.012<br>0.956<br>0.000	0.035<br>0.001<br>0.010<br>0.011<br>0.965<br>0.000
	0.2	0.812<br>0.201<br>0.067<br>0.065<br>0.942<br>0.004	0.074<br>0.192<br>0.339<br>0.336<br>0.948<br>0.115	0.653<br>0.201<br>0.096<br>0.086<br>0.931<br>0.009	0.844<br>0.201<br>0.066<br>0.064<br>0.937<br>0.004	0.075<br>0.194<br>0.339<br>0.336<br>0.948<br>0.115	1.000<br>0.200<br>0.013<br>0.013<br>0.959<br>0.000	1.000<br>0.199<br>0.013<br>0.013<br>0.963<br>0.000
100	0.0	0.047<br>0.001<br>0.036<br>0.036<br>0.948<br>0.001	0.049<br>0.002<br>0.184<br>0.183<br>0.947<br>0.034	0.044<br>0.003<br>0.054<br>0.049<br>0.950<br>0.003	0.054<br>0.001<br>0.035<br>0.036<br>0.946<br>0.001	0.050<br>0.002<br>0.184<br>0.183<br>0.948<br>0.034	0.053<br>0.000<br>0.006<br>0.006<br>0.947<br>0.000	0.037<br>0.000<br>0.006<br>0.006<br>0.963<br>0.000
	0.2	0.999<br>0.201<br>0.036<br>0.036<br>0.951<br>0.001	0.180<br>0.187<br>0.184<br>0.183<br>0.948<br>0.034	0.932<br>0.202<br>0.054<br>0.049<br>0.956<br>0.003	0.999<br>0.201<br>0.035<br>0.036<br>0.949<br>0.001	0.182<br>0.189<br>0.184<br>0.183<br>0.949<br>0.034	1.000<br>0.200<br>0.007<br>0.007<br>0.943<br>0.000	1.000<br>0.199<br>0.007<br>0.008<br>0.954<br>0.000

Table M. In each cell, from top to bottom are empirical type-I error/power, mean($\hat{\theta}$), SD($\hat{\theta}$), mean(SE($\hat{\theta}$)), coverage rate, MSE, when $n = 1e + 05$, p_invalid=0.7.

m	θ	oracle	default	random	ivw	radial	mixIE-MA-DP	cML-MA-DP
30	0.0	0.051	0.058	0.043	0.067	0.056	0.046	0.056
		-0.001	0.013	0.002	0.000	0.013	-0.001	-0.001
		0.103	0.530	0.138	0.101	0.531	0.023	0.019
		0.101	0.520	0.130	0.099	0.520	0.027	0.018
		0.934	0.935	0.947	0.933	0.934	0.954	0.944
		0.011	0.281	0.019	0.010	0.281	0.001	0.000
	0.2	0.480	0.065	0.374	0.527	0.067	0.974	0.959
		0.199	0.201	0.202	0.200	0.207	0.196	0.182
		0.103	0.530	0.139	0.101	0.530	0.048	0.037
		0.101	0.520	0.130	0.099	0.520	0.033	0.032
		0.930	0.936	0.947	0.931	0.935	0.963	0.909
		0.011	0.281	0.019	0.010	0.281	0.002	0.002
100	0.0	0.046	0.052	0.050	0.053	0.053	0.024	0.038
		0.002	0.003	0.004	0.002	0.003	-0.001	0.000
		0.056	0.277	0.086	0.056	0.277	0.012	0.012
		0.055	0.280	0.076	0.055	0.280	0.014	0.012
		0.950	0.944	0.947	0.947	0.945	0.976	0.962
		0.003	0.077	0.007	0.003	0.077	0.000	0.000
	0.2	0.939	0.102	0.783	0.947	0.103	1.000	1.000
		0.202	0.188	0.204	0.202	0.193	0.199	0.195
		0.056	0.277	0.086	0.056	0.277	0.014	0.015
		0.055	0.280	0.076	0.055	0.280	0.016	0.015
		0.949	0.949	0.948	0.948	0.948	0.971	0.936
		0.003	0.077	0.007	0.003	0.077	0.000	0.000

Table N. In each cell, from top to bottom are empirical type-I error/power, mean($\hat{\theta}$), SD($\hat{\theta}$), mean(SE($\hat{\theta}$)), coverage rate, MSE, when $n = 1e + 05$, p_invalid=1.

m	θ	oracle	default	random	ivw	radial	mixIE-MA-DP	cML-MA-DP
30	0.0	0.060	0.054	0.056	0.062	0.054	0.109	0.209
		0.001	0.010	-0.007	0.001	0.010	-0.001	-0.006
		0.125	0.640	0.168	0.123	0.640	0.288	0.093
		0.121	0.627	0.157	0.119	0.627	0.351	0.051
		0.938	0.937	0.932	0.938	0.937	0.891	0.791
		0.016	0.410	0.028	0.015	0.410	0.083	0.009
	0.2	0.366	0.062	0.261	0.402	0.063	0.132	0.235
		0.201	0.199	0.193	0.201	0.207	0.169	0.030
		0.125	0.639	0.169	0.123	0.639	0.306	0.108
		0.121	0.627	0.157	0.119	0.627	0.358	0.054
		0.939	0.938	0.932	0.938	0.937	0.899	0.209
		0.016	0.408	0.028	0.015	0.408	0.094	0.040
100	0.0	0.054	0.050	0.052	0.056	0.051	0.055	0.150
		0.002	-0.005	0.001	0.002	-0.005	0.004	0.000
		0.067	0.332	0.093	0.066	0.332	0.213	0.074
		0.066	0.334	0.088	0.065	0.334	0.283	0.050
		0.945	0.948	0.947	0.944	0.946	0.945	0.850
		0.004	0.110	0.009	0.004	0.110	0.045	0.006
	0.2	0.865	0.085	0.661	0.875	0.093	0.071	0.182
		0.202	0.180	0.201	0.202	0.187	0.171	0.036
		0.067	0.332	0.093	0.066	0.332	0.226	0.084
		0.066	0.335	0.089	0.065	0.335	0.297	0.054
		0.945	0.947	0.948	0.945	0.947	0.955	0.233
		0.004	0.110	0.009	0.004	0.110	0.052	0.034

B.3 Simulation (b): directional pleiotropy

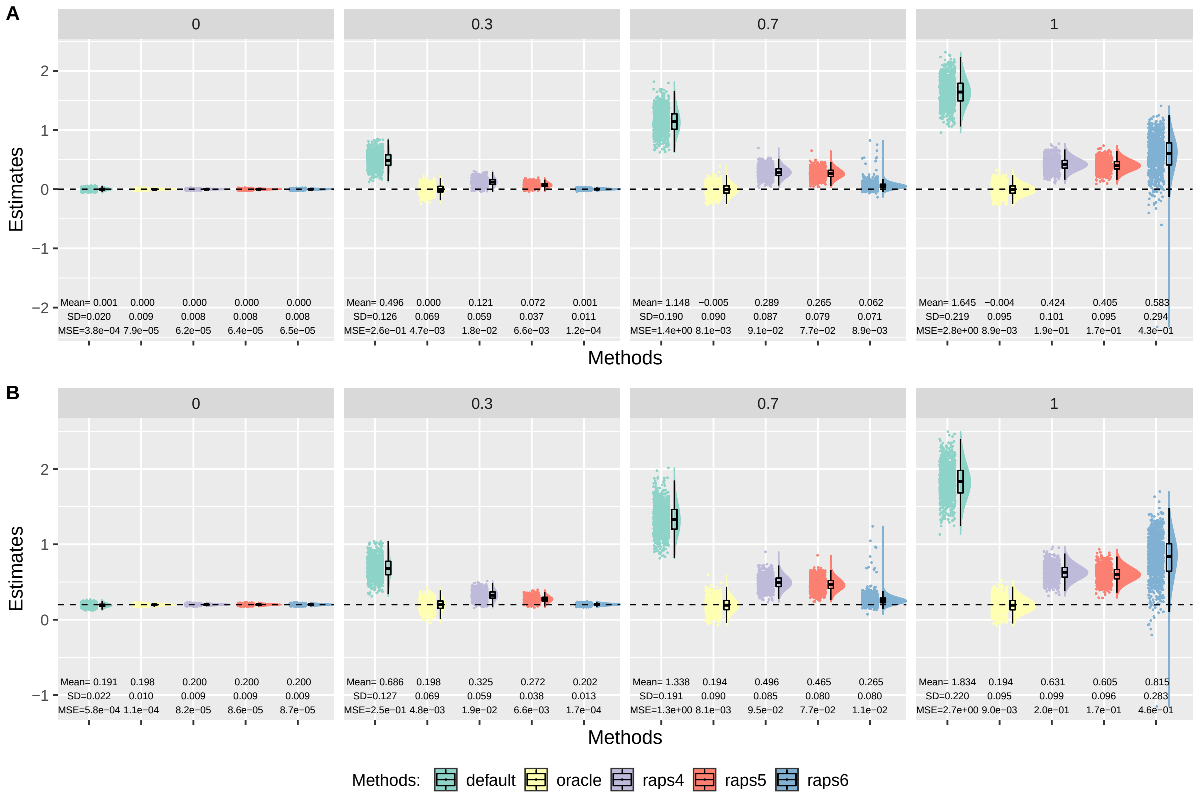

Fig E. Simulation (b) results with directional pleiotropy, $n = 50\,000, m = 100$. Empirical distributions of the estimates of the causal effect θ by the methods. raps4, raps5, raps6 correspond to the three versions of MR-RAPS with an over-dispersed model and with the L2, Huber and Tukey loss functions respectively. Each column corresponds to 0%, 30%, 70% or 100% invalid IVs. A: $\theta = 0$. B: $\theta = 0.2$.

Table O. In each cell, from top to bottom are empirical type-I error/power, mean($\hat{\theta}$), SD($\hat{\theta}$), mean(SE($\hat{\theta}$)), coverage rate, MSE, when $n = 50000$, p_invalid=0.

m	θ	oracle	default	random	ivw	radial	mixIE-MA-DP	cML-MA-DP
30	0.0	0.031<br>0.001<br>0.017<br>0.017<br>0.959<br>0.000	0.023<br>-0.001<br>0.035<br>0.039<br>0.964<br>0.001	0.031<br>0.001<br>0.018<br>0.019<br>0.962<br>0.000	0.033<br>0.001<br>0.014<br>0.015<br>0.967<br>0.000	0.042<br>-0.001<br>0.035<br>0.036<br>0.947<br>0.001	0.031<br>0.001<br>0.014<br>0.015<br>0.969<br>0.000	0.028<br>0.001<br>0.014<br>0.016<br>0.972<br>0.000
	0.2	1.000<br>0.199<br>0.019<br>0.020<br>0.961<br>0.000	0.991<br>0.191<br>0.041<br>0.043<br>0.960<br>0.002	1.000<br>0.199<br>0.021<br>0.021<br>0.953<br>0.000	1.000<br>0.199<br>0.016<br>0.017<br>0.961<br>0.000	0.993<br>0.191<br>0.041<br>0.041<br>0.946<br>0.002	1.000<br>0.199<br>0.016<br>0.017<br>0.964<br>0.000	1.000<br>0.199<br>0.017<br>0.018<br>0.963<br>0.000
100	0.0	0.048<br>0.000<br>0.009<br>0.009<br>0.951<br>0.000	0.049<br>0.001<br>0.020<br>0.020<br>0.948<br>0.000	0.043<br>0.000<br>0.010<br>0.010<br>0.955<br>0.000	0.049<br>0.000<br>0.008<br>0.008<br>0.951<br>0.000	0.054<br>0.001<br>0.020<br>0.020<br>0.944<br>0.000	0.047<br>0.000<br>0.008<br>0.008<br>0.953<br>0.000	0.020<br>0.000<br>0.008<br>0.009<br>0.980<br>0.000
	0.2	1.000<br>0.198<br>0.010<br>0.010<br>0.945<br>0.000	1.000<br>0.191<br>0.022<br>0.023<br>0.933<br>0.001	1.000<br>0.198<br>0.012<br>0.012<br>0.950<br>0.000	1.000<br>0.198<br>0.009<br>0.009<br>0.938<br>0.000	1.000<br>0.191<br>0.022<br>0.022<br>0.928<br>0.001	1.000<br>0.198<br>0.009<br>0.009<br>0.939<br>0.000	1.000<br>0.199<br>0.009<br>0.010<br>0.963<br>0.000
500	0.0	0.051<br>0.000<br>0.004<br>0.004<br>0.949<br>0.000	0.049<br>0.000<br>0.008<br>0.009<br>0.951<br>0.000	0.047<br>0.000<br>0.004<br>0.004<br>0.953<br>0.000	0.052<br>0.000<br>0.003<br>0.004<br>0.948<br>0.000	0.052<br>0.000<br>0.008<br>0.008<br>0.948<br>0.000	0.052<br>0.000<br>0.003<br>0.004<br>0.948<br>0.000	0.029<br>0.000<br>0.004<br>0.004<br>0.971<br>0.000
	0.2	1.000<br>0.196<br>0.005<br>0.005<br>0.840<br>0.000	1.000<br>0.181<br>0.010<br>0.010<br>0.549<br>0.000	1.000<br>0.194<br>0.006<br>0.005<br>0.803<br>0.000	1.000<br>0.197<br>0.004<br>0.004<br>0.858<br>0.000	1.000<br>0.181<br>0.010<br>0.010<br>0.549<br>0.000	1.000<br>0.197<br>0.004<br>0.006<br>0.862<br>0.000	1.000<br>0.198<br>0.004<br>0.005<br>0.946<br>0.000

Table P. In each cell, from top to bottom are empirical type-I error/power, mean($\hat{\theta}$), SD($\hat{\theta}$), mean(SE($\hat{\theta}$)), coverage rate, MSE, when $n = 50000$, p_invalid=0.3.

m	θ	oracle	default	random	ivw	radial	mixIE-MA-DP	cML-MA-DP
30	0.0	0.050	0.391	0.169	0.198	0.391	0.054	0.031
		-0.002	0.491	0.157	0.113	0.492	0.004	0.001
		0.122	0.235	0.137	0.104	0.235	0.020	0.019
		0.122	0.275	0.137	0.111	0.275	0.021	0.020
		0.944	0.569	0.813	0.802	0.567	0.946	0.969
		0.015	0.296	0.043	0.024	0.297	0.000	0.000
	0.2	0.360	0.716	0.741	0.810	0.721	1.000	1.000
		0.197	0.682	0.355	0.312	0.683	0.204	0.199
		0.123	0.237	0.137	0.105	0.236	0.023	0.023
		0.122	0.276	0.138	0.111	0.276	0.023	0.024
		0.944	0.594	0.817	0.808	0.589	0.948	0.956
		0.015	0.288	0.043	0.024	0.289	0.001	0.001
100	0.0	0.057	0.957	0.550	0.485	0.959	0.063	0.033
		0.000	0.496	0.168	0.117	0.497	0.003	0.001
		0.069	0.126	0.101	0.057	0.126	0.011	0.011
		0.066	0.148	0.078	0.061	0.148	0.011	0.012
		0.942	0.040	0.440	0.515	0.039	0.937	0.967
		0.005	0.262	0.038	0.017	0.263	0.000	0.000
	0.2	0.822	1.000	0.999	1.000	1.000	1.000	1.000
		0.198	0.686	0.365	0.316	0.687	0.203	0.200
		0.069	0.127	0.100	0.057	0.127	0.013	0.013
		0.067	0.149	0.078	0.062	0.149	0.013	0.014
		0.942	0.049	0.458	0.523	0.049	0.944	0.963
		0.005	0.252	0.037	0.017	0.253	0.000	0.000
500	0.0	0.051	1.000	0.996	0.994	1.000	0.114	0.037
		-0.001	0.465	0.165	0.116	0.465	0.004	0.002
		0.029	0.055	0.075	0.026	0.055	0.006	0.006
		0.030	0.065	0.035	0.027	0.065	0.006	0.007
		0.949	0.000	0.004	0.006	0.000	0.886	0.963
		0.001	0.219	0.033	0.014	0.219	0.000	0.000
	0.2	1.000	1.000	1.000	1.000	1.000	1.000	1.000
		0.195	0.646	0.360	0.313	0.647	0.202	0.202
		0.029	0.056	0.073	0.026	0.055	0.007	0.007
		0.030	0.065	0.035	0.028	0.065	0.007	0.008
		0.947	0.000	0.004	0.009	0.000	0.929	0.957
		0.001	0.202	0.031	0.013	0.203	0.000	0.000

Table Q. In each cell, from top to bottom are empirical type-I error/power, mean($\hat{\theta}$), SD($\hat{\theta}$), mean(SE($\hat{\theta}$)), coverage rate, MSE, when $n = 50000$, p_invalid=0.7.

m	θ	oracle	default	random	ivw	radial	mixIE-MA-DP	cML-MA-DP
30	0.0	0.053	0.857	0.368	0.358	0.861	0.044	0.046
		-0.011	1.176	0.341	0.261	1.177	0.058	0.005
		0.168	0.363	0.229	0.157	0.363	0.139	0.034
		0.164	0.387	0.204	0.167	0.387	0.103	0.033
		0.938	0.123	0.609	0.642	0.120	0.956	0.954
		0.028	1.515	0.168	0.093	1.518	0.023	0.001
	0.2	0.202	0.947	0.737	0.780	0.948	0.750	0.918
		0.188	1.367	0.539	0.460	1.370	0.272	0.188
		0.168	0.364	0.229	0.157	0.364	0.155	0.051
		0.164	0.389	0.204	0.167	0.388	0.118	0.046
		0.938	0.127	0.612	0.644	0.124	0.950	0.924
		0.028	1.495	0.167	0.093	1.500	0.029	0.003
100	0.0	0.053	1.000	0.906	0.861	1.000	0.052	0.046
		-0.005	1.148	0.380	0.268	1.150	0.020	0.008
		0.090	0.190	0.186	0.081	0.190	0.024	0.021
		0.088	0.207	0.113	0.091	0.207	0.028	0.022
		0.942	0.000	0.090	0.139	0.000	0.948	0.954
		0.008	1.355	0.179	0.079	1.358	0.001	0.001
	0.2	0.577	1.000	1.000	1.000	1.000	0.998	1.000
		0.194	1.338	0.577	0.467	1.340	0.223	0.206
		0.090	0.191	0.185	0.081	0.191	0.029	0.028
		0.088	0.208	0.113	0.091	0.208	0.033	0.027
		0.945	0.000	0.093	0.142	0.000	0.937	0.934
		0.008	1.332	0.177	0.078	1.336	0.001	0.001
500	0.0	0.044	1.000	1.000	1.000	1.000	0.421	0.205
		0.001	1.077	0.381	0.270	1.077	0.035	0.020
		0.039	0.084	0.169	0.037	0.084	0.028	0.016
		0.039	0.091	0.050	0.041	0.091	0.020	0.016
		0.956	0.000	0.000	0.000	0.000	0.579	0.795
		0.001	1.166	0.173	0.074	1.167	0.002	0.001
	0.2	0.999	1.000	1.000	1.000	1.000	0.997	1.000
		0.197	1.258	0.575	0.467	1.259	0.238	0.228
		0.039	0.084	0.166	0.038	0.084	0.023	0.018
		0.039	0.092	0.051	0.041	0.092	0.023	0.018
		0.952	0.000	0.000	0.000	0.000	0.628	0.686
		0.002	1.126	0.169	0.073	1.129	0.002	0.001

Table R. In each cell, from top to bottom are empirical type-I error/power, mean($\hat{\theta}$), SD($\hat{\theta}$), mean(SE($\hat{\theta}$)), coverage rate, MSE, when $n = 50000$, p_invalid=1.

m	θ	oracle	default	random	ivw	radial	mixIE-MA-DP	cML-MA-DP
30	0.0	0.061	0.978	0.532	0.509	0.979	0.617	0.305
		-0.009	1.664	0.513	0.377	1.666	1.078	0.095
		0.174	0.411	0.293	0.181	0.410	0.561	0.208
		0.171	0.427	0.237	0.196	0.427	0.482	0.090
		0.929	0.016	0.442	0.491	0.014	0.383	0.695
		0.030	2.939	0.349	0.174	2.944	1.476	0.052
	0.2	0.185	0.995	0.808	0.830	0.995	0.716	0.380
		0.190	1.855	0.711	0.576	1.857	1.279	0.150
		0.174	0.412	0.292	0.181	0.411	0.576	0.273
		0.171	0.429	0.238	0.196	0.428	0.502	0.105
		0.932	0.018	0.439	0.495	0.016	0.415	0.463
		0.030	2.908	0.346	0.174	2.914	1.496	0.077
100	0.0	0.061	1.000	0.973	0.958	1.000	0.791	0.349
		-0.004	1.645	0.539	0.386	1.646	1.333	0.136
		0.095	0.219	0.247	0.093	0.219	0.372	0.178
		0.092	0.230	0.131	0.107	0.230	0.465	0.086
		0.937	0.000	0.027	0.042	0.000	0.209	0.651
		0.009	2.752	0.351	0.157	2.756	1.914	0.050
	0.2	0.536	1.000	1.000	1.000	1.000	0.891	0.525
		0.194	1.834	0.736	0.585	1.836	1.556	0.248
		0.095	0.220	0.245	0.094	0.220	0.363	0.236
		0.092	0.231	0.131	0.107	0.230	0.460	0.100
		0.937	0.000	0.027	0.043	0.000	0.202	0.522
		0.009	2.719	0.348	0.157	2.724	1.970	0.058
500	0.0	0.050	1.000	1.000	1.000	1.000	1.000	0.932
		0.000	1.541	0.533	0.385	1.541	1.542	0.385
		0.041	0.096	0.223	0.042	0.096	0.096	0.113
		0.041	0.102	0.058	0.048	0.102	0.120	0.073
		0.950	0.000	0.000	0.000	0.000	0.000	0.068
		0.002	2.382	0.333	0.150	2.384	2.386	0.161
	0.2	0.996	1.000	1.000	1.000	1.000	1.000	0.995
		0.196	1.722	0.728	0.582	1.723	1.723	0.551
		0.041	0.096	0.220	0.043	0.096	0.097	0.088
		0.041	0.103	0.058	0.048	0.103	0.123	0.060
		0.947	0.000	0.000	0.000	0.000	0.000	0.024
		0.002	2.326	0.327	0.148	2.328	2.329	0.131

Table S. In each cell, from top to bottom are empirical type-I error/power, mean($\hat{\theta}$), SD($\hat{\theta}$), mean(SE($\hat{\theta}$)), coverage rate, MSE, when $n = 1e + 05$, p_invalid=0.

m	θ	oracle	default	random	ivw	radial	mixIE-MA-DP	cML-MA-DP
30	0.0	0.035<br>0.000<br>0.012<br>0.012<br>0.956<br>0.000	0.037<br>0.000<br>0.027<br>0.028<br>0.954<br>0.001	0.029<br>0.000<br>0.013<br>0.013<br>0.959<br>0.000	0.039<br>0.000<br>0.010<br>0.011<br>0.961<br>0.000	0.056<br>0.000<br>0.027<br>0.026<br>0.934<br>0.001	0.038<br>0.000<br>0.011<br>0.011<br>0.962<br>0.000	0.028<br>0.000<br>0.010<br>0.011<br>0.972<br>0.000
	0.2	1.000<br>0.199<br>0.014<br>0.014<br>0.954<br>0.000	1.000<br>0.196<br>0.031<br>0.031<br>0.943<br>0.001	1.000<br>0.199<br>0.015<br>0.015<br>0.956<br>0.000	1.000<br>0.200<br>0.012<br>0.012<br>0.955<br>0.000	1.000<br>0.196<br>0.031<br>0.029<br>0.929<br>0.001	1.000<br>0.200<br>0.012<br>0.012<br>0.952<br>0.000	1.000<br>0.199<br>0.012<br>0.013<br>0.958<br>0.000
100	0.0	0.049<br>0.000<br>0.006<br>0.007<br>0.946<br>0.000	0.038<br>0.000<br>0.014<br>0.014<br>0.960<br>0.000	0.046<br>0.000<br>0.007<br>0.007<br>0.948<br>0.000	0.049<br>0.000<br>0.006<br>0.006<br>0.951<br>0.000	0.049<br>0.000<br>0.014<br>0.014<br>0.945<br>0.000	0.049<br>0.000<br>0.006<br>0.006<br>0.951<br>0.000	0.032<br>0.000<br>0.006<br>0.006<br>0.968<br>0.000
	0.2	1.000<br>0.199<br>0.007<br>0.007<br>0.950<br>0.000	1.000<br>0.194<br>0.016<br>0.016<br>0.940<br>0.000	1.000<br>0.199<br>0.008<br>0.008<br>0.952<br>0.000	1.000<br>0.199<br>0.006<br>0.006<br>0.949<br>0.000	1.000<br>0.194<br>0.016<br>0.016<br>0.934<br>0.000	1.000<br>0.199<br>0.006<br>0.006<br>0.947<br>0.000	1.000<br>0.199<br>0.007<br>0.007<br>0.963<br>0.000
500	0.0	0.044<br>0.000<br>0.003<br>0.003<br>0.954<br>0.000	0.043<br>0.000<br>0.006<br>0.006<br>0.957<br>0.000	0.048<br>0.000<br>0.003<br>0.003<br>0.952<br>0.000	0.049<br>0.000<br>0.002<br>0.002<br>0.951<br>0.000	0.048<br>0.000<br>0.006<br>0.006<br>0.952<br>0.000	0.053<br>0.000<br>0.002<br>0.003<br>0.947<br>0.000	0.028<br>0.000<br>0.002<br>0.003<br>0.972<br>0.000
	0.2	1.000<br>0.198<br>0.003<br>0.003<br>0.894<br>0.000	1.000<br>0.190<br>0.007<br>0.007<br>0.732<br>0.000	1.000<br>0.197<br>0.004<br>0.004<br>0.880<br>0.000	1.000<br>0.198<br>0.003<br>0.003<br>0.903<br>0.000	1.000<br>0.190<br>0.007<br>0.007<br>0.729<br>0.000	1.000<br>0.198<br>0.003<br>0.003<br>0.892<br>0.000	1.000<br>0.199<br>0.003<br>0.003<br>0.951<br>0.000

Table T. In each cell, from top to bottom are empirical type-I error/power, mean($\hat{\theta}$), SD($\hat{\theta}$), mean(SE($\hat{\theta}$)), coverage rate, MSE, when $n = 1e + 05$, p_invalid=0.3.

m	θ	oracle	default	random	ivw	radial	mixIE-MA-DP	cML-MA-DP
30	0.0	0.059	0.419	0.186	0.191	0.418	0.042	0.032
		0.001	0.510	0.162	0.118	0.511	0.003	0.001
		0.126	0.240	0.150	0.103	0.240	0.013	0.013
		0.121	0.274	0.137	0.110	0.274	0.014	0.014
		0.930	0.545	0.781	0.809	0.545	0.958	0.968
		0.016	0.318	0.049	0.025	0.318	0.000	0.000
	0.2	0.384	0.761	0.735	0.823	0.762	1.000	1.000
		0.201	0.706	0.361	0.318	0.707	0.203	0.200
		0.126	0.241	0.150	0.103	0.240	0.015	0.015
		0.121	0.275	0.137	0.110	0.275	0.016	0.016
		0.932	0.551	0.787	0.812	0.549	0.953	0.964
		0.016	0.313	0.048	0.025	0.315	0.000	0.000
100	0.0	0.046	0.968	0.560	0.503	0.968	0.052	0.032
		0.001	0.502	0.164	0.118	0.502	0.002	0.001
		0.065	0.125	0.100	0.056	0.125	0.008	0.007
		0.066	0.148	0.075	0.060	0.148	0.008	0.008
		0.953	0.029	0.429	0.497	0.029	0.948	0.968
		0.004	0.267	0.037	0.017	0.268	0.000	0.000
	0.2	0.853	1.000	0.994	1.000	1.000	1.000	1.000
		0.200	0.696	0.363	0.317	0.697	0.201	0.200
		0.065	0.125	0.099	0.056	0.125	0.009	0.009
		0.066	0.148	0.075	0.060	0.148	0.009	0.009
		0.953	0.035	0.439	0.501	0.031	0.950	0.965
		0.004	0.261	0.036	0.017	0.263	0.000	0.000
500	0.0	0.047	1.000	0.997	0.992	1.000	0.069	0.026
		0.000	0.487	0.168	0.117	0.488	0.002	0.001
		0.030	0.056	0.079	0.026	0.056	0.004	0.004
		0.030	0.066	0.035	0.027	0.066	0.004	0.004
		0.953	0.000	0.003	0.008	0.000	0.931	0.974
		0.001	0.240	0.035	0.014	0.241	0.000	0.000
	0.2	1.000	1.000	1.000	1.000	1.000	1.000	1.000
		0.198	0.677	0.366	0.315	0.678	0.201	0.200
		0.030	0.057	0.078	0.026	0.057	0.005	0.005
		0.030	0.066	0.035	0.027	0.066	0.005	0.005
		0.950	0.000	0.003	0.011	0.000	0.949	0.972
		0.001	0.231	0.033	0.014	0.232	0.000	0.000

Table U. In each cell, from top to bottom are empirical type-I error/power, mean($\hat{\theta}$), SD($\hat{\theta}$), mean(SE($\hat{\theta}$)), coverage rate, MSE, when $n = 1e + 05$, p_invalid=0.7.

m	θ	oracle	default	random	ivw	radial	mixIE-MA-DP	cML-MA-DP
30	0.0	0.056	0.892	0.406	0.396	0.891	0.039	0.052
		0.002	1.182	0.366	0.272	1.184	0.027	0.003
		0.167	0.351	0.232	0.152	0.350	0.066	0.023
		0.162	0.387	0.202	0.166	0.387	0.073	0.023
		0.931	0.095	0.558	0.604	0.094	0.961	0.948
		0.028	1.520	0.187	0.097	1.523	0.005	0.001
	0.2	0.209	0.955	0.783	0.816	0.956	0.848	0.967
		0.202	1.377	0.565	0.472	1.379	0.232	0.191
		0.167	0.351	0.231	0.152	0.350	0.074	0.037
		0.162	0.387	0.202	0.166	0.387	0.077	0.033
		0.930	0.097	0.560	0.613	0.095	0.954	0.920
		0.028	1.508	0.187	0.097	1.514	0.006	0.001
100	0.0	0.056	1.000	0.894	0.867	1.000	0.050	0.050
		0.004	1.160	0.389	0.275	1.162	0.008	0.003
		0.089	0.187	0.197	0.084	0.187	0.015	0.014
		0.088	0.207	0.112	0.090	0.207	0.018	0.014
		0.940	0.000	0.099	0.133	0.000	0.950	0.950
		0.008	1.382	0.190	0.083	1.385	0.000	0.000
	0.2	0.638	1.000	1.000	1.000	1.000	0.999	1.000
		0.204	1.355	0.588	0.475	1.357	0.210	0.200
		0.089	0.188	0.197	0.084	0.187	0.017	0.018
		0.088	0.207	0.112	0.090	0.207	0.020	0.018
		0.940	0.000	0.101	0.132	0.000	0.956	0.939
		0.008	1.368	0.189	0.082	1.373	0.000	0.000
500	0.0	0.042	1.000	1.000	1.000	1.000	0.245	0.105
		0.001	1.137	0.390	0.274	1.137	0.017	0.008
		0.039	0.084	0.176	0.036	0.084	0.020	0.010
		0.039	0.092	0.051	0.041	0.092	0.014	0.010
		0.957	0.000	0.000	0.000	0.000	0.755	0.895
		0.002	1.299	0.183	0.076	1.300	0.001	0.000
	0.2	0.999	1.000	1.000	1.000	1.000	0.996	1.000
		0.200	1.326	0.588	0.473	1.327	0.218	0.210
		0.039	0.084	0.175	0.036	0.084	0.012	0.012
		0.039	0.093	0.051	0.041	0.093	0.014	0.012
		0.959	0.000	0.000	0.000	0.000	0.769	0.852
		0.002	1.276	0.181	0.076	1.278	0.000	0.000

Table V. In each cell, from top to bottom are empirical type-I error/power, mean($\hat{\theta}$), SD($\hat{\theta}$), mean(SE($\hat{\theta}$)), coverage rate, MSE, when $n = 1e + 05$, p_invalid=1.

m	θ	oracle	default	random	ivw	radial	mixIE-MA-DP	cML-MA-DP
30	0.0	0.065	0.976	0.535	0.517	0.976	0.695	0.341
30	0.0	0.002	1.703	0.512	0.387	1.705	1.147	0.059
30	0.0	0.177	0.415	0.276	0.177	0.415	0.531	0.166
30	0.0	0.169	0.428	0.240	0.195	0.428	0.452	0.066
30	0.0	0.929	0.019	0.434	0.483	0.019	0.305	0.659
30	0.0	0.031	3.073	0.338	0.181	3.079	1.598	0.031
30	0.2	0.205	0.991	0.821	0.859	0.991	0.794	0.352
30	0.2	0.202	1.898	0.712	0.587	1.901	1.332	0.093
30	0.2	0.177	0.416	0.275	0.176	0.415	0.566	0.228
30	0.2	0.170	0.429	0.240	0.195	0.428	0.469	0.080
30	0.2	0.928	0.019	0.431	0.482	0.019	0.346	0.360
30	0.2	0.031	3.057	0.338	0.181	3.064	1.601	0.064
100	0.0	0.053	1.000	0.972	0.949	1.000	0.737	0.304
100	0.0	0.003	1.661	0.546	0.392	1.663	1.153	0.081
100	0.0	0.094	0.223	0.251	0.098	0.223	0.367	0.141
100	0.0	0.092	0.230	0.129	0.106	0.229	0.440	0.063
100	0.0	0.945	0.000	0.027	0.051	0.000	0.263	0.696
100	0.0	0.009	2.810	0.361	0.163	2.814	1.465	0.026
100	0.2	0.581	1.000	0.999	1.000	1.000	0.842	0.395
100	0.2	0.203	1.856	0.745	0.592	1.857	1.401	0.144
100	0.2	0.094	0.224	0.250	0.098	0.223	0.387	0.203
100	0.2	0.092	0.230	0.129	0.106	0.230	0.460	0.075
100	0.2	0.944	0.000	0.026	0.052	0.000	0.277	0.401
100	0.2	0.009	2.791	0.359	0.163	2.796	1.593	0.044
500	0.0	0.042	1.000	1.000	1.000	1.000	1.000	0.754
500	0.0	0.002	1.624	0.553	0.391	1.624	1.624	0.282
500	0.0	0.041	0.099	0.244	0.041	0.099	0.100	0.145
500	0.0	0.041	0.102	0.059	0.048	0.102	0.118	0.076
500	0.0	0.958	0.000	0.000	0.000	0.000	0.000	0.246
500	0.0	0.002	2.647	0.365	0.155	2.649	2.647	0.101
500	0.2	1.000	1.000	1.000	1.000	1.000	1.000	0.940
500	0.2	0.200	1.814	0.750	0.590	1.814	1.814	0.449
500	0.2	0.041	0.099	0.242	0.041	0.099	0.100	0.141
500	0.2	0.041	0.103	0.059	0.048	0.103	0.118	0.069
500	0.2	0.964	0.000	0.000	0.000	0.000	0.000	0.190
500	0.2	0.002	2.614	0.361	0.154	2.616	2.613	0.082

B.4 Simulation (b): balanced pleiotropy

Table W. In each cell, from top to bottom are empirical type-I error/power, mean($\hat{\theta}$), SD($\hat{\theta}$), mean(SE($\hat{\theta}$)), coverage rate, MSE, when $n = 50000$, p_invalid=0.3.

m	θ	oracle	default	random	ivw	radial	mixIE-MA-DP	cML-MA-DP
30	0.0	0.052	0.048	0.049	0.050	0.048	0.057	0.038
		-0.003	-0.007	-0.002	-0.003	-0.007	0.000	0.000
		0.096	0.206	0.104	0.082	0.206	0.021	0.019
		0.093	0.204	0.099	0.080	0.204	0.021	0.020
		0.935	0.943	0.939	0.950	0.944	0.943	0.962
		0.009	0.042	0.011	0.007	0.042	0.000	0.000
	0.2	0.549	0.151	0.543	0.686	0.150	1.000	1.000
		0.196	0.185	0.197	0.196	0.185	0.198	0.196
		0.097	0.207	0.105	0.082	0.207	0.024	0.024
		0.093	0.205	0.100	0.080	0.205	0.024	0.024
		0.935	0.943	0.938	0.948	0.942	0.944	0.949
		0.009	0.043	0.011	0.007	0.043	0.001	0.001
100	0.0	0.051	0.061	0.057	0.058	0.060	0.060	0.037
		-0.002	0.001	-0.001	-0.001	0.001	0.000	0.000
		0.053	0.114	0.060	0.045	0.114	0.012	0.011
		0.051	0.111	0.057	0.044	0.111	0.012	0.012
		0.945	0.937	0.939	0.942	0.937	0.940	0.963
		0.003	0.013	0.004	0.002	0.013	0.000	0.000
	0.2	0.955	0.407	0.898	0.992	0.408	1.000	1.000
		0.196	0.191	0.196	0.198	0.192	0.199	0.199
		0.053	0.115	0.060	0.046	0.115	0.014	0.013
		0.051	0.112	0.057	0.045	0.112	0.013	0.014
		0.946	0.937	0.942	0.941	0.937	0.939	0.955
		0.003	0.013	0.004	0.002	0.013	0.000	0.000
500	0.0	0.058	0.046	0.060	0.059	0.046	0.054	0.030
		-0.001	0.000	-0.001	-0.001	0.000	0.000	0.000
		0.023	0.048	0.026	0.020	0.048	0.006	0.006
		0.023	0.049	0.025	0.020	0.049	0.006	0.006
		0.942	0.954	0.940	0.941	0.954	0.946	0.970
		0.001	0.002	0.001	0.000	0.002	0.000	0.000
	0.2	1.000	0.957	1.000	1.000	0.958	1.000	1.000
		0.195	0.182	0.194	0.196	0.182	0.197	0.199
		0.023	0.048	0.026	0.020	0.048	0.007	0.007
		0.023	0.049	0.025	0.020	0.049	0.006	0.007
		0.940	0.935	0.941	0.945	0.936	0.911	0.955
		0.001	0.003	0.001	0.000	0.003	0.000	0.000

Table X. In each cell, from top to bottom are empirical type-I error/power, mean($\hat{\theta}$), SD($\hat{\theta}$), mean(SE($\hat{\theta}$)), coverage rate, MSE, when $n = 50000$, p_invalid=0.7.

m	θ	oracle	default	random	ivw	radial	mixIE-MA-DP	cML-MA-DP
30	0.0	0.049	0.059	0.047	0.054	0.059	0.046	0.059
		-0.011	0.010	-0.011	-0.007	0.010	-0.002	-0.002
		0.146	0.325	0.161	0.124	0.325	0.047	0.035
		0.143	0.314	0.151	0.123	0.314	0.052	0.033
		0.939	0.930	0.940	0.946	0.930	0.954	0.941
		0.021	0.105	0.026	0.015	0.105	0.002	0.001
	0.2	0.261	0.100	0.260	0.351	0.100	0.876	0.799
		0.188	0.203	0.188	0.192	0.204	0.195	0.166
		0.146	0.325	0.161	0.124	0.325	0.054	0.058
		0.143	0.315	0.151	0.123	0.315	0.058	0.051
		0.942	0.928	0.940	0.945	0.927	0.943	0.844
		0.022	0.106	0.026	0.015	0.106	0.003	0.005
100	0.0	0.055	0.063	0.049	0.061	0.063	0.040	0.053
		-0.006	0.001	-0.003	-0.005	0.001	0.001	0.001
		0.078	0.173	0.089	0.069	0.173	0.024	0.022
		0.077	0.169	0.084	0.067	0.169	0.027	0.022
		0.943	0.936	0.950	0.939	0.936	0.960	0.947
		0.006	0.030	0.008	0.005	0.030	0.001	0.000
	0.2	0.693	0.203	0.650	0.813	0.204	1.000	0.995
		0.193	0.192	0.195	0.194	0.193	0.199	0.192
		0.078	0.173	0.089	0.069	0.173	0.027	0.029
		0.077	0.169	0.085	0.067	0.169	0.031	0.028
		0.941	0.939	0.948	0.941	0.938	0.962	0.933
		0.006	0.030	0.008	0.005	0.030	0.001	0.001
500	0.0	0.051	0.047	0.054	0.053	0.046	0.020	0.032
		0.000	-0.005	-0.002	0.000	-0.005	0.000	0.000
		0.035	0.073	0.038	0.031	0.073	0.013	0.013
		0.035	0.074	0.038	0.030	0.074	0.016	0.014
		0.948	0.952	0.946	0.947	0.952	0.980	0.968
		0.001	0.005	0.001	0.001	0.005	0.000	0.000
	0.2	1.000	0.653	0.981	1.000	0.656	1.000	1.000
		0.197	0.177	0.193	0.197	0.178	0.196	0.200
		0.035	0.074	0.039	0.031	0.074	0.015	0.015
		0.035	0.074	0.038	0.030	0.074	0.017	0.015
		0.952	0.939	0.940	0.945	0.940	0.982	0.959
		0.001	0.006	0.002	0.001	0.006	0.000	0.000

Table Y. In each cell, from top to bottom are empirical type-I error/power, mean($\hat{\theta}$), SD($\hat{\theta}$), mean(SE($\hat{\theta}$)), coverage rate, MSE, when $n = 50000$, p_invalid=1.

m	θ	oracle	default	random	ivw	radial	mixIE-MA-DP	cML-MA-DP
30	0.0	0.061	0.055	0.057	0.069	0.054	0.176	0.172
		-0.009	0.005	-0.003	-0.007	0.005	-0.015	0.002
		0.174	0.375	0.196	0.149	0.375	0.278	0.119
		0.171	0.375	0.181	0.147	0.375	0.260	0.078
		0.929	0.938	0.928	0.931	0.936	0.824	0.828
		0.030	0.140	0.038	0.022	0.140	0.078	0.014
	0.2	0.187	0.075	0.198	0.278	0.074	0.207	0.196
		0.190	0.197	0.196	0.192	0.198	0.169	0.040
		0.174	0.375	0.196	0.149	0.375	0.269	0.142
		0.171	0.375	0.181	0.147	0.375	0.262	0.085
		0.930	0.936	0.928	0.929	0.936	0.844	0.396
		0.030	0.141	0.039	0.022	0.140	0.073	0.046
100	0.0	0.065	0.057	0.065	0.068	0.057	0.073	0.126
		-0.005	0.007	0.000	-0.002	0.007	-0.011	-0.001
		0.095	0.202	0.106	0.083	0.202	0.238	0.098
		0.092	0.201	0.100	0.080	0.201	0.283	0.073
		0.931	0.941	0.931	0.932	0.941	0.927	0.874
		0.009	0.041	0.011	0.007	0.041	0.057	0.010
	0.2	0.533	0.160	0.534	0.686	0.162	0.111	0.180
		0.194	0.198	0.198	0.196	0.199	0.177	0.053
		0.095	0.203	0.106	0.084	0.203	0.223	0.115
		0.092	0.202	0.100	0.080	0.201	0.270	0.082
		0.932	0.941	0.932	0.930	0.940	0.937	0.475
		0.009	0.041	0.011	0.007	0.041	0.050	0.035
500	0.0	0.043	0.039	0.053	0.056	0.039	0.026	0.105
		0.000	-0.004	0.000	-0.001	-0.004	-0.003	-0.005
		0.041	0.087	0.047	0.036	0.087	0.089	0.089
		0.041	0.088	0.045	0.036	0.088	0.109	0.078
		0.954	0.961	0.947	0.944	0.960	0.974	0.895
		0.002	0.008	0.002	0.001	0.008	0.008	0.008
	0.2	0.999	0.517	0.954	1.000	0.519	0.355	0.365
		0.196	0.178	0.195	0.196	0.179	0.178	0.131
		0.041	0.088	0.047	0.036	0.088	0.091	0.102
		0.041	0.088	0.045	0.036	0.088	0.111	0.081
		0.947	0.952	0.941	0.940	0.954	0.975	0.733
		0.002	0.008	0.002	0.001	0.008	0.009	0.015

Table Z. In each cell, from top to bottom are empirical type-I error/power, mean($\hat{\theta}$), SD($\hat{\theta}$), mean(SE($\hat{\theta}$)), coverage rate, MSE, when $n = 1e + 05$, p_invalid=0.3.

m	θ	oracle	default	random	ivw	radial	mixIE-MA-DP	cML-MA-DP
30	0.0	0.039	0.051	0.049	0.055	0.051	0.042	0.034
		0.002	0.000	0.004	0.002	0.000	0.001	0.001
		0.094	0.211	0.104	0.079	0.211	0.014	0.013
		0.091	0.202	0.098	0.078	0.202	0.014	0.014
		0.949	0.942	0.937	0.945	0.942	0.958	0.966
		0.009	0.045	0.011	0.006	0.045	0.000	0.000
	0.2	0.570	0.187	0.574	0.731	0.187	1.000	1.000
		0.201	0.196	0.203	0.202	0.197	0.200	0.198
		0.094	0.212	0.104	0.079	0.211	0.016	0.016
		0.091	0.202	0.098	0.078	0.202	0.016	0.017
		0.951	0.944	0.939	0.946	0.944	0.961	0.964
		0.009	0.045	0.011	0.006	0.045	0.000	0.000
100	0.0	0.050	0.050	0.048	0.054	0.050	0.038	0.029
		0.002	-0.005	0.000	0.000	-0.005	0.000	0.000
		0.049	0.111	0.056	0.044	0.111	0.008	0.007
		0.050	0.111	0.055	0.044	0.111	0.008	0.008
		0.945	0.948	0.950	0.946	0.947	0.962	0.971
		0.002	0.012	0.003	0.002	0.012	0.000	0.000
	0.2	0.971	0.393	0.911	0.989	0.395	1.000	1.000
		0.201	0.189	0.198	0.200	0.190	0.199	0.199
		0.049	0.111	0.056	0.044	0.111	0.009	0.009
		0.050	0.111	0.055	0.044	0.111	0.009	0.009
		0.949	0.947	0.947	0.946	0.947	0.955	0.962
		0.002	0.012	0.003	0.002	0.012	0.000	0.000
500	0.0	0.051	0.052	0.049	0.052	0.052	0.049	0.029
		0.000	-0.003	-0.001	0.000	-0.003	0.000	0.000
		0.023	0.049	0.026	0.020	0.049	0.004	0.004
		0.023	0.049	0.025	0.020	0.049	0.004	0.004
		0.949	0.947	0.951	0.948	0.948	0.951	0.971
		0.001	0.002	0.001	0.000	0.002	0.000	0.000
	0.2	1.000	0.966	1.000	1.000	0.966	1.000	1.000
		0.198	0.188	0.197	0.198	0.188	0.198	0.199
		0.023	0.049	0.026	0.020	0.049	0.004	0.004
		0.023	0.050	0.025	0.020	0.050	0.004	0.005
		0.956	0.943	0.938	0.951	0.943	0.929	0.952
		0.001	0.003	0.001	0.000	0.003	0.000	0.000

Table AA. In each cell, from top to bottom are empirical type-I error/power, mean($\hat{\theta}$), SD($\hat{\theta}$), mean(SE($\hat{\theta}$)), coverage rate, MSE, when $n = 1e + 05$, p_invalid=0.7.

m	θ	oracle	default	random	ivw	radial	mixIE-MA-DP	cML-MA-DP
30	0.0	0.055<br>0.002<br>0.146<br>0.141<br>0.929<br>0.021	0.057<br>-0.007<br>0.321<br>0.313<br>0.931<br>0.103	0.050<br>0.001<br>0.158<br>0.150<br>0.941<br>0.025	0.069<br>0.001<br>0.126<br>0.121<br>0.931<br>0.016	0.058<br>-0.007<br>0.321<br>0.313<br>0.931<br>0.103	0.042<br>-0.002<br>0.037<br>0.037<br>0.958<br>0.001	0.068<br>-0.001<br>0.025<br>0.023<br>0.932<br>0.001
	0.2	0.274<br>0.201<br>0.146<br>0.141<br>0.930<br>0.021	0.092<br>0.189<br>0.321<br>0.313<br>0.931<br>0.103	0.274<br>0.200<br>0.158<br>0.151<br>0.939<br>0.025	0.382<br>0.200<br>0.126<br>0.121<br>0.932<br>0.016	0.093<br>0.191<br>0.321<br>0.313<br>0.932<br>0.103	0.955<br>0.197<br>0.042<br>0.041<br>0.953<br>0.002	0.868<br>0.172<br>0.047<br>0.040<br>0.872<br>0.003
100	0.0	0.055<br>0.003<br>0.079<br>0.077<br>0.942<br>0.006	0.058<br>-0.014<br>0.173<br>0.169<br>0.938<br>0.030	0.054<br>-0.001<br>0.091<br>0.084<br>0.940<br>0.008	0.060<br>-0.001<br>0.069<br>0.067<br>0.940<br>0.005	0.057<br>-0.014<br>0.173<br>0.169<br>0.939<br>0.030	0.030<br>-0.001<br>0.016<br>0.018<br>0.970<br>0.000	0.044<br>-0.001<br>0.015<br>0.014<br>0.956<br>0.000
	0.2	0.739<br>0.203<br>0.079<br>0.077<br>0.943<br>0.006	0.198<br>0.181<br>0.173<br>0.170<br>0.936<br>0.030	0.666<br>0.198<br>0.091<br>0.084<br>0.941<br>0.008	0.840<br>0.199<br>0.069<br>0.067<br>0.940<br>0.005	0.200<br>0.182<br>0.173<br>0.170<br>0.937<br>0.030	1.000<br>0.198<br>0.018<br>0.020<br>0.967<br>0.000	0.999<br>0.193<br>0.020<br>0.019<br>0.931<br>0.000
500	0.0	0.044<br>0.002<br>0.034<br>0.035<br>0.956<br>0.001	0.051<br>-0.004<br>0.076<br>0.075<br>0.949<br>0.006	0.057<br>0.001<br>0.040<br>0.038<br>0.943<br>0.002	0.053<br>0.001<br>0.030<br>0.030<br>0.947<br>0.001	0.051<br>-0.004<br>0.076<br>0.075<br>0.949<br>0.006	0.019<br>0.000<br>0.008<br>0.010<br>0.981<br>0.000	0.044<br>0.000<br>0.009<br>0.009<br>0.956<br>0.000
	0.2	1.000<br>0.200<br>0.034<br>0.035<br>0.958<br>0.001	0.692<br>0.186<br>0.076<br>0.075<br>0.941<br>0.006	0.985<br>0.198<br>0.040<br>0.038<br>0.943<br>0.002	1.000<br>0.200<br>0.030<br>0.030<br>0.950<br>0.001	0.696<br>0.187<br>0.076<br>0.075<br>0.941<br>0.006	1.000<br>0.198<br>0.010<br>0.011<br>0.977<br>0.000	1.000<br>0.198<br>0.010<br>0.010<br>0.951<br>0.000

Table AB. In each cell, from top to bottom are empirical type-I error/power, mean($\hat{\theta}$), SD($\hat{\theta}$), mean(SE($\hat{\theta}$)), coverage rate, MSE, when $n = 1e + 05$, p_invalid=1.

m	θ	oracle	default	random	ivw	radial	mixIE-MA-DP	cML-MA-DP
30	0.0	0.065	0.057	0.045	0.063	0.058	0.211	0.223
		0.002	0.001	-0.006	0.002	0.001	-0.005	-0.001
		0.177	0.391	0.191	0.152	0.391	0.326	0.105
		0.169	0.377	0.182	0.146	0.377	0.269	0.058
		0.929	0.928	0.946	0.937	0.927	0.789	0.777
		0.031	0.153	0.036	0.023	0.153	0.106	0.011
	0.2	0.198	0.086	0.197	0.296	0.086	0.251	0.226
		0.202	0.198	0.194	0.202	0.200	0.178	0.024
		0.177	0.391	0.191	0.152	0.391	0.305	0.114
		0.170	0.377	0.182	0.146	0.377	0.266	0.062
		0.928	0.931	0.944	0.935	0.931	0.811	0.239
		0.031	0.153	0.036	0.023	0.153	0.094	0.044
100	0.0	0.053	0.051	0.064	0.057	0.051	0.120	0.163
		0.003	-0.012	0.000	0.000	-0.012	-0.004	0.000
		0.094	0.208	0.108	0.082	0.208	0.283	0.083
		0.092	0.203	0.100	0.080	0.203	0.322	0.057
		0.943	0.946	0.933	0.943	0.945	0.880	0.837
		0.009	0.044	0.012	0.007	0.044	0.080	0.007
	0.2	0.583	0.154	0.540	0.701	0.154	0.142	0.179
		0.203	0.183	0.199	0.199	0.184	0.182	0.032
		0.094	0.209	0.108	0.082	0.209	0.260	0.092
		0.092	0.203	0.100	0.080	0.203	0.311	0.062
		0.944	0.944	0.934	0.944	0.943	0.900	0.285
		0.009	0.044	0.012	0.007	0.044	0.068	0.037
500	0.0	0.036	0.058	0.044	0.043	0.058	0.033	0.109
		0.002	-0.004	0.000	0.002	-0.004	-0.003	0.003
		0.041	0.092	0.046	0.036	0.092	0.092	0.073
		0.041	0.090	0.045	0.036	0.090	0.106	0.064
		0.964	0.941	0.956	0.957	0.940	0.967	0.891
		0.002	0.008	0.002	0.001	0.008	0.008	0.005
	0.2	1.000	0.546	0.967	1.000	0.553	0.403	0.256
		0.200	0.187	0.198	0.200	0.188	0.187	0.084
		0.041	0.092	0.046	0.036	0.092	0.092	0.096
		0.041	0.090	0.045	0.036	0.090	0.106	0.070
		0.966	0.939	0.958	0.956	0.937	0.968	0.528
		0.002	0.009	0.002	0.001	0.009	0.009	0.023

B.5 Simulation (c): directional pleiotropy

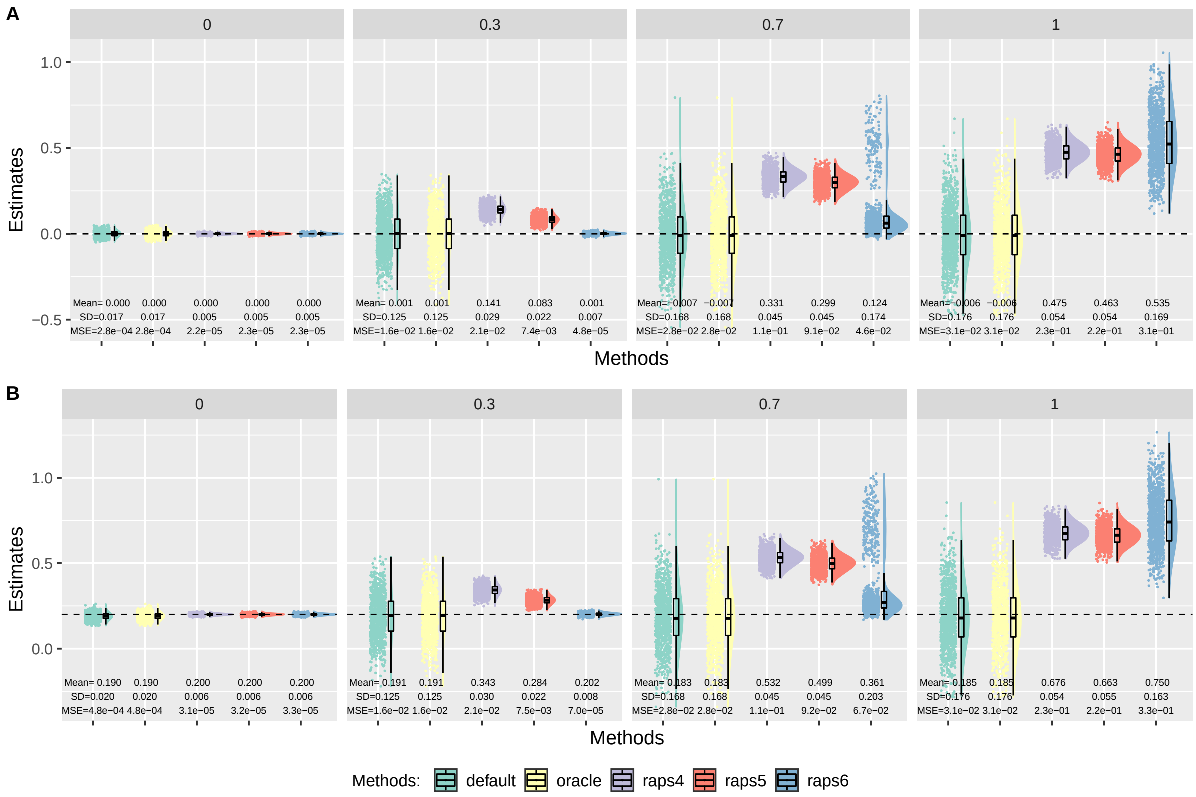

Fig F. Simulation (c) results with directional pleiotropy, $n = 50\,000, m = 100$. Empirical distributions of the estimates of the causal effect θ by the methods. raps4, raps5, raps6 correspond to the three versions of MR-RAPS with an over-dispersed model and with the L2, Huber and Tukey loss functions respectively. Each column corresponds to 0%, 30%, 70% or 100% invalid IVs. A: $\theta = 0$. B: $\theta = 0.2$.

Table AC. In each cell, from top to bottom are empirical type-I error/power, mean($\hat{\theta}$), SD($\hat{\theta}$), mean(SE($\hat{\theta}$)), coverage rate, MSE, when $n = 50000$, p_invalid=0.

m	θ	oracle	default	random	ivw	radial	mixIE-MA-DP	cML-MA-DP
30	0.0	0.036	0.036	0.023	0.029	0.046	0.026	0.022
		0.001	0.001	0.000	0.000	0.001	0.000	0.000
		0.032	0.032	0.011	0.008	0.032	0.008	0.008
		0.033	0.033	0.012	0.009	0.031	0.009	0.009
		0.955	0.955	0.971	0.971	0.945	0.974	0.979
		0.001	0.001	0.000	0.000	0.001	0.000	0.000
	0.2	0.997	0.997	1.000	1.000	0.997	1.000	1.000
		0.195	0.195	0.200	0.200	0.195	0.199	0.199
		0.036	0.036	0.013	0.010	0.036	0.010	0.010
		0.037	0.037	0.013	0.010	0.035	0.011	0.011
		0.954	0.954	0.959	0.959	0.939	0.966	0.968
		0.001	0.001	0.000	0.000	0.001	0.000	0.000
100	0.0	0.043	0.043	0.036	0.036	0.050	0.040	0.029
		0.000	0.000	0.000	0.000	0.000	0.000	0.000
		0.017	0.017	0.006	0.005	0.017	0.005	0.005
		0.017	0.017	0.006	0.005	0.017	0.005	0.005
		0.953	0.953	0.960	0.964	0.948	0.960	0.971
		0.000	0.000	0.000	0.000	0.000	0.000	0.000
	0.2	1.000	1.000	1.000	1.000	1.000	1.000	1.000
		0.190	0.190	0.199	0.199	0.190	0.199	0.199
		0.020	0.020	0.008	0.006	0.020	0.006	0.006
		0.020	0.020	0.007	0.006	0.019	0.006	0.006
		0.915	0.915	0.942	0.942	0.908	0.946	0.962
		0.000	0.000	0.000	0.000	0.000	0.000	0.000

Table AD. In each cell, from top to bottom are empirical type-I error/power, mean($\hat{\theta}$), SD($\hat{\theta}$), mean(SE($\hat{\theta}$)), coverage rate, MSE, when $n = 50000$, p_invalid=0.3.

m	θ	oracle	default	random	ivw	radial	mixIE-MA-DP	cML-MA-DP
30	0.0	0.048	0.048	0.384	0.653	0.047	0.059	0.035
		-0.007	-0.007	0.127	0.139	-0.001	0.001	0.001
		0.229	0.229	0.076	0.050	0.229	0.013	0.012
		0.229	0.229	0.082	0.063	0.229	0.012	0.012
		0.940	0.940	0.582	0.347	0.939	0.941	0.965
		0.053	0.053	0.022	0.022	0.053	0.000	0.000
	0.2	0.125	0.125	0.930	1.000	0.130	1.000	1.000
		0.186	0.186	0.327	0.338	0.193	0.201	0.200
		0.230	0.230	0.076	0.050	0.231	0.015	0.014
		0.229	0.229	0.082	0.064	0.229	0.014	0.014
		0.936	0.936	0.591	0.355	0.939	0.936	0.953
		0.053	0.053	0.022	0.022	0.053	0.000	0.000
100	0.0	0.049	0.049	0.813	0.998	0.047	0.067	0.047
		0.001	0.001	0.128	0.139	0.006	0.001	0.001
		0.125	0.125	0.049	0.028	0.125	0.007	0.007
		0.123	0.123	0.047	0.035	0.123	0.007	0.007
		0.949	0.949	0.183	0.002	0.949	0.933	0.953
		0.016	0.016	0.019	0.020	0.016	0.000	0.000
	0.2	0.356	0.356	0.986	1.000	0.372	1.000	1.000
		0.191	0.191	0.327	0.339	0.197	0.200	0.201
		0.125	0.125	0.050	0.029	0.125	0.008	0.008
		0.124	0.124	0.047	0.035	0.124	0.008	0.008
		0.950	0.950	0.191	0.002	0.950	0.937	0.951
		0.016	0.016	0.019	0.020	0.016	0.000	0.000

Table AE. In each cell, from top to bottom are empirical type-I error/power, mean($\hat{\theta}$), SD($\hat{\theta}$), mean(SE($\hat{\theta}$)), coverage rate, MSE, when $n = 50000$, p_invalid=0.7.

m	θ	oracle	default	random	ivw	radial	mixIE-MA-DP	cML-MA-DP
30	0.0	0.050	0.050	0.797	0.987	0.050	0.055	0.076
		-0.017	-0.017	0.308	0.326	-0.007	0.049	0.007
		0.314	0.314	0.109	0.078	0.314	0.110	0.021
		0.307	0.307	0.110	0.086	0.308	0.094	0.020
		0.940	0.940	0.186	0.013	0.940	0.945	0.924
		0.099	0.099	0.107	0.112	0.099	0.015	0.000
	0.2	0.086	0.086	0.949	1.000	0.092	0.831	0.995
		0.177	0.177	0.507	0.525	0.188	0.238	0.202
		0.314	0.314	0.109	0.078	0.315	0.092	0.032
		0.308	0.308	0.110	0.087	0.308	0.077	0.027
		0.941	0.941	0.187	0.015	0.942	0.946	0.906
		0.099	0.099	0.106	0.112	0.099	0.010	0.001
100	0.0	0.054	0.054	0.923	1.000	0.054	0.026	0.094
		-0.007	-0.007	0.296	0.323	0.001	0.006	0.009
		0.168	0.168	0.079	0.044	0.168	0.034	0.013
		0.164	0.164	0.062	0.047	0.164	0.024	0.013
		0.943	0.943	0.077	0.000	0.945	0.974	0.906
		0.028	0.028	0.094	0.106	0.028	0.001	0.000
	0.2	0.192	0.192	0.985	1.000	0.209	0.992	1.000
		0.183	0.183	0.495	0.522	0.193	0.203	0.211
		0.168	0.168	0.080	0.044	0.168	0.029	0.018
		0.164	0.164	0.062	0.047	0.164	0.024	0.016
		0.943	0.943	0.077	0.000	0.941	0.970	0.875
		0.028	0.028	0.093	0.106	0.028	0.001	0.000

Table AF. In each cell, from top to bottom are empirical type-I error/power, mean($\hat{\theta}$), SD($\hat{\theta}$), mean(SE($\hat{\theta}$)), coverage rate, MSE, when $n = 50000$, p_invalid=1.

m	θ	oracle	default	random	ivw	radial	mixIE-MA-DP	cML-MA-DP
30	0.0	0.053	0.053	0.898	1.000	0.053	0.426	0.519
		-0.015	-0.015	0.437	0.463	-0.005	0.275	0.146
		0.322	0.322	0.132	0.093	0.323	0.235	0.165
		0.320	0.320	0.118	0.092	0.320	0.228	0.050
		0.943	0.943	0.097	0.000	0.944	0.574	0.481
		0.104	0.104	0.208	0.223	0.104	0.130	0.048
	0.2	0.076	0.076	0.966	1.000	0.088	0.566	0.787
		0.178	0.178	0.636	0.663	0.191	0.461	0.314
		0.323	0.323	0.133	0.093	0.323	0.233	0.210
		0.320	0.320	0.118	0.092	0.321	0.230	0.055
		0.945	0.945	0.098	0.000	0.944	0.593	0.294
		0.105	0.105	0.208	0.223	0.105	0.123	0.057
100	0.0	0.054	0.054	0.962	1.000	0.053	0.147	0.767
		-0.006	-0.006	0.428	0.463	0.003	0.171	0.220
		0.176	0.176	0.096	0.053	0.176	0.179	0.138
		0.171	0.171	0.065	0.050	0.171	0.240	0.051
		0.943	0.943	0.037	0.000	0.944	0.853	0.233
		0.031	0.031	0.192	0.217	0.031	0.061	0.067
	0.2	0.177	0.177	0.991	1.000	0.196	0.305	0.979
		0.185	0.185	0.626	0.662	0.194	0.339	0.413
		0.176	0.176	0.097	0.053	0.176	0.180	0.132
		0.172	0.172	0.066	0.050	0.172	0.239	0.050
		0.940	0.940	0.038	0.000	0.939	0.880	0.165
		0.031	0.031	0.191	0.216	0.031	0.052	0.063

Table AG. In each cell, from top to bottom are empirical type-I error/power, mean($\hat{\theta}$), SD($\hat{\theta}$), mean(SE($\hat{\theta}$)), coverage rate, MSE, when $n = 1e + 05$, p_invalid=0.

m	θ	oracle	default	random	ivw	radial	mixIE-MA-DP	cML-MA-DP
30	0.0	0.048<br>0.000<br>0.023<br>0.023<br>0.946<br>0.001	0.048<br>0.000<br>0.023<br>0.023<br>0.946<br>0.001	0.037<br>0.000<br>0.008<br>0.008<br>0.953<br>0.000	0.044<br>0.000<br>0.006<br>0.006<br>0.956<br>0.000	0.060<br>0.000<br>0.023<br>0.022<br>0.926<br>0.001	0.046<br>0.000<br>0.006<br>0.007<br>0.954<br>0.000	0.038<br>0.000<br>0.006<br>0.007<br>0.962<br>0.000
	0.2	1.000<br>0.196<br>0.026<br>0.026<br>0.948<br>0.001	1.000<br>0.196<br>0.026<br>0.026<br>0.948<br>0.001	1.000<br>0.200<br>0.010<br>0.009<br>0.938<br>0.000	1.000<br>0.200<br>0.007<br>0.007<br>0.943<br>0.000	1.000<br>0.196<br>0.026<br>0.025<br>0.922<br>0.001	1.000<br>0.200<br>0.007<br>0.007<br>0.952<br>0.000	1.000<br>0.200<br>0.007<br>0.008<br>0.956<br>0.000
100	0.0	0.046<br>0.000<br>0.012<br>0.012<br>0.952<br>0.000	0.046<br>0.000<br>0.012<br>0.012<br>0.952<br>0.000	0.043<br>0.000<br>0.005<br>0.005<br>0.957<br>0.000	0.034<br>0.000<br>0.003<br>0.003<br>0.966<br>0.000	0.054<br>0.000<br>0.012<br>0.012<br>0.943<br>0.000	0.037<br>0.000<br>0.003<br>0.003<br>0.963<br>0.000	0.024<br>0.000<br>0.003<br>0.004<br>0.976<br>0.000
	0.2	1.000<br>0.195<br>0.014<br>0.014<br>0.937<br>0.000	1.000<br>0.195<br>0.014<br>0.014<br>0.937<br>0.000	1.000<br>0.199<br>0.005<br>0.005<br>0.944<br>0.000	1.000<br>0.200<br>0.004<br>0.004<br>0.952<br>0.000	1.000<br>0.195<br>0.014<br>0.014<br>0.934<br>0.000	1.000<br>0.200<br>0.004<br>0.004<br>0.955<br>0.000	1.000<br>0.199<br>0.004<br>0.004<br>0.967<br>0.000

Table AH. In each cell, from top to bottom are empirical type-I error/power, mean($\hat{\theta}$), SD($\hat{\theta}$), mean(SE($\hat{\theta}$)), coverage rate, MSE, when $n = 1e + 05$, p_invalid=0.3.

m	θ	oracle	default	random	ivw	radial	mixIE-MA-DP	cML-MA-DP
30	0.0	0.058<br>0.001<br>0.235<br>0.228<br>0.935<br>0.055	0.058<br>0.001<br>0.235<br>0.228<br>0.935<br>0.055	0.416<br>0.133<br>0.080<br>0.082<br>0.542<br>0.024	0.689<br>0.141<br>0.050<br>0.063<br>0.311<br>0.022	0.057<br>0.007<br>0.235<br>0.228<br>0.932<br>0.055	0.043<br>0.001<br>0.008<br>0.008<br>0.957<br>0.000	0.031<br>0.001<br>0.008<br>0.008<br>0.969<br>0.000
	0.2	0.151<br>0.198<br>0.235<br>0.228<br>0.931<br>0.055	0.151<br>0.198<br>0.235<br>0.228<br>0.931<br>0.055	0.937<br>0.332<br>0.080<br>0.082<br>0.545<br>0.024	1.000<br>0.341<br>0.050<br>0.063<br>0.311<br>0.022	0.157<br>0.205<br>0.236<br>0.229<br>0.933<br>0.056	1.000<br>0.201<br>0.009<br>0.009<br>0.949<br>0.000	1.000<br>0.200<br>0.009<br>0.010<br>0.960<br>0.000
100	0.0	0.053<br>0.003<br>0.122<br>0.123<br>0.942<br>0.015	0.053<br>0.003<br>0.122<br>0.123<br>0.942<br>0.015	0.829<br>0.126<br>0.048<br>0.045<br>0.166<br>0.018	0.998<br>0.138<br>0.028<br>0.034<br>0.002<br>0.020	0.054<br>0.008<br>0.122<br>0.123<br>0.943<br>0.015	0.056<br>0.000<br>0.005<br>0.005<br>0.944<br>0.000	0.033<br>0.001<br>0.004<br>0.005<br>0.967<br>0.000
	0.2	0.357<br>0.197<br>0.123<br>0.123<br>0.946<br>0.015	0.357<br>0.197<br>0.123<br>0.123<br>0.946<br>0.015	0.986<br>0.325<br>0.049<br>0.045<br>0.175<br>0.018	1.000<br>0.338<br>0.028<br>0.034<br>0.002<br>0.020	0.374<br>0.204<br>0.123<br>0.123<br>0.946<br>0.015	1.000<br>0.200<br>0.006<br>0.005<br>0.937<br>0.000	1.000<br>0.200<br>0.005<br>0.006<br>0.956<br>0.000

Table AI. In each cell, from top to bottom are empirical type-I error/power, mean($\hat{\theta}$), SD($\hat{\theta}$), mean(SE($\hat{\theta}$)), coverage rate, MSE, when $n = 1e + 05$, p_invalid=0.7.

m	θ	oracle	default	random	ivw	radial	mixIE-MA-DP	cML-MA-DP
30	0.0	0.051<br>0.000<br>0.312<br>0.306<br>0.938<br>0.097	0.051<br>0.000<br>0.312<br>0.306<br>0.938<br>0.097	0.794<br>0.311<br>0.112<br>0.110<br>0.189<br>0.109	0.991<br>0.326<br>0.079<br>0.085<br>0.009<br>0.112	0.049<br>0.010<br>0.312<br>0.307<br>0.939<br>0.098	0.064<br>0.012<br>0.047<br>0.030<br>0.936<br>0.002	0.069<br>0.004<br>0.014<br>0.013<br>0.931<br>0.000
	0.2	0.092<br>0.197<br>0.312<br>0.306<br>0.940<br>0.097	0.092<br>0.197<br>0.312<br>0.306<br>0.940<br>0.097	0.962<br>0.510<br>0.112<br>0.110<br>0.190<br>0.109	1.000<br>0.525<br>0.079<br>0.085<br>0.010<br>0.112	0.096<br>0.208<br>0.312<br>0.307<br>0.937<br>0.097	0.983<br>0.208<br>0.033<br>0.027<br>0.939<br>0.001	0.997<br>0.199<br>0.021<br>0.019<br>0.913<br>0.000
100	0.0	0.058<br>0.008<br>0.169<br>0.164<br>0.937<br>0.029	0.058<br>0.008<br>0.169<br>0.164<br>0.937<br>0.029	0.927<br>0.296<br>0.072<br>0.062<br>0.072<br>0.093	1.000<br>0.321<br>0.042<br>0.047<br>0.000<br>0.105	0.057<br>0.017<br>0.169<br>0.164<br>0.941<br>0.029	0.031<br>0.000<br>0.009<br>0.010<br>0.969<br>0.000	0.054<br>0.003<br>0.009<br>0.009<br>0.946<br>0.000
	0.2	0.240<br>0.204<br>0.169<br>0.164<br>0.935<br>0.029	0.240<br>0.204<br>0.169<br>0.164<br>0.935<br>0.029	0.992<br>0.495<br>0.072<br>0.062<br>0.076<br>0.092	1.000<br>0.520<br>0.043<br>0.047<br>0.000<br>0.104	0.257<br>0.213<br>0.169<br>0.164<br>0.937<br>0.029	1.000<br>0.200<br>0.011<br>0.012<br>0.964<br>0.000	1.000<br>0.203<br>0.011<br>0.011<br>0.928<br>0.000

Table AJ. In each cell, from top to bottom are empirical type-I error/power, mean($\hat{\theta}$), SD($\hat{\theta}$), mean(SE($\hat{\theta}$)), coverage rate, MSE, when $n = 1e + 05$, p_invalid=1.

m	θ	oracle	default	random	ivw	radial	mixIE-MA-DP	cML-MA-DP
30	0.0	0.056	0.056	0.881	0.999	0.056	0.347	0.462
		0.004	0.004	0.435	0.466	0.014	0.250	0.096
		0.332	0.332	0.134	0.095	0.333	0.222	0.134
		0.320	0.320	0.117	0.091	0.321	0.219	0.039
		0.933	0.933	0.109	0.001	0.934	0.653	0.538
		0.110	0.110	0.207	0.226	0.111	0.112	0.027
	0.2	0.095	0.095	0.966	1.000	0.103	0.564	0.711
		0.201	0.201	0.635	0.666	0.213	0.441	0.232
		0.332	0.332	0.134	0.095	0.333	0.215	0.201
		0.320	0.320	0.117	0.091	0.320	0.216	0.044
		0.936	0.936	0.110	0.002	0.936	0.655	0.249
		0.110	0.110	0.207	0.226	0.111	0.104	0.041
100	0.0	0.053	0.053	0.961	1.000	0.053	0.246	0.599
		0.005	0.005	0.425	0.460	0.013	0.268	0.138
		0.178	0.178	0.088	0.050	0.178	0.171	0.129
		0.172	0.172	0.065	0.050	0.172	0.229	0.039
		0.944	0.944	0.038	0.000	0.945	0.754	0.401
		0.032	0.032	0.188	0.214	0.032	0.101	0.036
	0.2	0.225	0.225	0.995	1.000	0.239	0.469	0.919
		0.200	0.200	0.624	0.659	0.209	0.427	0.323
		0.178	0.178	0.088	0.050	0.178	0.172	0.154
		0.172	0.172	0.065	0.050	0.173	0.230	0.042
		0.942	0.942	0.039	0.000	0.944	0.794	0.274
		0.032	0.032	0.187	0.213	0.032	0.081	0.039

B.6 Simulation (c): balanced pleiotropy

Table AK. In each cell, from top to bottom are empirical type-I error/power, mean($\hat{\theta}$), SD($\hat{\theta}$), mean(SE($\hat{\theta}$)), coverage rate, MSE, when $n = 50000$, p_invalid=0.3.

m	θ	oracle	default	random	ivw	radial	mixIE-MA-DP	cML-MA-DP
30	0.0	0.059	0.059	0.051	0.050	0.058	0.059	0.039
		-0.007	-0.007	-0.003	-0.001	-0.007	0.000	0.000
		0.181	0.181	0.067	0.049	0.181	0.013	0.012
		0.173	0.173	0.062	0.048	0.173	0.013	0.012
		0.935	0.935	0.936	0.950	0.934	0.941	0.961
		0.033	0.033	0.005	0.002	0.033	0.000	0.000
	0.2	0.189	0.189	0.836	0.966	0.190	1.000	1.000
		0.186	0.186	0.196	0.199	0.187	0.199	0.197
		0.181	0.181	0.068	0.049	0.181	0.015	0.014
		0.174	0.174	0.062	0.048	0.174	0.015	0.015
		0.934	0.934	0.939	0.945	0.934	0.945	0.959
		0.033	0.033	0.005	0.002	0.033	0.000	0.000
100	0.0	0.056	0.056	0.056	0.054	0.057	0.065	0.044
		-0.003	-0.003	0.000	0.000	-0.003	0.000	0.000
		0.098	0.098	0.038	0.027	0.098	0.007	0.007
		0.095	0.095	0.036	0.027	0.095	0.007	0.007
		0.940	0.940	0.938	0.946	0.941	0.935	0.956
		0.010	0.010	0.001	0.001	0.010	0.000	0.000
	0.2	0.501	0.501	0.978	1.000	0.507	1.000	1.000
		0.187	0.187	0.199	0.199	0.188	0.199	0.199
		0.099	0.099	0.038	0.028	0.099	0.009	0.008
		0.095	0.095	0.036	0.027	0.095	0.008	0.008
		0.940	0.940	0.935	0.940	0.941	0.928	0.944
		0.010	0.010	0.001	0.001	0.010	0.000	0.000

Table AL. In each cell, from top to bottom are empirical type-I error/power, mean($\hat{\theta}$), SD($\hat{\theta}$), mean(SE($\hat{\theta}$)), coverage rate, MSE, when $n = 50000$, p_invalid=0.7.

m	θ	oracle	default	random	ivw	radial	mixIE-MA-DP	cML-MA-DP
30	0.0	0.052	0.052	0.042	0.056	0.049	0.038	0.044
		-0.018	-0.018	0.001	0.000	-0.017	0.000	0.000
		0.276	0.276	0.097	0.075	0.276	0.026	0.020
		0.268	0.268	0.094	0.074	0.268	0.030	0.020
		0.938	0.938	0.949	0.944	0.935	0.962	0.956
		0.077	0.077	0.009	0.006	0.077	0.001	0.000
	0.2	0.108	0.108	0.585	0.772	0.109	0.974	0.928
		0.177	0.177	0.201	0.200	0.180	0.198	0.175
		0.276	0.276	0.097	0.075	0.276	0.030	0.042
		0.268	0.268	0.094	0.074	0.268	0.035	0.035
		0.937	0.937	0.946	0.943	0.938	0.956	0.877
		0.077	0.077	0.009	0.006	0.077	0.001	0.002
100	0.0	0.057	0.057	0.054	0.058	0.055	0.037	0.041
		-0.008	-0.008	-0.002	-0.001	-0.008	0.000	0.000
		0.148	0.148	0.057	0.042	0.148	0.014	0.013
		0.143	0.143	0.053	0.040	0.143	0.016	0.013
		0.940	0.940	0.943	0.942	0.940	0.963	0.959
		0.022	0.022	0.003	0.002	0.022	0.000	0.000
	0.2	0.241	0.241	0.910	1.000	0.245	1.000	1.000
		0.182	0.182	0.196	0.198	0.184	0.199	0.194
		0.148	0.148	0.058	0.042	0.147	0.016	0.017
		0.144	0.144	0.053	0.041	0.144	0.018	0.017
		0.942	0.942	0.940	0.938	0.944	0.967	0.933
		0.022	0.022	0.003	0.002	0.022	0.000	0.000

Table AM. In each cell, from top to bottom are empirical type-I error/power, mean($\hat{\theta}$), SD($\hat{\theta}$), mean(SE($\hat{\theta}$)), coverage rate, MSE, when $n = 50000$, p_invalid=1.

m	θ	oracle	default	random	ivw	radial	mixIE-MA-DP	cML-MA-DP
30	0.0	0.053	0.053	0.052	0.059	0.053	0.099	0.178
		-0.015	-0.015	0.001	-0.001	-0.015	-0.007	0.001
		0.322	0.322	0.123	0.090	0.323	0.178	0.076
		0.320	0.320	0.113	0.088	0.320	0.207	0.047
		0.942	0.942	0.943	0.941	0.943	0.901	0.822
		0.104	0.104	0.015	0.008	0.104	0.032	0.006
	0.2	0.080	0.080	0.447	0.637	0.082	0.205	0.252
		0.178	0.178	0.200	0.199	0.182	0.182	0.043
		0.322	0.322	0.123	0.090	0.323	0.182	0.099
		0.320	0.320	0.114	0.088	0.320	0.212	0.052
		0.943	0.943	0.941	0.938	0.945	0.907	0.218
		0.104	0.104	0.015	0.008	0.104	0.034	0.034
100	0.0	0.053	0.053	0.060	0.065	0.052	0.020	0.135
		-0.006	-0.006	0.000	0.000	-0.006	-0.004	0.001
		0.176	0.176	0.069	0.051	0.176	0.134	0.061
		0.171	0.171	0.063	0.048	0.171	0.171	0.046
		0.943	0.943	0.940	0.935	0.944	0.980	0.865
		0.031	0.031	0.005	0.003	0.031	0.018	0.004
	0.2	0.180	0.180	0.853	0.977	0.192	0.151	0.259
		0.184	0.184	0.199	0.200	0.187	0.183	0.061
		0.176	0.176	0.069	0.051	0.175	0.137	0.083
		0.171	0.171	0.063	0.048	0.171	0.173	0.053
		0.939	0.939	0.937	0.934	0.941	0.972	0.305
		0.031	0.031	0.005	0.003	0.031	0.019	0.026

Table AN. In each cell, from top to bottom are empirical type-I error/power, mean($\hat{\theta}$), SD($\hat{\theta}$), mean(SE($\hat{\theta}$)), coverage rate, MSE, when $n = 1e + 05$, p_invalid=0.3.

m	θ	oracle	default	random	ivw	radial	mixIE-MA-DP	cML-MA-DP
30	0.0	0.046<br>0.003<br>0.177<br>0.172<br>0.938<br>0.031	0.046<br>0.003<br>0.177<br>0.172<br>0.938<br>0.031	0.052<br>0.002<br>0.065<br>0.061<br>0.936<br>0.004	0.052<br>0.001<br>0.048<br>0.047<br>0.948<br>0.002	0.044<br>0.003<br>0.177<br>0.172<br>0.938<br>0.031	0.044<br>0.000<br>0.008<br>0.009<br>0.956<br>0.000	0.031<br>0.000<br>0.008<br>0.008<br>0.969<br>0.000
	0.2	0.235<br>0.200<br>0.177<br>0.172<br>0.939<br>0.031	0.235<br>0.200<br>0.177<br>0.172<br>0.939<br>0.031	0.848<br>0.202<br>0.065<br>0.061<br>0.941<br>0.004	0.968<br>0.201<br>0.048<br>0.047<br>0.946<br>0.002	0.235<br>0.201<br>0.177<br>0.172<br>0.938<br>0.031	1.000<br>0.200<br>0.009<br>0.010<br>0.960<br>0.000	1.000<br>0.199<br>0.009<br>0.010<br>0.965<br>0.000
100	0.0	0.054<br>0.004<br>0.093<br>0.094<br>0.943<br>0.009	0.054<br>0.004<br>0.093<br>0.094<br>0.943<br>0.009	0.052<br>-0.003<br>0.038<br>0.035<br>0.940<br>0.001	0.062<br>-0.001<br>0.027<br>0.026<br>0.938<br>0.001	0.054<br>0.004<br>0.093<br>0.094<br>0.942<br>0.009	0.041<br>0.000<br>0.005<br>0.005<br>0.959<br>0.000	0.029<br>0.000<br>0.004<br>0.005<br>0.971<br>0.000
	0.2	0.574<br>0.198<br>0.094<br>0.094<br>0.945<br>0.009	0.574<br>0.198<br>0.094<br>0.094<br>0.945<br>0.009	0.974<br>0.197<br>0.038<br>0.035<br>0.942<br>0.001	1.000<br>0.199<br>0.027<br>0.026<br>0.939<br>0.001	0.579<br>0.200<br>0.094<br>0.094<br>0.944<br>0.009	1.000<br>0.200<br>0.005<br>0.005<br>0.950<br>0.000	1.000<br>0.199<br>0.005<br>0.006<br>0.952<br>0.000

Table AO. In each cell, from top to bottom are empirical type-I error/power, mean($\hat{\theta}$), SD($\hat{\theta}$), mean(SE($\hat{\theta}$)), coverage rate, MSE, when $n = 1e + 05$, p_invalid=0.7.

m	θ	oracle	default	random	ivw	radial	mixIE-MA-DP	cML-MA-DP
30	0.0	0.046	0.046	0.061	0.062	0.046	0.043	0.064
		0.001	0.001	0.003	-0.001	0.001	0.000	-0.001
		0.272	0.272	0.100	0.076	0.273	0.021	0.014
		0.266	0.266	0.094	0.073	0.266	0.020	0.014
		0.943	0.943	0.931	0.938	0.942	0.957	0.936
		0.074	0.074	0.010	0.006	0.074	0.000	0.000
	0.2	0.119	0.119	0.594	0.757	0.121	0.987	0.914
		0.198	0.198	0.203	0.200	0.200	0.196	0.173
		0.272	0.272	0.100	0.076	0.272	0.028	0.047
		0.267	0.267	0.094	0.073	0.266	0.024	0.029
		0.941	0.941	0.931	0.938	0.941	0.950	0.855
		0.074	0.074	0.010	0.006	0.074	0.001	0.003
100	0.0	0.050	0.050	0.047	0.062	0.049	0.034	0.047
		0.006	0.006	-0.002	-0.003	0.006	0.000	0.000
		0.148	0.148	0.055	0.041	0.148	0.009	0.009
		0.144	0.144	0.053	0.040	0.144	0.010	0.009
		0.947	0.947	0.951	0.938	0.947	0.966	0.953
		0.022	0.022	0.003	0.002	0.022	0.000	0.000
	0.2	0.283	0.283	0.924	0.997	0.291	1.000	1.000
		0.201	0.201	0.197	0.196	0.203	0.199	0.195
		0.149	0.149	0.055	0.041	0.148	0.011	0.012
		0.144	0.144	0.054	0.040	0.144	0.012	0.011
		0.948	0.948	0.951	0.938	0.947	0.962	0.922
		0.022	0.022	0.003	0.002	0.022	0.000	0.000

Table AP. In each cell, from top to bottom are empirical type-I error/power, mean($\hat{\theta}$), SD($\hat{\theta}$), mean(SE($\hat{\theta}$)), coverage rate, MSE, when $n = 1e + 05$, p_invalid=1.

m	θ	oracle	default	random	ivw	radial	mixIE-MA-DP	cML-MA-DP
30	0.0	0.057<br>0.004<br>0.333<br>0.320<br>0.934<br>0.111	0.057<br>0.004<br>0.333<br>0.320<br>0.934<br>0.111	0.059<br>-0.003<br>0.123<br>0.113<br>0.933<br>0.015	0.067<br>0.001<br>0.092<br>0.087<br>0.933<br>0.008	0.056<br>0.004<br>0.333<br>0.320<br>0.932<br>0.111	0.151<br>-0.004<br>0.181<br>0.189<br>0.849<br>0.033	0.236<br>-0.002<br>0.065<br>0.036<br>0.764<br>0.004
	0.2	0.096<br>0.201<br>0.332<br>0.320<br>0.935<br>0.110	0.096<br>0.201<br>0.332<br>0.320<br>0.935<br>0.110	0.429<br>0.197<br>0.123<br>0.113<br>0.933<br>0.015	0.627<br>0.201<br>0.092<br>0.087<br>0.939<br>0.008	0.100<br>0.204<br>0.332<br>0.320<br>0.934<br>0.110	0.232<br>0.165<br>0.177<br>0.188<br>0.840<br>0.033	0.239<br>0.026<br>0.079<br>0.040<br>0.113<br>0.037
100	0.0	0.054<br>0.005<br>0.178<br>0.172<br>0.944<br>0.032	0.054<br>0.005<br>0.178<br>0.172<br>0.944<br>0.032	0.050<br>-0.003<br>0.066<br>0.063<br>0.947<br>0.004	0.061<br>-0.002<br>0.049<br>0.048<br>0.939<br>0.002	0.053<br>0.005<br>0.178<br>0.172<br>0.945<br>0.032	0.095<br>0.004<br>0.139<br>0.170<br>0.905<br>0.019	0.165<br>0.000<br>0.055<br>0.035<br>0.835<br>0.003
	0.2	0.224<br>0.200<br>0.178<br>0.172<br>0.945<br>0.031	0.224<br>0.200<br>0.178<br>0.172<br>0.945<br>0.031	0.850<br>0.197<br>0.066<br>0.063<br>0.948<br>0.004	0.987<br>0.198<br>0.049<br>0.048<br>0.938<br>0.002	0.232<br>0.203<br>0.177<br>0.172<br>0.946<br>0.031	0.195<br>0.177<br>0.135<br>0.170<br>0.921<br>0.019	0.229<br>0.035<br>0.071<br>0.040<br>0.161<br>0.032

B.7 Figures for assessing the weak InSIDE assumption in simulations

In the main text, we see that in Simulation (a), where we generated $\boldsymbol{\beta}_{Xj} \sim \mathcal{U}((-0.2, -0.1) \cup (0.1, 0.2))$, MR-Egger with the default coding still performed reasonably well in terms of unbiasedness. We explained in Section 3.3 that this was a special case with $\bar{\boldsymbol{\beta}}_X = 0$. But in Simulation (b), where we generated $\boldsymbol{\beta}_{Xj} \sim \mathcal{U}((-0.1, -0.03) \cup (0.1, 0.2))$, MR-Egger with the default coding performed poorly with large biases. In both simulation set-ups, we generated SNP-exposure associations $\boldsymbol{\beta}_{Xj}$'s and direct effects α_j's independently. Although for each specific simulated dataset, the sample covariance of β_{Xj} and α_j ($j = 1, \ldots, m$) might not be exactly equal to zero, the weak InSIDE assumption says that across all simulated datasets, the average sample covariance between β_{Xj} and α_j will be (nearly) zero [1]. In this section, we present further evaluations for this weak InSIDE assumption under the default and oracle codings.

Fig. G shows the empirical distribution of the sample covariance $cov(\boldsymbol{\alpha}^*, \boldsymbol{\beta}^*_X)$ across 1000 replications for each scenario. We see that, in Simulation (a) with directional pleiotropy (Fig. GA), the weak InSIDE assumption held under both the default and oracle coding, thus MR-Egger with the default coding still performed well. However, in Simulation (b) with directional pleiotropy (Fig. GA), it is clear that the InSIDE assumption did not hold under the default coding, thus MR-Egger with the default coding gave biased causal estimates.

A. Simulation (a) with directional pleiotropy

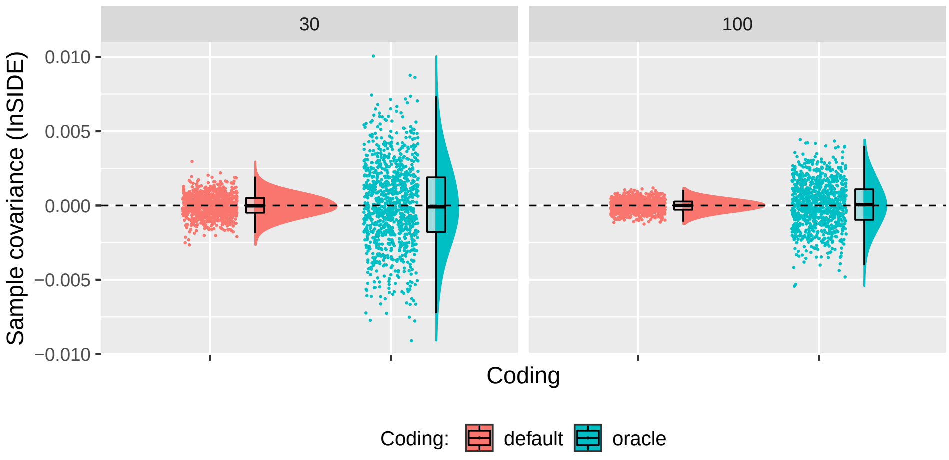

B. Simulation (b) with directional pleiotropy

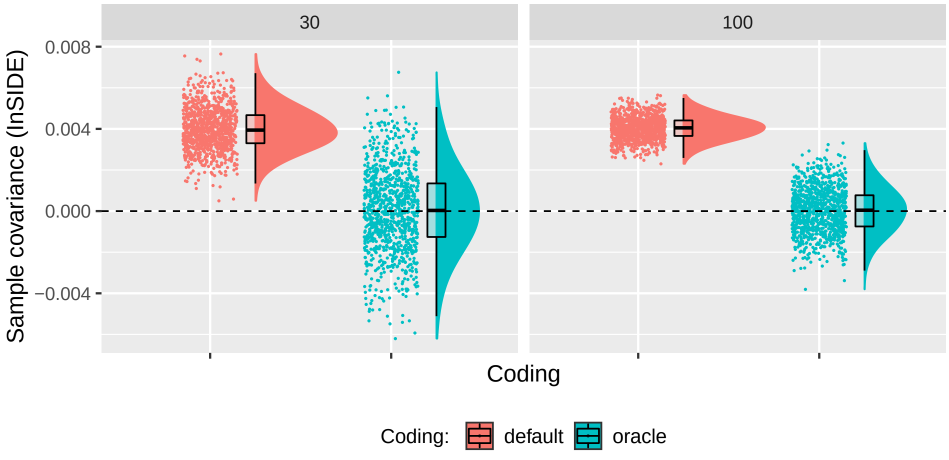

Fig G. Distributions of $cov(\boldsymbol{\alpha}^*, \boldsymbol{\beta}^*_X)$ under the default and oracle codings across 1000 replications. $n = 100\,000, \theta = 0.2$. Panel A: Simulation (a) with directional pleiotropy; Panel B: Simulation (b) with directional pleiotropy. Left: $m = 30$. Right: $m = 100$.

C Additional simulation results under the NOME assumption

Egger regression imposes the NOME assumption, which may approximately, but not exactly, hold in finite samples. However, the issue of InSIDE not holding for the default coding even if it holds for some oracle coding is not due to the violation of the NOME assumption in practice. To see this, we reran the simulations under set-up Simulation (b) with directional pleiotropy and $m = 100$, $n = 100\,000$, but we input β_{Xj}, instead of its estimate $\hat{\beta}_{Xj}$, in MR-Egger with the default and oracle codings, referred to 'defaultNOME' and 'oracleNOME' respectively. Fig. H shows the distributions of the causal estimates by the default and oracle codings with or without the NOME assumption holding. It is clear that even though we used β_{Xj} with the default coding in MR-Egger, i.e., no measurement-errors in SNP-exposure associations, we still obtained biased estimates.

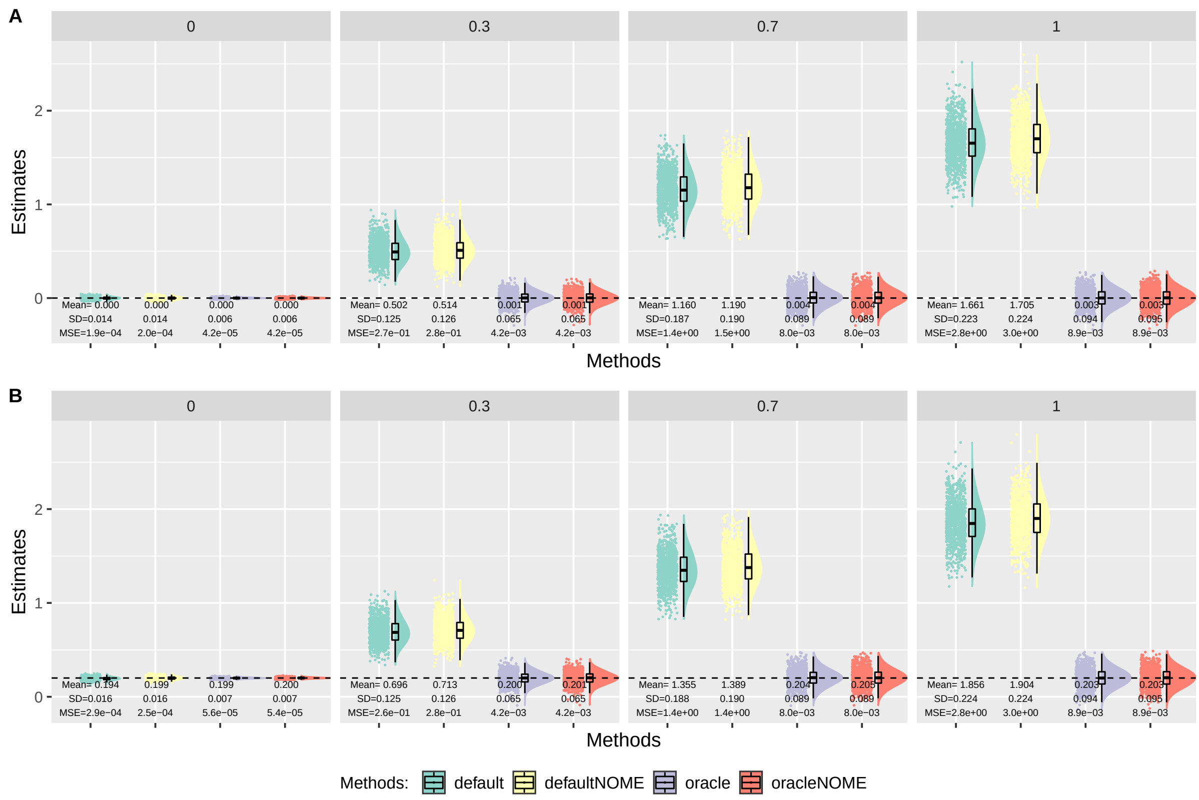

Fig H. Simulation (b) results with directional pleiotropy, $n = 100\,000, m = 100$. Empirical distributions of the estimates of the causal effect θ by the methods. Each column corresponds to 0%, 30%, 70% or 100% invalid IVs. A: $\theta = 0$. B: $\theta = 0.2$.

D Additional simulations evaluating the impact of SNP coding on violation of NOME assumption

Egger regression makes NOME assumption in SNP-exposure association, which is only an approximation to the truth when we only use strong instruments. Otherwise, it will lead to weak instrument biases in MR-Egger causal estimates. [1] proposed an I^2 ($0 < I^2 < 1$) statistic to quantify the strength of NOME violation for Egger regression, with $I^2 = 0.9$ implying a 10% relative bias in the causal estimate. In the main text, we see that using different SNP coding schemes yielded different I^2 estimates, and the default coding usually gave the smallest I^2. In this section, we conducted additional simulations to assess the impact of NOME relative to the allele coding.

We simulated data according to Eq.(1) in the main text with $G_j \sim \text{Binomial}(2, 0.3)$, and $U, \epsilon_X, \epsilon_Y \sim \mathcal{N}(0, 1)$ independently. We generated $m = 100$ IVs and their association with the exposure β_{Xj} from (i) a uniform distribution on $(-0.2, -0.1) \cup (0.1, 0.2)$; (ii)a uniform distribution on $(-0.3, -0.2) \cup (0.2, 0.3)$ and (iii) a uniform distribution on $(-0.3, -0.1) \cup (0.1, 0.3)$. We note that for all three scenarios, the means of β_{Xj} equal to zero, which are special cases where (weak) InSIDE assumption still hold when we reorientate SNPs. We generated directional pleiotropy $\alpha_j \sim \mathcal{N}(0.1, 0.1^2)$, $j = 1, \ldots, 100$. The causal effect was set to $\theta = 1$, and the sample size of GWAS was set to $n = 50\,000$ or $100\,000$.

Table AQ shows the average of the estimated causal effects and I^2 statistics across 1000 replications for each setup. First, MR-Egger with the oracle coding and IVW yielded approximately unbiased causal estimates, as expected. Second, using the default coding reduced I^2 compared to the oracle coding, leading to non-negligible finite-sample biases, though we expect that causal estimator of MR-Egger with the default coding would be unbiased if we keep increasing the sample size n since the weak InSIDE assumption held in this simulation. We also note that Radial-Egger had a smaller bias than the MR-Egger with the default coding.

Table AQ. Simulation results for different IV strengths distributions (i)-(iii). True causal effect $\theta = 1$.

n	Distributions for β_{Xj}	default		oracle		IVW	Radial
		$\widehat{I^2}$	$\hat{\theta}$	$\widehat{I^2}$	$\hat{\theta}$	$\hat{\theta}$	$\hat{\theta}$
	(i)$(-0.2, -0.1) \cup (0.1, 0.2)$	0.853	0.837	0.994	0.992	0.991	0.863
50000	(ii)$(-0.3, -0.2) \cup (0.2, 0.3)$	0.789	0.774	0.997	0.995	0.995	0.829
	(iii)$(-0.3, -0.1) \cup (0.1, 0.3)$	0.948	0.941	0.996	0.995	0.994	0.953
	(i)$(-0.2, -0.1) \cup (0.1, 0.2)$	0.922	0.916	0.997	1.000	1.005	0.943
100000	(ii)$(-0.3, -0.2) \cup (0.2, 0.3)$	0.883	0.878	0.998	1.001	1.004	0.937
	(iii)$(-0.3, -0.1) \cup (0.1, 0.3)$	0.974	0.970	0.998	1.000	1.004	0.982

E Simulations with individual-level data

Using the same simulation set-ups as in the main text, we applied several (two-sample) IV regression methods similar to two-stage least squares (TSLS) but treating direct effects of the IVs as normal random effects in stage 2 as in MR-Egger and IVW(RE), called TSLS-Egger and TSLS-IVW, with individual-level data, and each set of samples consisted of $n = 50\,000$ individuals. Specifically, in stage 1, we used the first set of samples to fit the following linear regression model:

$$\mathbf{X} = \mu_X + \mathbf{G}_x \boldsymbol{\beta}_X + \epsilon_X \tag{1}$$

where $\mathbf{X}$ is an $n-$vector of exposure and $\mathbf{G}_x$ is an $n \times m$ genotype matrix measured on the first set of samples, ϵ_X is the random error, and μ_X is the intercept, $\boldsymbol{\beta}_X$ is an $m-$vector of instrumental effect sizes on the exposure. Then in stage 2, we imputed the unobserved exposure for the second set of non-overlapping samples using the estimated parameters in model (1): $\widehat{\mathbf{X}} = \hat{\mu}_X + \mathbf{G}_y \widehat{\boldsymbol{\beta}}_X$, where $\mathbf{G}_y$ is the genotype matrix measured on the second set of samples. Then we fitted the following linear mixed model:

$$\mathbf{Y} = \mu_Y + \theta \widehat{\mathbf{X}} + \sum_{j=1}^{m} \alpha_j \mathbf{G}_{,j} + \epsilon_Y \tag{2}$$

where $\mathbf{Y}$ is an $n-$vector of outcome and $\mathbf{G}_{,j}$ is an $n-$vector of genotype of SNP G_j measured on the second set of samples, ϵ_Y is the random error, θ is the (fixed) causal effect of interest, μ_Y is the (fixed) intercept and α_j is the *random* pleiotropic effect of SNP G_j. For IVW (RE), $\alpha_j \sim \mathcal{N}(0, \sigma_\alpha^2)$ iid, and for Egger regression, $\alpha_j \sim \mathcal{N}(r, \sigma_\alpha^2)$ iid. Equivalently, we can rewrite the linear mixed model (2) for Egger regression by including $\sum_{j=1}^m \mathbf{G}_{,j}$ into the fixed effects as follows:

$$\mathbf{Y} = \mu_Y + \theta\widehat{\mathbf{X}} + r\sum_{j=1}^m \mathbf{G}_{,j} + \sum_{j=1}^m \alpha_j \mathbf{G}_{,j} + \epsilon_Y \tag{3}$$

where r is the average pleiotropic effect and the random effect becomes $\alpha_j \sim \mathcal{N}(0, \sigma_\alpha^2)$ iid.

We generated 500 independent simulated datasets. For each simulated dataset, we applied TSLS-Egger with (i) the *oracle* coding, that is, we used the simulated genotype matrix $\mathbf{G}_x$ and $\mathbf{G}_y$ under which the InSIDE assumption is satisfied; (ii) the *default* coding, that is, we flipped the coding of SNPs that we'd flipped in the MR setting, i.e. SNPs with negative *marginal* effect sizes with the exposure; for these SNPs to be flipped, we let $\mathbf{G}_{,j} = 2 - \mathbf{G}_{,j}$ in Eq (3). We also applied TSLS-IVW for comparison. And we used the R package `nlme` to fit the above linear mixed models. For $m = 500$ IVs, it took about 15 minutes to run TSLS-Egger on one simulated dataset on a core with 15GB memory. Due to the computational burden, we only considered $m = 30$ or 100 IVs in this simulation study.

As shown in Fig I and more detailed results in the following subsections, TSLS-Egger behaved similarly to MR-Egger, in which using the default coding scheme yielded biased estimates of θ except for some special scenarios.

A. Default coding

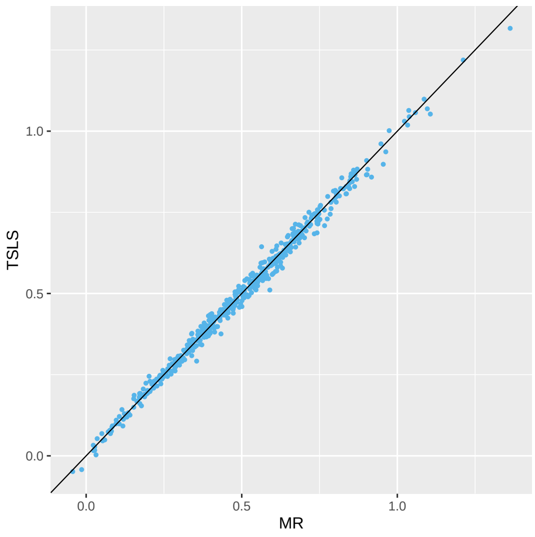

B. Oracle coding

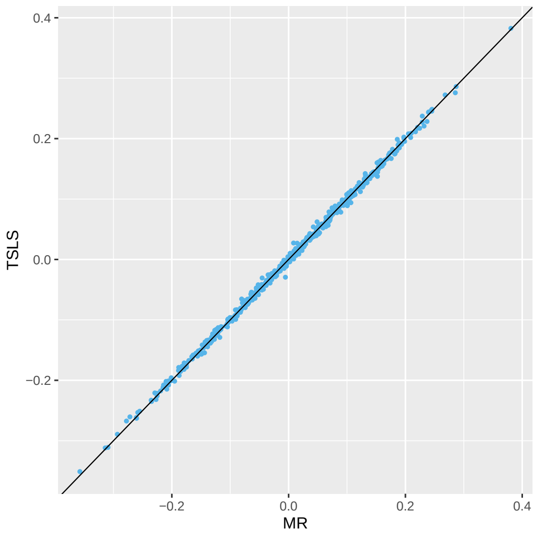

Fig I. Comparison of the causal estimates from MR-Egger and TSLS-Egger across 500 simulated datasets: Simulation (b) with directional pleiotropy, $m = 30$, $\theta = 0$ and 30% invalid IVs.

Another closely related method is PMR-Egger, which was proposed account for correlated IVs and (uncorrelated) horizontal pleiotropy [2]. Although the method was originally proposed in the context of transcriptome wide association studies (TWAS), we could be used in the context of MR (with independent IVs), under which we assessed its non-robustness to the orientation of SNPs. There are two major assumptions in PMR-Egger: $\boldsymbol{\beta}_X \sim \mathcal{N}(0, \sigma_\beta^2)$ and a constant (directional) pleiotropic effect α_j across all m IVs in Eq. (2). The first assumption leads to the requirement of the InSIDE assumption, which, along with the non-zero mean directional pleiotropy, leads to its sensitivity to the orientations of SNPs as to be shown; The second assumption is too restrictive, and may lead to biased estimation as to be shown in the current simulation set-ups.

For each simulated dataset, we applied PMR-Egger to the individual-level data. Specifically, we used the R package `PMR` (`https://github.com/yuanzhongshang/PMR`) to implement the method with the oracle and the default coding schemes described above.

As shown in the simulation results, the oracle PMR-Egger yielded unbiased estimates in the null case ($\theta = 0$) but with highly inflated type-1 errors. Its estimates were biased upward in the non-null case ($\theta = 0.2$) in all scenarios. The default PMR-Egger completely

broke down except when all IVs were valid. The problem might be (partly) due to the second assumption of an equal pleiotropic effect size in PMR-Egger, which was violated in the current simulation set-ups. Hence we considered additional simulations where the second assumption held next.

E.1 Simulation (a): directional pleiotropy

Table AR. In each cell, from top to bottom are empirical type-I error/power, mean($\hat{\theta}$), SD($\hat{\theta}$), MSE, when $n = 50000$, p_invalid=0.

m	θ	oracle-TSLS-Egger	default-TSLS-Egger	TSLS-IVW	oracle-PMR-Egger	default-PMR-Egger
30	0.0	0.038<br>0.001<br>0.011<br>0.000	0.066<br>0.004<br>0.063<br>0.004	0.048<br>0.001<br>0.011<br>0.000	0.040<br>0.001<br>0.012<br>0.000	0.066<br>0.004<br>0.067<br>0.004
	0.2	1.000<br>0.200<br>0.013<br>0.000	0.766<br>0.185<br>0.072<br>0.005	1.000<br>0.200<br>0.013<br>0.000	1.000<br>0.192<br>0.012<br>0.000	0.782<br>0.176<br>0.069<br>0.005
100	0.0	0.054<br>0.000<br>0.006<br>0.000	0.058<br>0.000<br>0.033<br>0.001	0.062<br>0.000<br>0.006<br>0.000	0.056<br>0.000<br>0.008<br>0.000	0.058<br>0.000<br>0.041<br>0.002
	0.2	1.000<br>0.200<br>0.007<br>0.000	0.998<br>0.180<br>0.038<br>0.002	1.000<br>0.200<br>0.007<br>0.000	1.000<br>0.218<br>0.008<br>0.000	0.998<br>0.195<br>0.041<br>0.002

Table AS. In each cell, from top to bottom are empirical type-I error/power, mean($\hat{\theta}$), SD($\hat{\theta}$), MSE, when $n = 50000$, p_invalid=0.3.

m	θ	oracle-TSLS-Egger	default-TSLS-Egger	TSLS-IVW	oracle-PMR-Egger	default-PMR-Egger
30	0.0	0.060<br>0.001<br>0.086<br>0.007	0.060<br>-0.028<br>0.474<br>0.225	0.072<br>0.001<br>0.095<br>0.009	0.814<br>0.001<br>0.116<br>0.013	0.998<br>-0.190<br>4.460<br>19.887
	0.2	0.660<br>0.200<br>0.086<br>0.007	0.088<br>0.151<br>0.475<br>0.227	0.608<br>0.200<br>0.096<br>0.009	0.986<br>0.226<br>0.098<br>0.010	0.996<br>0.918<br>3.960<br>16.164
100	0.0	0.050<br>0.002<br>0.049<br>0.002	0.048<br>-0.005<br>0.245<br>0.060	0.040<br>0.001<br>0.051<br>0.003	0.840<br>0.003<br>0.071<br>0.005	0.998<br>-0.305<br>5.204<br>27.118
	0.2	0.980<br>0.201<br>0.049<br>0.002	0.102<br>0.174<br>0.245<br>0.061	0.968<br>0.201<br>0.051<br>0.003	1.000<br>0.249<br>0.059<br>0.006	1.000<br>2.429<br>4.085<br>21.623

Table AT. In each cell, from top to bottom are empirical type-I error/power, mean($\hat{\theta}$), SD($\hat{\theta}$), MSE, when $n = 50000$, p_invalid=0.7.

m	θ	oracle-TSLS-Egger	default-TSLS-Egger	TSLS-IVW	oracle-PMR-Egger	default-PMR-Egger
30	0.0	0.082<br>-0.005<br>0.121<br>0.015	0.076<br>-0.043<br>0.779<br>0.607	0.074<br>-0.006<br>0.152<br>0.023	0.846<br>-0.009<br>0.196<br>0.038	1.000<br>-0.236<br>6.070<br>36.821
	0.2	0.402<br>0.194<br>0.122<br>0.015	0.076<br>0.139<br>0.780<br>0.610	0.326<br>0.193<br>0.152<br>0.023	0.966<br>0.250<br>0.155<br>0.026	1.000<br>0.691<br>5.635<br>31.929
100	0.0	0.050<br>-0.004<br>0.065<br>0.004	0.038<br>-0.011<br>0.366<br>0.134	0.048<br>-0.006<br>0.077<br>0.006	0.882<br>-0.007<br>0.104<br>0.011	1.000<br>0.062<br>6.582<br>43.238
	0.2	0.870<br>0.195<br>0.065<br>0.004	0.064<br>0.170<br>0.366<br>0.134	0.698<br>0.193<br>0.077<br>0.006	0.998<br>0.261<br>0.085<br>0.011	1.000<br>2.036<br>5.875<br>37.815

Table AU. In each cell, from top to bottom are empirical type-I error/power, mean($\hat{\theta}$), SD($\hat{\theta}$), MSE, when $n = 50000$, p_invalid=1.

m	θ	oracle-TSLS-Egger	default-TSLS-Egger	TSLS-IVW	oracle-PMR-Egger	default-PMR-Egger
30	0.0	0.074<br>-0.002<br>0.126<br>0.016	0.072<br>-0.065<br>0.901<br>0.814	0.078<br>-0.003<br>0.181<br>0.033	0.856<br>-0.005<br>0.208<br>0.043	1.000<br>-0.551<br>6.680<br>44.840
	0.2	0.400<br>0.197<br>0.126<br>0.016	0.082<br>0.115<br>0.901<br>0.817	0.262<br>0.196<br>0.181<br>0.033	0.962<br>0.259<br>0.161<br>0.029	1.000<br>0.542<br>6.290<br>39.606
100	0.0	0.056<br>-0.003<br>0.067<br>0.004	0.040<br>-0.029<br>0.438<br>0.193	0.046<br>-0.006<br>0.089<br>0.008	0.872<br>-0.005<br>0.106<br>0.011	0.998<br>-0.694<br>7.000<br>49.379
	0.2	0.850<br>0.196<br>0.067<br>0.005	0.064<br>0.151<br>0.439<br>0.194	0.562<br>0.193<br>0.089<br>0.008	1.000<br>0.260<br>0.087<br>0.011	0.996<br>1.805<br>6.481<br>44.492

E.2 Simulation (a): balanced pleiotropy

Table AV. In each cell, from top to bottom are empirical type-I error/power, mean($\hat{\theta}$), SD($\hat{\theta}$), MSE, when $n = 50000$, p_invalid=0.3.

m	θ	oracle-TSLS-Egger	default-TSLS-Egger	TSLS-IVW	oracle-PMR-Egger	default-PMR-Egger
30	0.0	0.072<br>0.002<br>0.067<br>0.005	0.068<br>-0.015<br>0.336<br>0.113	0.072<br>0.002<br>0.066<br>0.004	0.782<br>0.002<br>0.082<br>0.007	0.994<br>-0.165<br>3.197<br>10.225
30	0.2	0.848<br>0.201<br>0.068<br>0.005	0.114<br>0.163<br>0.337<br>0.115	0.840<br>0.201<br>0.067<br>0.004	0.996<br>0.212<br>0.072<br>0.005	0.982<br>0.867<br>2.659<br>7.500
100	0.0	0.052<br>0.000<br>0.036<br>0.001	0.052<br>-0.006<br>0.177<br>0.031	0.048<br>0.000<br>0.036<br>0.001	0.754<br>-0.001<br>0.049<br>0.002	1.000<br>-0.226<br>3.876<br>15.041
100	0.2	1.000<br>0.199<br>0.036<br>0.001	0.166<br>0.174<br>0.177<br>0.032	1.000<br>0.199<br>0.036<br>0.001	1.000<br>0.234<br>0.042<br>0.003	0.996<br>2.106<br>2.586<br>10.306

Table AW. In each cell, from top to bottom are empirical type-I error/power, mean($\hat{\theta}$), SD($\hat{\theta}$), MSE, when $n = 50000$, p_invalid=0.7.

m	θ	oracle-TSLS-Egger	default-TSLS-Egger	TSLS-IVW	oracle-PMR-Egger	default-PMR-Egger
30	0.0	0.078<br>-0.004<br>0.107<br>0.011	0.064<br>-0.015<br>0.557<br>0.310	0.076<br>-0.003<br>0.105<br>0.011	0.856<br>-0.006<br>0.158<br>0.025	1.000<br>-0.370<br>4.758<br>22.732
30	0.2	0.502<br>0.195<br>0.107<br>0.012	0.094<br>0.166<br>0.559<br>0.313	0.510<br>0.196<br>0.105<br>0.011	0.964<br>0.236<br>0.129<br>0.018	0.998<br>0.857<br>4.283<br>18.736
100	0.0	0.048<br>-0.005<br>0.055<br>0.003	0.064<br>-0.001<br>0.278<br>0.077	0.048<br>-0.005<br>0.054<br>0.003	0.846<br>-0.007<br>0.085<br>0.007	1.000<br>0.163<br>5.482<br>30.023
100	0.2	0.948<br>0.195<br>0.055<br>0.003	0.104<br>0.179<br>0.279<br>0.078	0.946<br>0.195<br>0.054<br>0.003	1.000<br>0.254<br>0.071<br>0.008	1.000<br>2.342<br>4.454<br>24.387

Table AX. In each cell, from top to bottom are empirical type-I error/power, mean($\hat{\theta}$), SD($\hat{\theta}$), MSE, when $n = 50000$, p_invalid=1.

m	θ	oracle-TSLS-Egger	default-TSLS-Egger	TSLS-IVW	oracle-PMR-Egger	default-PMR-Egger
30	0.0	0.074<br>-0.002<br>0.126<br>0.016	0.066<br>-0.035<br>0.648<br>0.420	0.070<br>0.000<br>0.122<br>0.015	0.856<br>-0.005<br>0.215<br>0.046	1.000<br>-0.071<br>5.423<br>29.350
	0.2	0.400<br>0.197<br>0.126<br>0.016	0.082<br>0.146<br>0.647<br>0.421	0.400<br>0.199<br>0.122<br>0.015	0.960<br>0.265<br>0.165<br>0.031	0.998<br>0.597<br>4.981<br>24.919
100	0.0	0.056<br>-0.003<br>0.067<br>0.004	0.046<br>-0.003<br>0.327<br>0.106	0.054<br>-0.003<br>0.067<br>0.004	0.870<br>-0.005<br>0.115<br>0.013	0.998<br>-0.089<br>6.068<br>36.758
	0.2	0.850<br>0.196<br>0.067<br>0.005	0.088<br>0.177<br>0.327<br>0.108	0.846<br>0.196<br>0.067<br>0.004	1.000<br>0.278<br>0.093<br>0.015	0.998<br>2.273<br>5.188<br>31.155

E.3 Simulation (b): directional pleiotropy

Table AY. In each cell, from top to bottom are empirical type-I error/power, mean($\hat{\theta}$), SD($\hat{\theta}$), MSE, when $n = 50000$, p_invalid=0.

m	θ	oracle-TSLS-Egger	default-TSLS-Egger	TSLS-IVW	oracle-PMR-Egger	default-PMR-Egger
30	0.0	0.040<br>0.001<br>0.016<br>0.000	0.050<br>0.000<br>0.035<br>0.001	0.034<br>0.001<br>0.014<br>0.000	0.040<br>0.001<br>0.017<br>0.000	0.054<br>0.000<br>0.037<br>0.001
	0.2	1.000<br>0.199<br>0.018<br>0.000	0.992<br>0.193<br>0.041<br>0.002	1.000<br>0.200<br>0.016<br>0.000	1.000<br>0.188<br>0.017<br>0.000	0.994<br>0.182<br>0.038<br>0.002
100	0.0	0.056<br>0.000<br>0.009<br>0.000	0.050<br>0.000<br>0.019<br>0.000	0.052<br>0.000<br>0.008<br>0.000	0.056<br>0.000<br>0.010<br>0.000	0.050<br>0.001<br>0.022<br>0.000
	0.2	1.000<br>0.199<br>0.010<br>0.000	1.000<br>0.193<br>0.022<br>0.001	1.000<br>0.199<br>0.009<br>0.000	1.000<br>0.206<br>0.011<br>0.000	1.000<br>0.199<br>0.023<br>0.001

Table AZ. In each cell, from top to bottom are empirical type-I error/power, mean($\hat{\theta}$), SD($\hat{\theta}$), MSE, when $n = 50000$, p_invalid=0.3.

m	θ	oracle-TSLS-Egger	default-TSLS-Egger	TSLS-IVW	oracle-PMR-Egger	default-PMR-Egger
30	0.0	0.052<br>0.002<br>0.120<br>0.014	0.458<br>0.484<br>0.223<br>0.284	0.206<br>0.113<br>0.106<br>0.024	0.834<br>0.004<br>0.236<br>0.056	0.998<br>2.605<br>1.154<br>8.116
	0.2	0.430<br>0.200<br>0.120<br>0.014	0.782<br>0.675<br>0.224<br>0.276	0.814<br>0.312<br>0.106<br>0.024	0.940<br>0.274<br>0.172<br>0.035	1.000<br>2.082<br>0.823<br>4.218
100	0.0	0.066<br>0.003<br>0.069<br>0.005	0.972<br>0.496<br>0.124<br>0.261	0.518<br>0.119<br>0.057<br>0.018	0.854<br>0.004<br>0.128<br>0.016	1.000<br>2.870<br>0.652<br>8.660
	0.2	0.844<br>0.201<br>0.069<br>0.005	1.000<br>0.689<br>0.124<br>0.254	1.000<br>0.318<br>0.057<br>0.017	0.996<br>0.286<br>0.099<br>0.017	1.000<br>2.236<br>0.496<br>4.390

Table BA. In each cell, from top to bottom are empirical type-I error/power, mean($\hat{\theta}$), SD($\hat{\theta}$), MSE, when $n = 50000$, p_invalid=0.7.

m	θ	oracle-TSLS-Egger	default-TSLS-Egger	TSLS-IVW	oracle-PMR-Egger	default-PMR-Egger
30	0.0	0.076<br>-0.009<br>0.173<br>0.030	0.900<br>1.195<br>0.347<br>1.548	0.402<br>0.261<br>0.164<br>0.095	0.926<br>-0.041<br>0.894<br>0.799	1.000<br>3.747<br>0.776<br>14.641
	0.2	0.256<br>0.189<br>0.174<br>0.030	0.970<br>1.388<br>0.348<br>1.531	0.774<br>0.460<br>0.165<br>0.095	0.956<br>0.369<br>0.382<br>0.174	1.000<br>3.237<br>0.659<br>9.658
100	0.0	0.066<br>-0.005<br>0.091<br>0.008	1.000<br>1.157<br>0.187<br>1.373	0.858<br>0.268<br>0.083<br>0.079	0.892<br>-0.005<br>0.389<br>0.151	1.000<br>3.807<br>0.439<br>14.683
	0.2	0.594<br>0.194<br>0.091<br>0.008	1.000<br>1.350<br>0.187<br>1.357	1.000<br>0.466<br>0.083<br>0.078	0.992<br>0.374<br>0.176<br>0.061	1.000<br>3.285<br>0.371<br>9.656

Table BB. In each cell, from top to bottom are empirical type-I error/power, mean($\hat{\theta}$), SD($\hat{\theta}$), MSE, when $n = 50000$, p_invalid=1.

m	θ	oracle-TSLS-Egger	default-TSLS-Egger	TSLS-IVW	oracle-PMR-Egger	default-PMR-Egger
30	0.0	0.082<br>-0.002<br>0.176<br>0.031	0.992<br>1.689<br>0.404<br>3.016	0.524<br>0.379<br>0.185<br>0.178	0.914<br>-0.006<br>1.219<br>1.484	1.000<br>3.987<br>0.702<br>16.386
	0.2	0.246<br>0.196<br>0.176<br>0.031	0.998<br>1.881<br>0.405<br>2.991	0.836<br>0.578<br>0.185<br>0.177	0.952<br>0.423<br>0.489<br>0.288	1.000<br>3.550<br>0.613<br>11.595
100	0.0	0.072<br>-0.005<br>0.093<br>0.009	1.000<br>1.659<br>0.216<br>2.798	0.962<br>0.385<br>0.094<br>0.157	0.902<br>-0.039<br>0.723<br>0.523	1.000<br>3.935<br>0.377<br>15.623
	0.2	0.556<br>0.194<br>0.093<br>0.009	1.000<br>1.851<br>0.217<br>2.774	1.000<br>0.584<br>0.094<br>0.156	0.984<br>0.398<br>0.192<br>0.076	1.000<br>3.516<br>0.325<br>11.104

E.4 Simulation (b): balanced pleiotropy

Table BC. In each cell, from top to bottom are empirical type-I error/power, mean($\hat{\theta}$), SD($\hat{\theta}$), MSE, when $n = 50000$, p_invalid=0.3.

m	θ	oracle-TSLS-Egger	default-TSLS-Egger	TSLS-IVW	oracle-PMR-Egger	default-PMR-Egger
30	0.0	0.076<br>0.002<br>0.094<br>0.009	0.062<br>-0.008<br>0.197<br>0.039	0.054<br>0.000<br>0.081<br>0.007	0.822<br>0.002<br>0.135<br>0.018	0.912<br>-0.009<br>2.009<br>4.028
	0.2	0.616<br>0.200<br>0.095<br>0.009	0.188<br>0.183<br>0.198<br>0.040	0.698<br>0.199<br>0.082<br>0.007	0.972<br>0.230<br>0.112<br>0.013	0.928<br>0.687<br>1.282<br>1.878
100	0.0	0.052<br>-0.001<br>0.051<br>0.003	0.062<br>0.000<br>0.111<br>0.012	0.064<br>0.000<br>0.044<br>0.002	0.750<br>-0.001<br>0.076<br>0.006	0.954<br>-0.041<br>2.198<br>4.822
	0.2	0.960<br>0.198<br>0.051<br>0.003	0.436<br>0.193<br>0.112<br>0.013	0.996<br>0.199<br>0.044<br>0.002	1.000<br>0.246<br>0.063<br>0.006	0.982<br>1.055<br>0.867<br>1.481

Table BD. In each cell, from top to bottom are empirical type-I error/power, mean($\hat{\theta}$), SD($\hat{\theta}$), MSE, when $n = 50000$, p_invalid=0.7.

m	θ	oracle-TSLS-Egger	default-TSLS-Egger	TSLS-IVW	oracle-PMR-Egger	default-PMR-Egger
30	0.0	0.070<br>-0.006<br>0.150<br>0.022	0.070<br>0.025<br>0.309<br>0.096	0.060<br>0.000<br>0.127<br>0.016	0.880<br>-0.016<br>0.472<br>0.223	0.998<br>0.435<br>4.170<br>17.541
	0.2	0.320<br>0.192<br>0.150<br>0.023	0.128<br>0.217<br>0.310<br>0.096	0.400<br>0.199<br>0.128<br>0.016	0.926<br>0.308<br>0.256<br>0.077	0.998<br>1.477<br>3.156<br>11.573
100	0.0	0.058<br>-0.006<br>0.076<br>0.006	0.068<br>0.000<br>0.172<br>0.029	0.058<br>-0.005<br>0.069<br>0.005	0.872<br>-0.014<br>0.174<br>0.031	1.000<br>0.049<br>4.926<br>24.215
	0.2	0.710<br>0.192<br>0.076<br>0.006	0.222<br>0.192<br>0.172<br>0.030	0.812<br>0.194<br>0.069<br>0.005	0.998<br>0.320<br>0.126<br>0.030	1.000<br>2.862<br>2.873<br>15.324

Table BE. In each cell, from top to bottom are empirical type-I error/power, mean($\hat{\theta}$), SD($\hat{\theta}$), MSE, when $n = 50000$, p_invalid=1.

m	θ	oracle-TSLS-Egger	default-TSLS-Egger	TSLS-IVW	oracle-PMR-Egger	default-PMR-Egger
30	0.0	0.082<br>-0.002<br>0.176<br>0.031	0.068<br>0.020<br>0.368<br>0.136	0.076<br>0.002<br>0.151<br>0.023	0.912<br>-0.006<br>1.254<br>1.570	1.000<br>0.318<br>5.148<br>26.551
	0.2	0.246<br>0.196<br>0.176<br>0.031	0.100<br>0.212<br>0.368<br>0.136	0.308<br>0.200<br>0.151<br>0.023	0.950<br>0.436<br>0.504<br>0.309	0.998<br>1.884<br>4.092<br>19.549
100	0.0	0.072<br>-0.005<br>0.093<br>0.009	0.056<br>0.005<br>0.199<br>0.040	0.070<br>-0.002<br>0.082<br>0.007	0.902<br>-0.041<br>0.781<br>0.611	1.000<br>0.126<br>5.777<br>33.326
	0.2	0.556<br>0.194<br>0.093<br>0.009	0.174<br>0.198<br>0.200<br>0.040	0.684<br>0.197<br>0.083<br>0.007	0.986<br>0.432<br>0.208<br>0.097	1.000<br>3.232<br>3.895<br>24.334

F Additional simulations for PMR-Egger

We conducted more simulations to study PMR-Egger's performance with various orientations of SNPs when its second assumption of an equal pleiotropic effect size held. All set-ups were the same as that in the previous section except that we set the directional pleiotropy as a constant, $\boldsymbol{\alpha}_{yg} = 0.01$, for all the K invalid IVs . We applied PMR-Egger and TSLS-Egger to the generated individual-level data.

As shown in the simulation results, when the pleiotropic effect sizes were the same across all invalid IVs, both the default and oracle PMR-Egger performed much more

stably than in the previous set-ups, and also more similarly to their TSLS counterparts. Specifically, the oracle-PMR-Egger performed similarly to the oracle-TSLS-Egger in the null case, yielding the unbiased estimates and well-calibrated type-I errors. But in the non-null case, the oracle-PMR-Egger still produced slightly biased estimates. On the other hand, the default-PMR-Egger also yielded similar estimates to those of the default-TSLS-Egger, which gave almost unbiased estimates in Simulation (a), but inflated type-I errors. In Simulation (b), both the default-PMR-Egger and default-TSLS-Egger gave biased estimates of θ.

F.1 Simulation (a): directional pleiotropy

Table BF. In each cell, from top to bottom are empirical type-I error/power, mean($\hat{\theta}$), SD($\hat{\theta}$), MSE, when $n = 50000$, p_invalid=0.3.

m	θ	oracle-TSLS-Egger	default-TSLS-Egger	TSLS-IVW	oracle-PMR-Egger	default-PMR-Egger
30	0.0	0.050<br>0.001<br>0.013<br>0.000	0.092<br>0.003<br>0.071<br>0.005	0.056<br>0.000<br>0.013<br>0.000	0.068<br>0.001<br>0.014<br>0.000	0.114<br>0.004<br>0.078<br>0.006
	0.2	1.000<br>0.200<br>0.014<br>0.000	0.732<br>0.184<br>0.079<br>0.007	1.000<br>0.199<br>0.015<br>0.000	1.000<br>0.192<br>0.014<br>0.000	0.760<br>0.180<br>0.078<br>0.006
100	0.0	0.056<br>0.001<br>0.007<br>0.000	0.068<br>0.000<br>0.037<br>0.001	0.070<br>0.000<br>0.007<br>0.000	0.060<br>0.001<br>0.009<br>0.000	0.102<br>0.000<br>0.047<br>0.002
	0.2	1.000<br>0.200<br>0.008<br>0.000	0.994<br>0.180<br>0.041<br>0.002	1.000<br>0.200<br>0.008<br>0.000	1.000<br>0.218<br>0.008<br>0.000	0.998<br>0.200<br>0.046<br>0.002

Table BG. In each cell, from top to bottom are empirical type-I error/power, mean($\hat{\theta}$), SD($\hat{\theta}$), MSE, when $n = 50000$, p_invalid=0.7.

m	θ	oracle-TSLS-Egger	default-TSLS-Egger	TSLS-IVW	oracle-PMR-Egger	default-PMR-Egger
30	0.0	0.054<br>0.000<br>0.013<br>0.000	0.058<br>0.000<br>0.081<br>0.007	0.062<br>0.000<br>0.015<br>0.000	0.062<br>0.001<br>0.014<br>0.000	0.138<br>0.000<br>0.093<br>0.009
	0.2	1.000<br>0.200<br>0.014<br>0.000	0.636<br>0.181<br>0.088<br>0.008	1.000<br>0.199<br>0.017<br>0.000	1.000<br>0.192<br>0.013<br>0.000	0.722<br>0.183<br>0.089<br>0.008
100	0.0	0.062<br>0.000<br>0.007<br>0.000	0.052<br>0.000<br>0.042<br>0.002	0.062<br>0.000<br>0.009<br>0.000	0.072<br>0.000<br>0.009<br>0.000	0.156<br>0.000<br>0.055<br>0.003
	0.2	1.000<br>0.200<br>0.008<br>0.000	0.978<br>0.180<br>0.045<br>0.002	1.000<br>0.200<br>0.009<br>0.000	1.000<br>0.218<br>0.009<br>0.000	0.992<br>0.208<br>0.052<br>0.003

Table BH. In each cell, from top to bottom are empirical type-I error/power, mean($\hat{\theta}$), SD($\hat{\theta}$), MSE, when $n = 50000$, p_invalid=1.

m	θ	oracle-TSLS-Egger	default-TSLS-Egger	TSLS-IVW	oracle-PMR-Egger	default-PMR-Egger
30	0.0	0.038<br>0.001<br>0.011<br>0.000	0.056<br>0.000<br>0.088<br>0.008	0.050<br>0.000<br>0.017<br>0.000	0.042<br>0.001<br>0.012<br>0.000	0.188<br>0.000<br>0.105<br>0.011
	0.2	1.000<br>0.200<br>0.013<br>0.000	0.558<br>0.181<br>0.094<br>0.009	1.000<br>0.199<br>0.018<br>0.000	1.000<br>0.192<br>0.012<br>0.000	0.718<br>0.188<br>0.098<br>0.010
100	0.0	0.054<br>0.000<br>0.006<br>0.000	0.044<br>-0.001<br>0.046<br>0.002	0.064<br>0.000<br>0.009<br>0.000	0.056<br>0.000<br>0.008<br>0.000	0.198<br>-0.001<br>0.062<br>0.004
	0.2	1.000<br>0.200<br>0.007<br>0.000	0.946<br>0.179<br>0.049<br>0.003	1.000<br>0.199<br>0.010<br>0.000	1.000<br>0.218<br>0.008<br>0.000	0.992<br>0.212<br>0.058<br>0.003

F.2 Simulation (b): directional pleiotropy

Table BI. In each cell, from top to bottom are empirical type-I error/power, mean($\hat{\theta}$), SD($\hat{\theta}$), MSE, when $n = 50000$, p_invalid=0.3.

m	θ	oracle-TSLS-Egger	default-TSLS-Egger	TSLS-IVW	oracle-PMR-Egger	default-PMR-Egger
30	0.0	0.071<br>0.001<br>0.018<br>0.000	0.234<br>0.050<br>0.038<br>0.004	0.123<br>0.012<br>0.015<br>0.000	0.084<br>0.001<br>0.019<br>0.000	0.270<br>0.053<br>0.040<br>0.004
	0.2	1.000<br>0.200<br>0.020<br>0.000	1.000<br>0.242<br>0.043<br>0.004	1.000<br>0.211<br>0.017<br>0.000	1.000<br>0.189<br>0.019<br>0.000	1.000<br>0.230<br>0.040<br>0.003
100	0.0	0.065<br>0.001<br>0.010<br>0.000	0.688<br>0.050<br>0.020<br>0.003	0.294<br>0.012<br>0.009<br>0.000	0.076<br>0.001<br>0.011<br>0.000	0.738<br>0.058<br>0.023<br>0.004
	0.2	1.000<br>0.199<br>0.011<br>0.000	1.000<br>0.242<br>0.023<br>0.002	1.000<br>0.211<br>0.010<br>0.000	1.000<br>0.206<br>0.011<br>0.000	1.000<br>0.252<br>0.024<br>0.003

Table BJ. In each cell, from top to bottom are empirical type-I error/power, mean($\hat{\theta}$), SD($\hat{\theta}$), MSE, when $n = 50000$, p_invalid=0.7.

m	θ	oracle-TSLS-Egger	default-TSLS-Egger	TSLS-IVW	oracle-PMR-Egger	default-PMR-Egger
30	0.0	0.066<br>0.001<br>0.018<br>0.000	0.848<br>0.117<br>0.039<br>0.015	0.352<br>0.027<br>0.016<br>0.001	0.072<br>0.001<br>0.019<br>0.000	0.878<br>0.124<br>0.041<br>0.017
	0.2	1.000<br>0.199<br>0.020<br>0.000	1.000<br>0.310<br>0.044<br>0.014	1.000<br>0.226<br>0.018<br>0.001	1.000<br>0.188<br>0.019<br>0.000	1.000<br>0.295<br>0.041<br>0.011
100	0.0	0.076<br>0.001<br>0.010<br>0.000	1.000<br>0.116<br>0.021<br>0.014	0.804<br>0.028<br>0.009<br>0.001	0.088<br>0.001<br>0.012<br>0.000	1.000<br>0.136<br>0.024<br>0.019
	0.2	1.000<br>0.199<br>0.011<br>0.000	1.000<br>0.308<br>0.023<br>0.012	1.000<br>0.226<br>0.010<br>0.001	1.000<br>0.206<br>0.012<br>0.000	1.000<br>0.321<br>0.024<br>0.015

Table BK. In each cell, from top to bottom are empirical type-I error/power, mean($\hat{\theta}$), SD($\hat{\theta}$), MSE, when $n = 50000$, p_invalid=1.

m	θ	oracle-TSLS-Egger	default-TSLS-Egger	TSLS-IVW	oracle-PMR-Egger	default-PMR-Egger
30	0.0	0.040<br>0.001<br>0.016<br>0.000	0.986<br>0.167<br>0.038<br>0.029	0.556<br>0.039<br>0.016<br>0.002	0.040<br>0.001<br>0.017<br>0.000	0.996<br>0.177<br>0.040<br>0.033
	0.2	1.000<br>0.199<br>0.018<br>0.000	1.000<br>0.360<br>0.043<br>0.027	1.000<br>0.237<br>0.018<br>0.002	1.000<br>0.188<br>0.017<br>0.000	1.000<br>0.341<br>0.041<br>0.022
100	0.0	0.056<br>0.000<br>0.009<br>0.000	1.000<br>0.166<br>0.021<br>0.028	0.968<br>0.039<br>0.009<br>0.002	0.056<br>0.000<br>0.010<br>0.000	1.000<br>0.194<br>0.025<br>0.038
	0.2	1.000<br>0.199<br>0.010<br>0.000	1.000<br>0.358<br>0.024<br>0.025	1.000<br>0.238<br>0.010<br>0.002	1.000<br>0.205<br>0.011<br>0.000	1.000<br>0.372<br>0.024<br>0.030

G Simulations with irrelevant IVs

We conducted simulation studies to assess the robustness of MR-Egger to the presence of irrelevant IVs. Specifically, we followed the simulation set-ups in the main text:

$$X = \sum_{j=1}^{m+m_0} \beta_{Xj} G_j + U + \epsilon_X,$$
$$Y = \sum_{j=1}^{m+m_0} \alpha_j G_j + \theta X + U + \epsilon_Y, \tag{4}$$

with $G_j \sim$ Binomial$(2, 0.3)$, and $U, \epsilon_X, \epsilon_Y \sim \mathcal{N}(0, 1)$ independently. The number of relevant IVs, m, was set to 100, and the number of irrelevant IVs, m_0, was set to 10 or 30.

For the m relevant IVs, we followed the Simulation Set-ups (a) and (b), where β_{Xj} was generated from (a) a uniform distribution on $(-0.2, -0.1) \cup (0.1, 0.2)$ and (b) a uniform distribution on $(-0.1, -0.03) \cup (0.1, 0.2)$, respectively. And similarly, we considered 0%, 30% or 70% invalid IVs with directional pleiotropy $\alpha_j \sim \mathcal{N}(0.1, 0.1^2)$.

For the m_0 irrelevant IVs, $\beta_{Xj} = 0$. At the same time, we also considered 0% or 50% of them having direct effect on Y. Specifically, we generated the direct effect $\alpha_j \sim \mathcal{N}(0.1, 0.1^2)$.

The causal effect θ was set to 0 or 0.2. The summary data for genetic associations were calculated for the exposure and the outcome on non-overlapping samples of individuals, each consisting of $n = 50\,000$ individuals.

As shown below, in Simulation (a), where the special case $\sum_{j=1}^{m} \beta_{Xj} = 0$ held, both the oracle and default MR-Egger yielded unbiased estimates in the presence of a few irrelevant IVs. However in Simulation (b) with directional pleiotropy, none of the special cases with $\sum_{j=1}^{m} \beta_{Xj} = 0$, or $\sum_{j=1}^{m} \alpha_j = \sum_{j=1}^{m+m_0} \alpha_j = 0$ held, then MR-Egger with the oracle coding gave biased estimates, and MR-Egger with the default coding gave the most biased results among all methods being compared. We can also see that mixIE (with default coding) also performed much more robustly to irrelevant IVs than MR-Egger – it gave slightly biased estimates when the proportion of invalid IVs was high. Lastly, as expected, MR-cML performed most robustly to irrelevant IVs in both simulation set-ups.

G.1 Simulation (a): directional pleiotropy

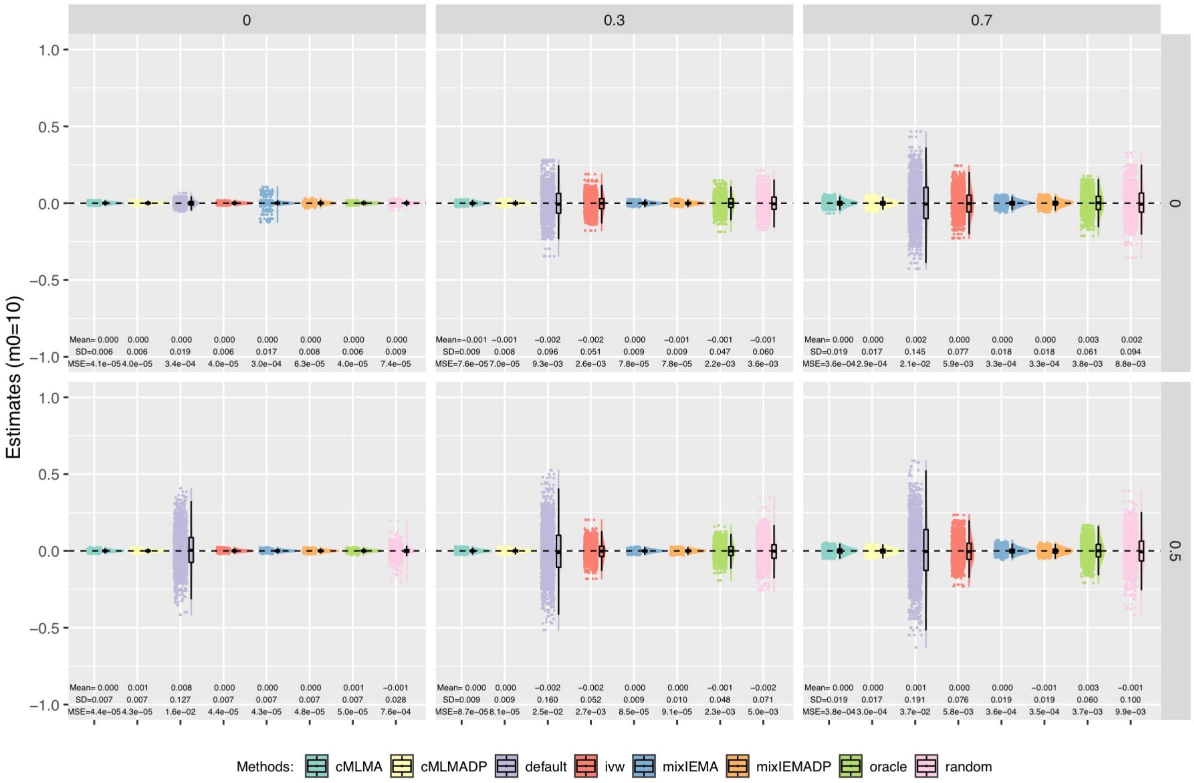

Fig J. Simulation (a) results with directional pleiotropy, $n = 50\,000, m_0 = 10, \theta = 0$. Empirical distributions of the estimates of the causal effect θ by the methods. Each column corresponds to 0%, 30% or 70% invalid IVs. Top: Irrelevant IVs do not have direct effect. Bottom: 50% of irrelevant IVs have direct effect.

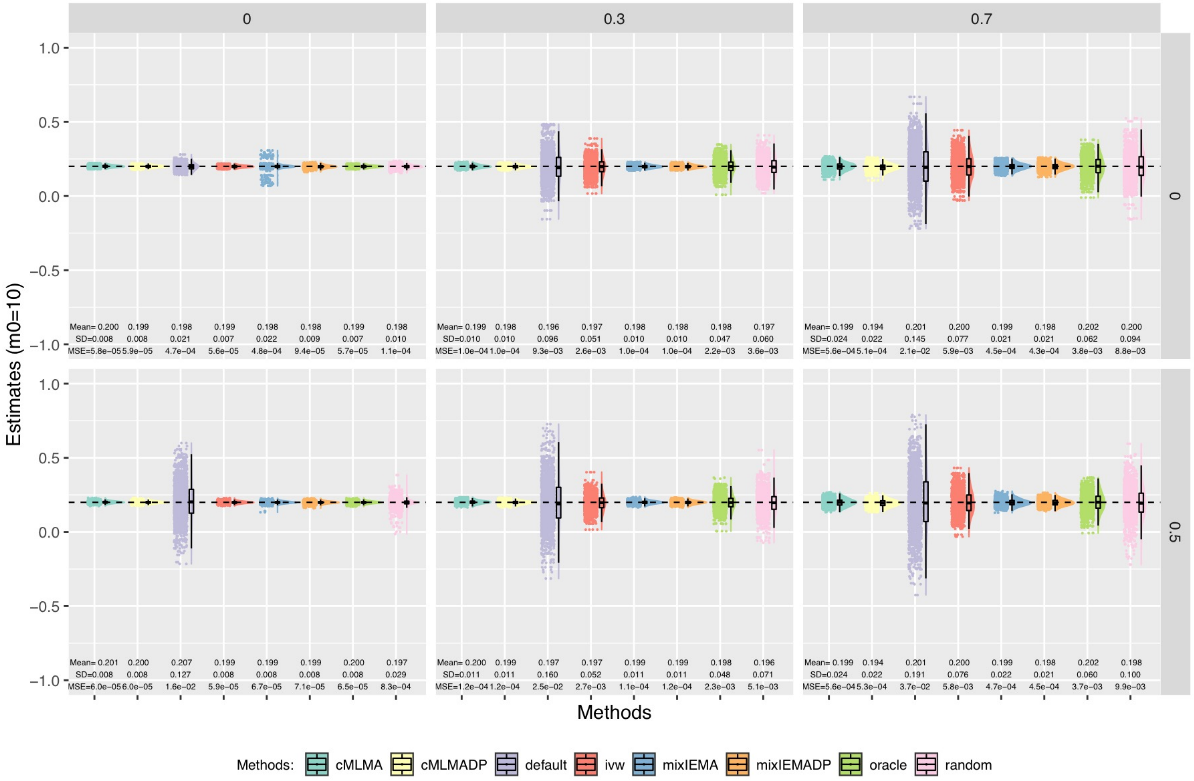

Fig K. Simulation (a) results with directional pleiotropy, $n = 50\,000, m_0 = 10, \theta = 0.2$. Empirical distributions of the estimates of the causal effect θ by the methods. Each column corresponds to 0%, 30% or 70% invalid IVs. Top: Irrelevant IVs do not have direct effect. Bottom: 50% of irrelevant IVs have direct effect.

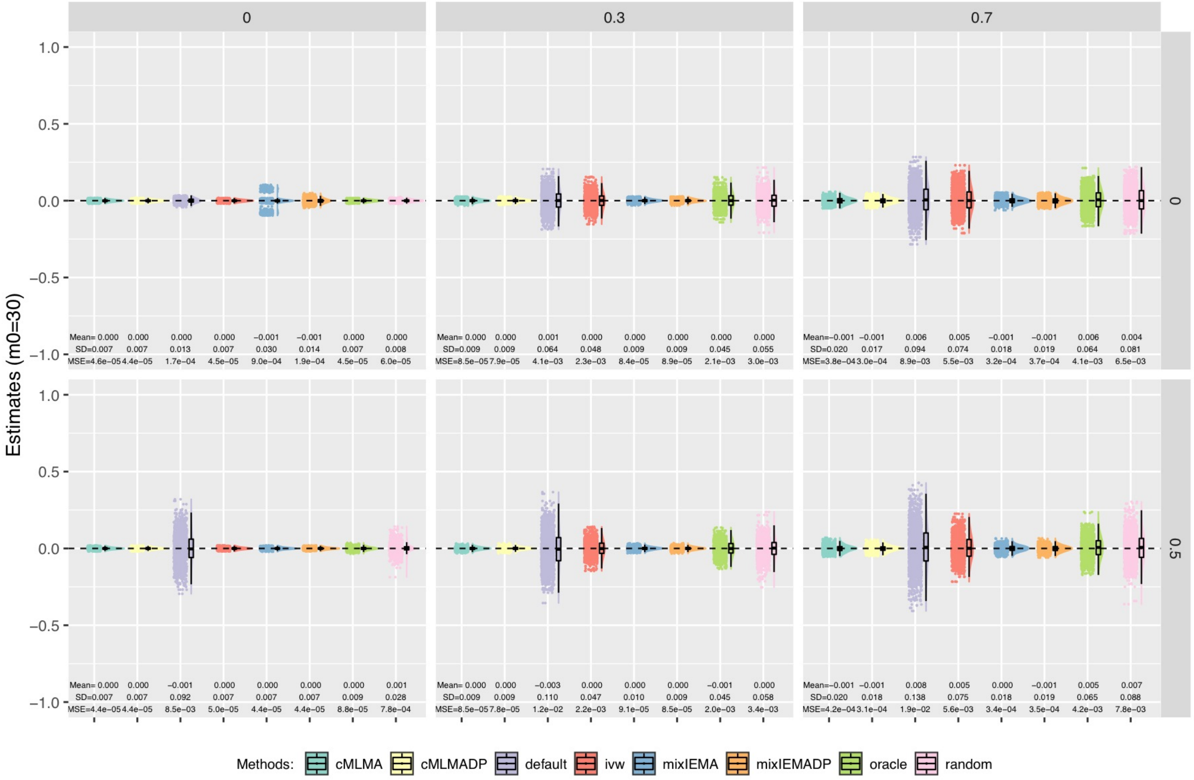

Fig L. Simulation (a) results with directional pleiotropy, $n = 50\,000, m_0 = 30, \theta = 0$. Empirical distributions of the estimates of the causal effect θ by the methods. Each column corresponds to 0%, 30% or 70% invalid IVs. Top: Irrelevant IVs do not have direct effect. Bottom: 50% of irrelevant IVs have direct effect.

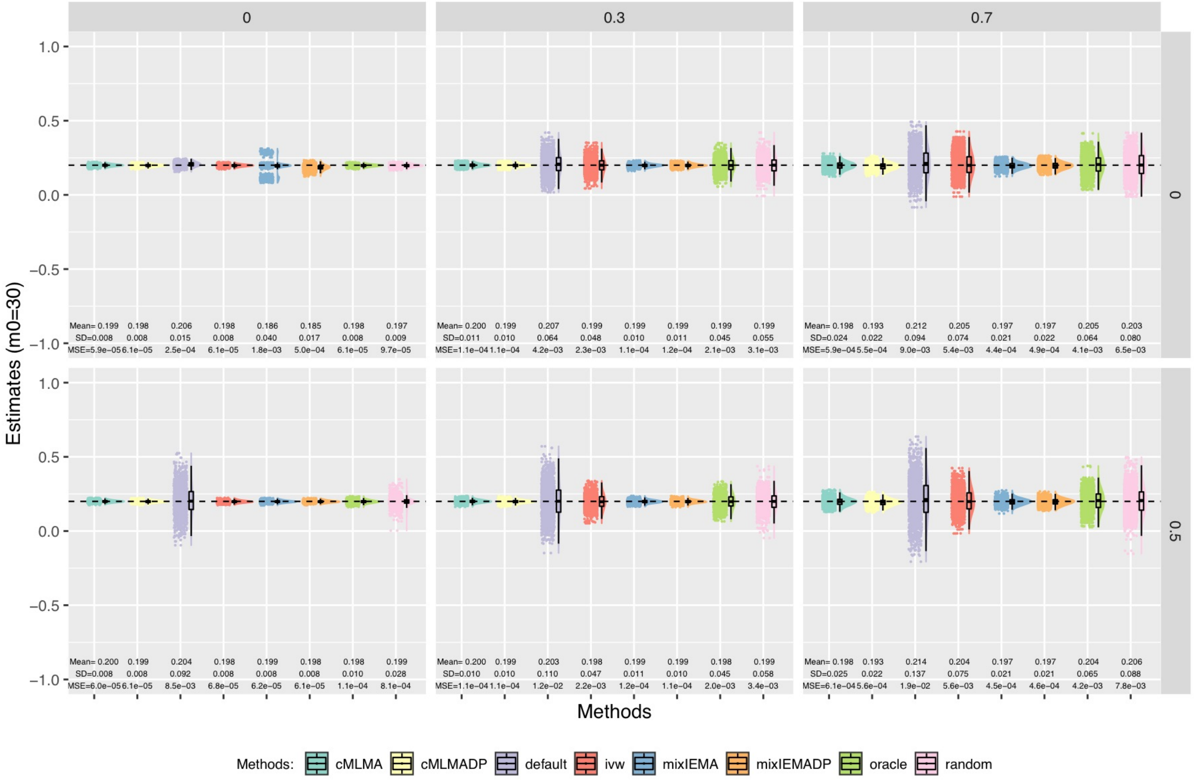

Fig M. Simulation (a) results with directional pleiotropy, $n = 50\,000, m_0 = 30, \theta = 0.2$. Empirical distributions of the estimates of the causal effect θ by the methods. Each column corresponds to 0%, 30% or 70% invalid IVs. Top: Irrelevant IVs do not have direct effect. Bottom: 50% of irrelevant IVs have direct effect.

G.2 Simulation (b): directional pleiotropy

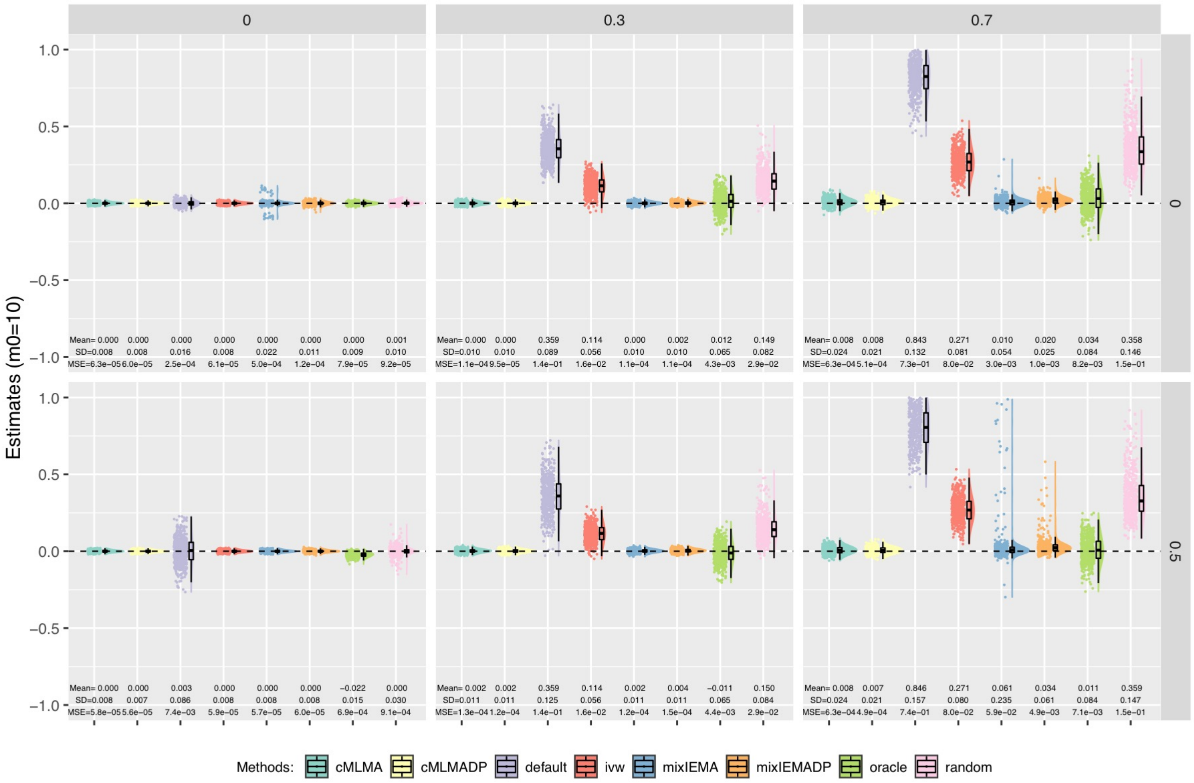

Fig N. Simulation (b) results with directional pleiotropy, $n = 50\,000, m_0 = 10, \theta = 0$. Empirical distributions of the estimates of the causal effect θ by the methods. Each column corresponds to 0%, 30% or 70% invalid IVs. Top: Irrelevant IVs do not have direct effect. Bottom: 50% of irrelevant IVs have direct effect.

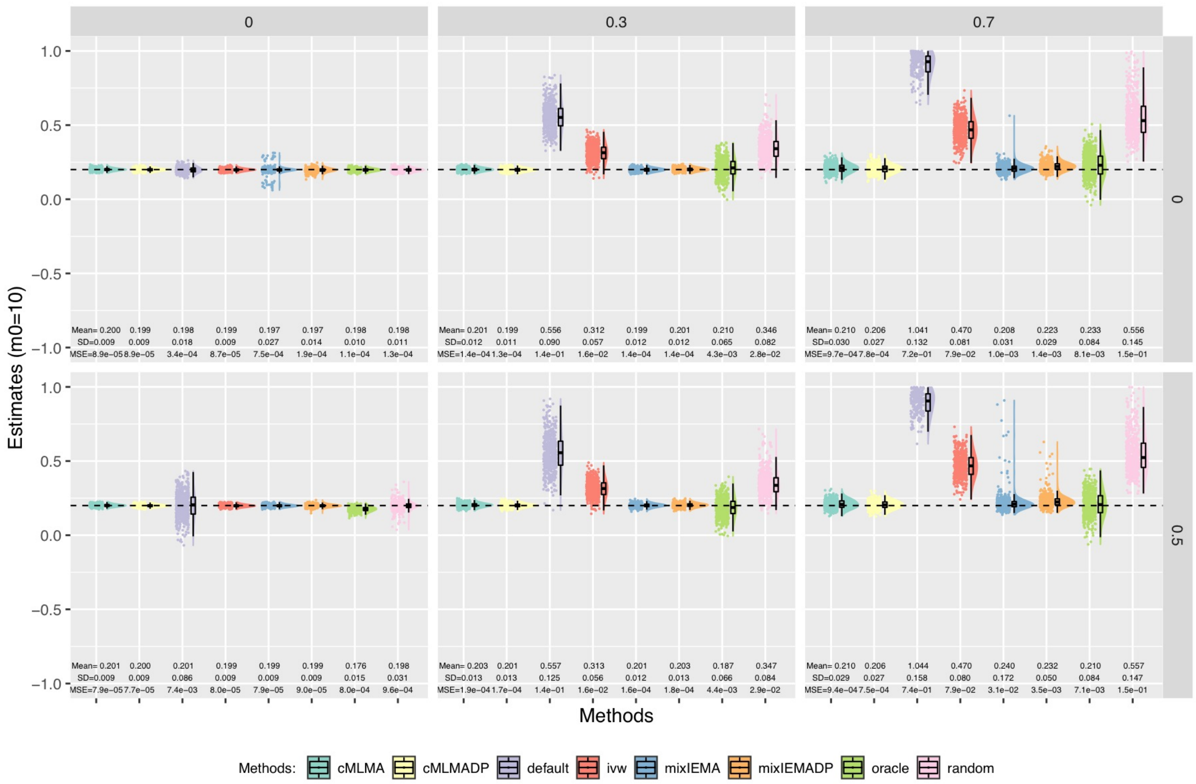

Fig O. Simulation (b) results with directional pleiotropy, $n = 50\,000, m_0 = 10, \theta = 0.2$. Empirical distributions of the estimates of the causal effect θ by the methods. Each column corresponds to 0%, 30% or 70% invalid IVs. Top: Irrelevant IVs do not have direct effect. Bottom: 50% of irrelevant IVs have direct effect.

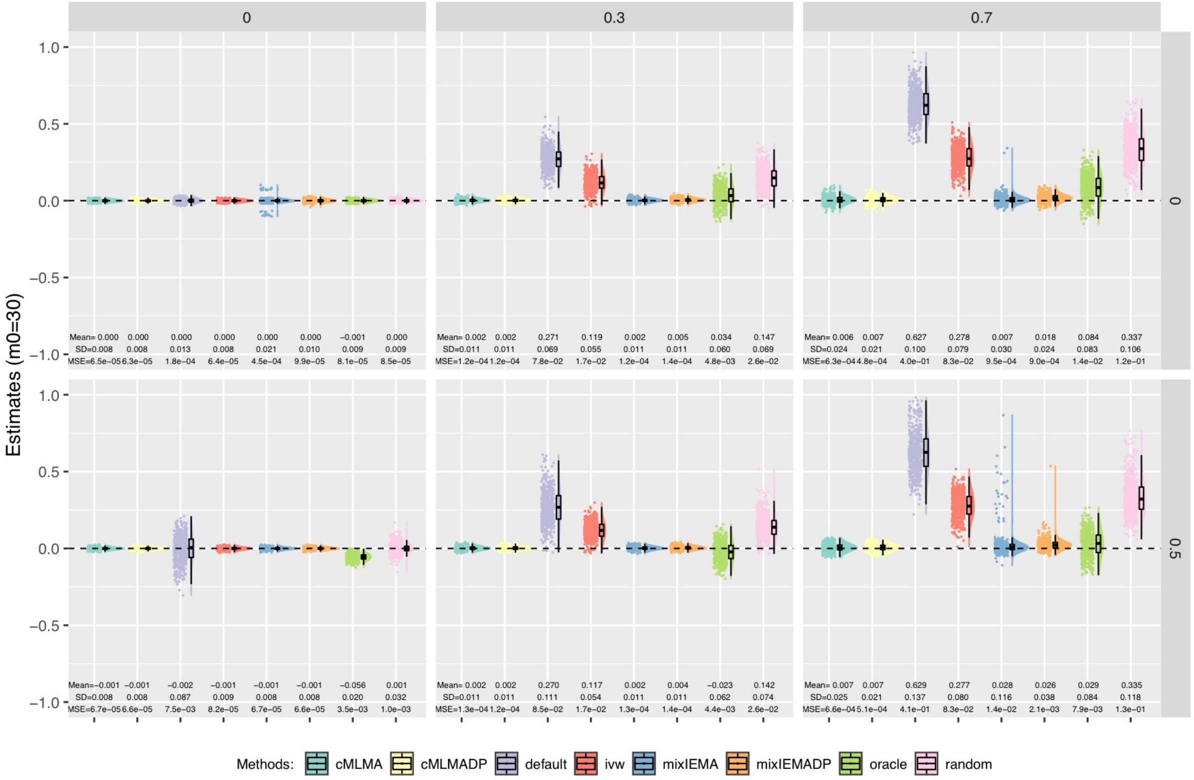

Fig P. Simulation (b) results with directional pleiotropy, $n = 50\,000, m_0 = 30, \theta = 0$. Empirical distributions of the estimates of the causal effect θ by the methods. Each column corresponds to 0%, 30% or 70% invalid IVs. Top: Irrelevant IVs do not have direct effect. Bottom: 50% of irrelevant IVs have direct effect.

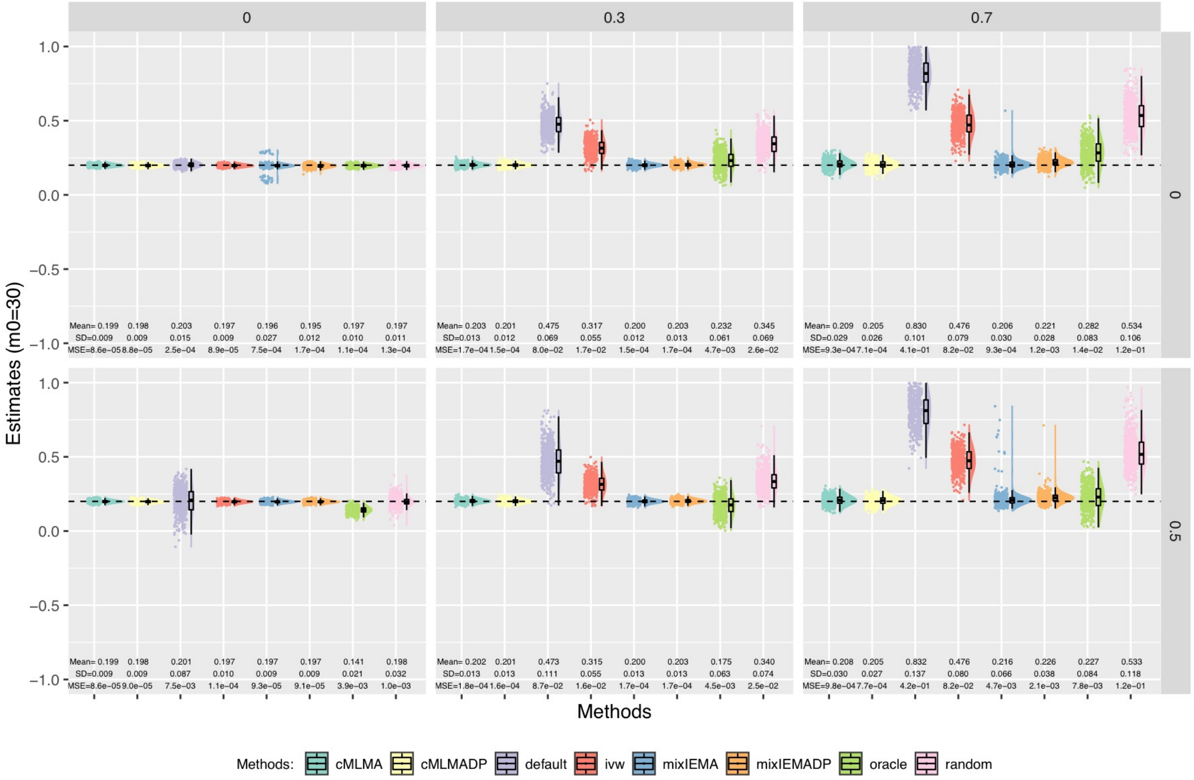

Fig Q. Simulation (b) results with directional pleiotropy, $\boldsymbol{n = 50\,000, m_0 = 30, \theta = 0.2}$. Empirical distributions of the estimates of the causal effect θ by the methods. Each column corresponds to 0%, 30% or 70% invalid IVs. Top: Irrelevant IVs do not have direct effect. Bottom: 50% of irrelevant IVs have direct effect.

H Additional simulations for intercept testing in MR-Egger

In the Egger regression model (Eq.(6) in the main text), testing whether the intercept r differs from zero can be used to detect the presence of invalid IVs with pleiotropic effect. The intercept test does not require the InSIDE assumption to hold [3]. In particular, correlated pleiotropy is a main source of IV assumption violations, which may lead to a non-zero intercept in MR-Egger. In this section, we performed more simulations to study the performance of the intercept testing in MR-Egger using various SNP coding schemes.

A general causal model is given as follows:

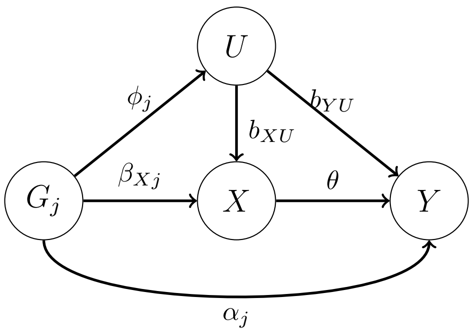

Fig R. A general causal diagram.

We have

$$b_{Xj} = \beta_{Xj} + b_{XU}\phi_j, \tag{5}$$

$$b_{Yj} = \theta b_{Xj} + b_{YU}\phi_j + \alpha_j := \theta b_{Xj} + r_j, \tag{6}$$

where b_{Xj} and b_{Yj} are the total effects of SNP G_j on the exposure X and the outcome Y respectively, and r_j is the total pleiotropic effect of G_j on Y not mediated through X. With two independent GWAS datasets on traits X and Y respectively, we have

$$\hat{\beta}_{Xj} = b_{Xj} + \epsilon_{Xj}, \quad \epsilon_{Xj} \sim \mathcal{N}(0, \sigma^2_{Xj}), \quad j = 1, \ldots, m, \tag{7}$$

$$\hat{\beta}_{Yj} = \theta b_{Xj} + r_j + \epsilon_{Yj}, \quad \epsilon_{Yj} \sim \mathcal{N}(0, \sigma^2_{Yj}), \quad j = 1, \ldots, m. \tag{8}$$

Under the NOME assumption (i.e. $\hat{\beta}_{Xj} = b_{Xj}$) and the assumption of a linear relationship between r_j and b_{Xj} (e.g. when r_j and b_{Xj} have a joint normal distribution), we have

$$E(\hat{\beta}_{Yj}|b_{Xj}) = \theta b_{Xj} + E(r_j|b_{Xj}) = \theta b_{Xj} + (\theta_1 b_{Xj} + r) = (\theta + \theta_1)b_{Xj} + r, \tag{9}$$

$$\theta_1 = \frac{\text{cov}(b_{Xj}, r_j)}{\text{var}(b_{Xj})} = \frac{b_{YU}\text{cov}(\phi_j, \beta_{Xj}) + b_{XU}b_{YU}\text{var}(\phi_j) + b_{XU}\text{cov}(\phi_j, \alpha_j) + \text{cov}(\beta_{Xj}, \alpha_j)}{\text{var}(\beta_{Xj}) + 2b_{XU}\text{cov}(\phi_j, \beta_{Xj}) + b^2_{XU}\text{var}(\phi_j)}, \tag{10}$$

where cov() and var() are the covariance and variance respectively. The MR-Egger causal estimator $\hat{\theta}_{Egger}$ is consistent for $\theta + \theta_1$ in Eq. (9), and the MR-Egger intercept estimator $\hat{r}$ is consistent for $r = E(r_j|b_{Xj} = 0)$, which can be interpreted as the average (extrapolated) pleiotropic effect for the SNPs with $b_{Xj} = 0$, or $r = E(r_j)$ as the average pleiotropic effect

if $\theta_1 = 0$ (e.g. when the InSIDE assumption holds).

H.1 Simulation set-ups

Following the causal model, we simulated data from the following model Eq. (11):

$$\begin{aligned} U &= \sum_{j=1}^{m} \phi_j G_j + \epsilon_U, \\ X &= \sum_{j=1}^{m} \beta_{Xj} G_j + U + \epsilon_X, \\ Y &= \sum_{j=1}^{m} \alpha_j G_j + \theta X + b_{YU} U + \epsilon_Y, \end{aligned} \tag{11}$$

where $m = 30$ SNPs, $G_j \sim \text{Binomial}(2, 0.3)$, and $\epsilon_U, \epsilon_X, \epsilon_Y \sim \mathcal{N}(0, 1)$ independently. As in the main text, we simulated β_{Xj} from (a) a uniform distribution on $(-0.2, -0.1) \cup (0.1, 0.2)$; (b) a uniform distribution on $(-0.1, -0.03) \cup (0.1, 0.2)$ and (c) a uniform distribution on $(0.1, 0.3)$. We considered 0% invalid IV, or 50% invalid IVs with $\alpha_j \neq 0$ and/or $\phi_j \neq 0$. When $\alpha_j \neq 0$, we generated $\alpha_j \sim \mathcal{N}(0.1, 0.1^2)$ iid and $\phi_j \sim \mathcal{U}(0, b)$ iid, with $b \in \{0, 0.1, 0.4\}$. (Note that when $b = 0$, $\phi_j = 0$.) When $\alpha_j = 0$, we generated $\phi_j \sim \mathcal{U}(0, b)$ iid, with $b \in \{0.1, 0.4\}$. We also varied $b_{YU} \in \{-1, -0.5, 1, 3\}$. The sample size for the GWAS dataset was set to be $n = 100\,000$, and the true causal effect θ was set to be 0.2.

We performed 1000 replications for each simulation scenario and compared the empirical type-I error and power using different SNPs codings in MR-Egger. As in the main text, we used (i) the default coding, i.e., the exposure-increasing allele coding; (ii) the oracle coding that was used to generated the data in Eq. (11); and (iii) the random coding. We note that, different from the oracle coding in the main text, here the (weak) InSIDE assumption might not hold under the oracle coding since we also generated correlated pleiotropy (when $\phi_j \neq 0$). But we still call it oracle coding to imply that under this coding, α_j, ϕ_j and β_{Xj} were still mutually independent.

H.2 Using the default coding controlled the type-I error of the intercept test when all IVs were valid

As shown in Fig. S, under all scenarios, when there was no invalid IV, MR-Egger with any coding scheme, including the default coding, was able to control the type-I error of the intercept test.

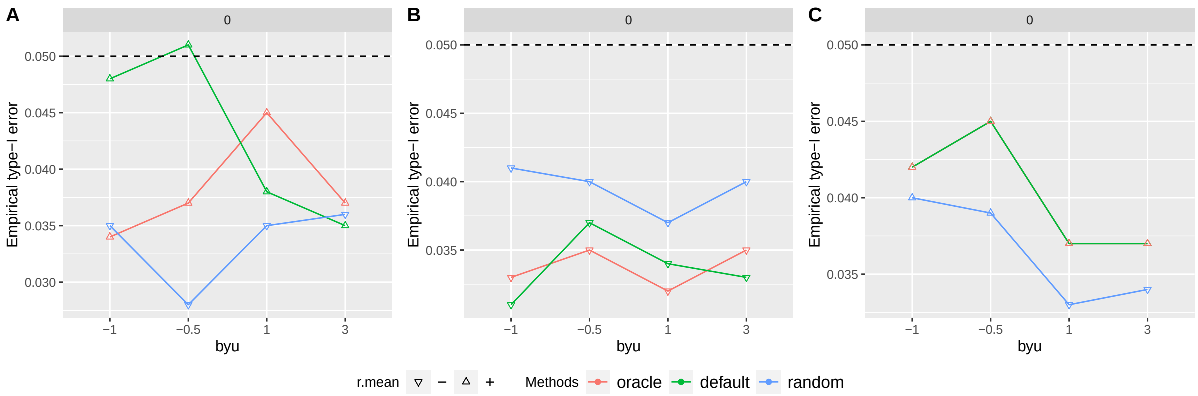

Fig S. Empirical type-I error of testing the intercept in MR-Egger in the absence of invalid IV. Panel A: Simulation (a) with $\beta_{Xj} \sim \mathcal{U}((-0.2, -0.1) \cup (0.1, 0.2))$. Panel B: Simulation (b) with $\beta_{Xj} \sim \mathcal{U}((-0.1, -0.03) \cup (0.1, 0.2))$. Panel C: Simulation (c) with $\beta_{Xj} \sim \mathcal{U}(0.1, 0.3)$.

H.3 Using the default coding was more powerful than using random codings

Figs. T and V show the empirical power of testing the intercept in MR-Egger when $\alpha_j \neq 0$ and $\alpha_j = 0$ respectively. We can see that the power of the intercept test using the default coding could be very high or very low, depending on the true data generating model. But in general, it was more powerful than the random coding. Moreover, the interpretation of the intercept in MR-Egger as an average pleiotropic effect relies on that the InSIDE assumption holds [3], as shown in the first column of Fig. U under the oracle coding. Otherwise, the presence of a non-zero intercept term was more likely to reflect some violations of the IV assumptions. We could see that in some scenarios, the intercept testing in MR-Egger using the oracle coding and the default coding both had relatively high power, but the signs of estimated intercepts from the two coding schemes

were opposite.

We also point out that, as the Editors suggested, one reasonable scenario is the following: Suppose the causal effect of X on Y is positive; the confounder has some effects on X and Y in the same direction, say both positive; when the IVs are associated with the unmeasured confounder, the SNPs under the default (i.e. exposure-increasing allele) coding will be more likely to have positive effects on Y. This scenario was reflected in Fig. V Panel C and Fig. W Panel C when $b_{YU} > 0$. However, as shown in our simulation results, even though the pleiotropic effects of the SNPs on Y were positive, the estimated intercepts were often negative.

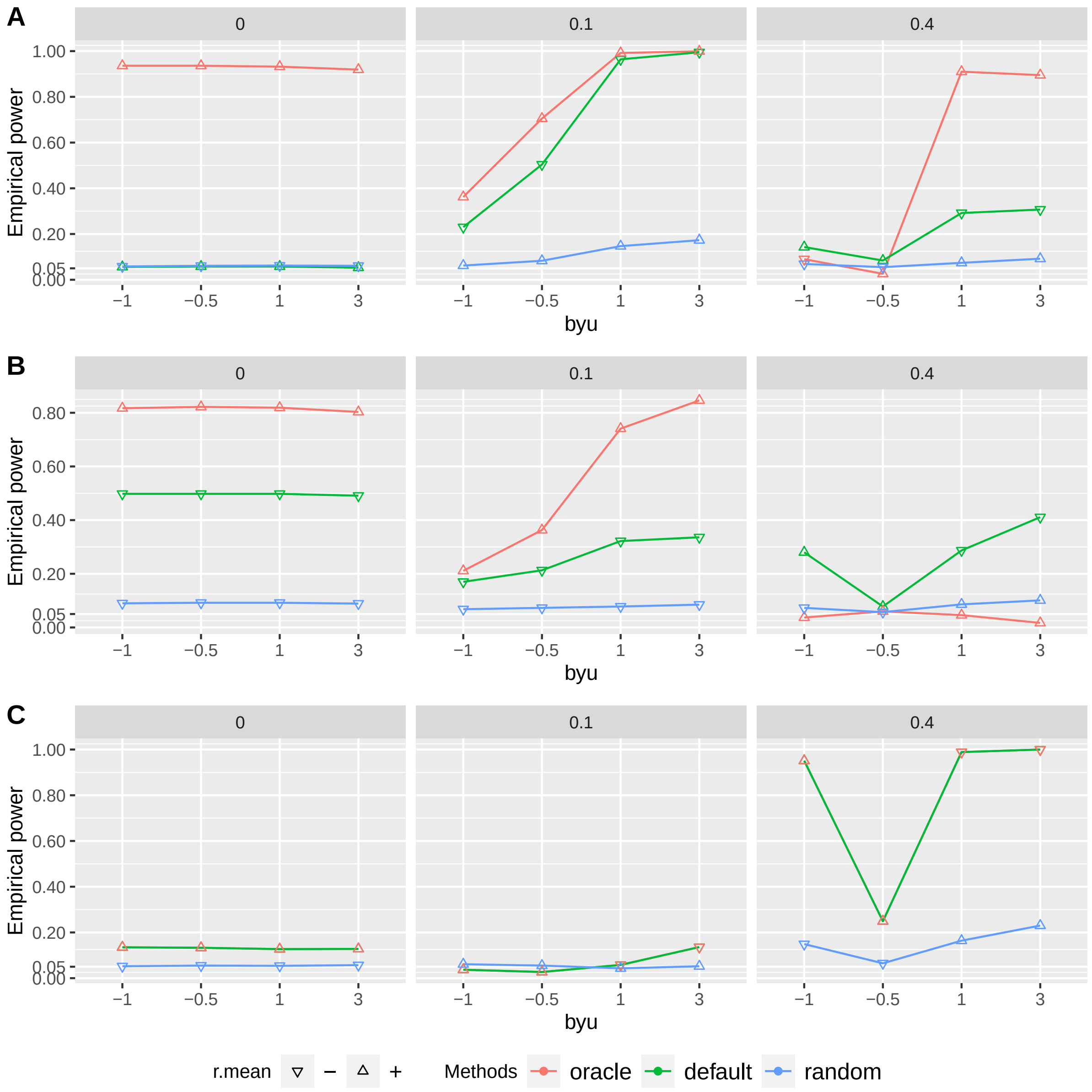

Fig T. Empirical power of testing the intercept in MR-Egger in the presence of invalid IVs with $\alpha_j \sim \mathcal{N}(0.1, 0.1^2)$ and $\phi_j \sim \mathcal{U}(0, b)$. From left to right, $b = 0, 0.1, 0.4$. Panel A: Simulation (a) with $\beta_{Xj} \sim \mathcal{U}((-0.2, -0.1) \cup (0.1, 0.2))$. Panel B: Simulation (b) with $\beta_{Xj} \sim \mathcal{U}((-0.1, -0.03) \cup (0.1, 0.2))$. Panel C: Simulation (c) with $\beta_{Xj} \sim \mathcal{U}(0.1, 0.3)$.The shape of triangle correspond to the sign of the mean of $\hat{r}$ across 1000 replications.

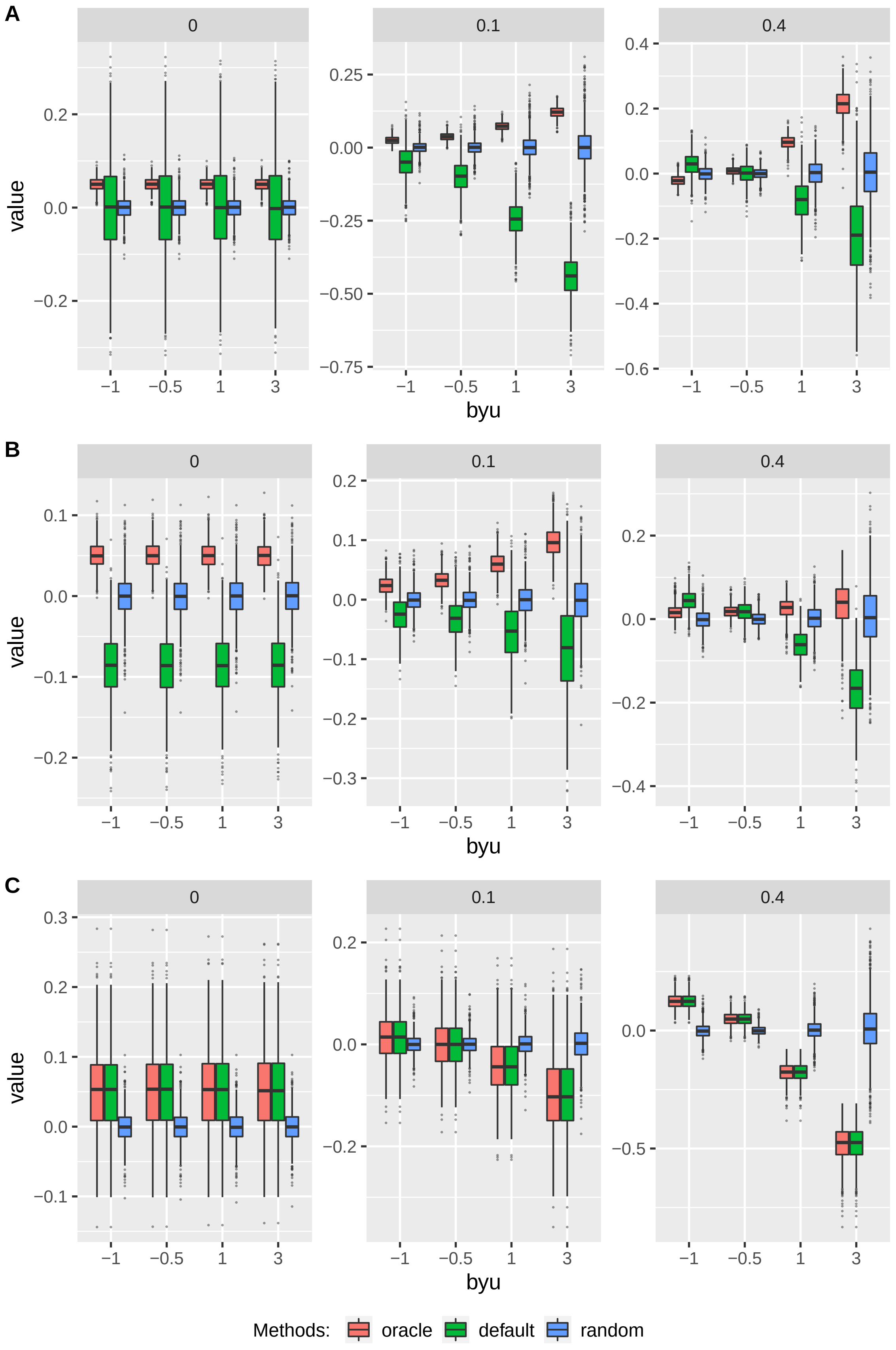

Fig U. Boxplots of estimated intercept ($\hat{r}$) across 1000 replications in the presence of invalid IVs with $\alpha_j \sim \mathcal{N}(0.1, 0.1^2)$ and $\phi_j \sim \mathcal{U}(0, b)$. From left to right, $b = 0, 0.1, 0.4$. Panel A: Simulation (a). Panel B: Simulation (b). Panel C: Simulation (c).

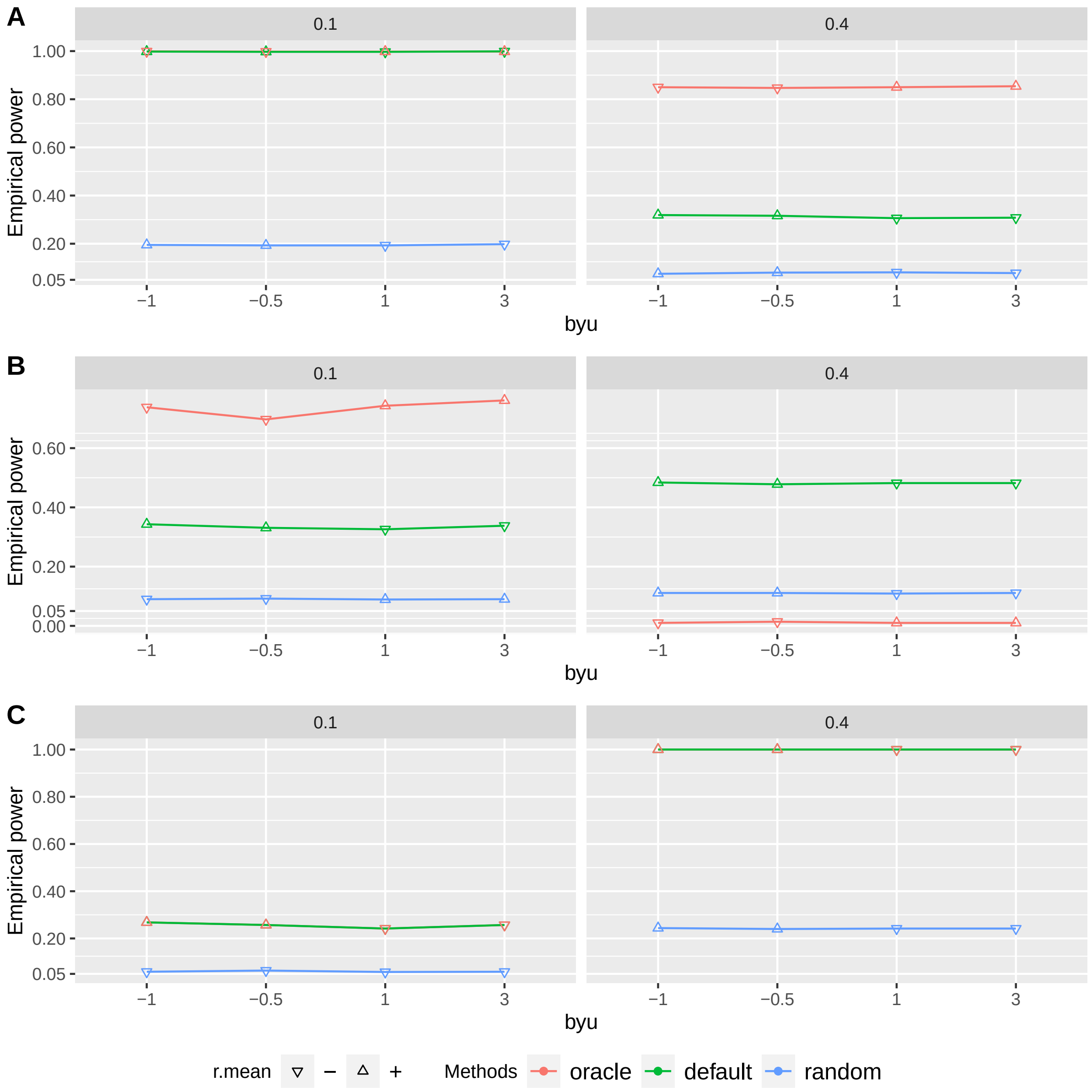

Fig V. Empirical power of testing the intercept in MR-Egger in the presence of invalid IVs with $\alpha_j = 0$ and $\phi_j \sim \mathcal{U}(0, b)$. Left: $b = 0.1$. Right: $b = 0.4$. Panel A: Simulation (a) with $\beta_{Xj} \sim \mathcal{U}((-0.2, -0.1) \cup (0.1, 0.2))$. Panel B: Simulation (b) with $\beta_{Xj} \sim \mathcal{U}((-0.1, -0.03) \cup (0.1, 0.2))$. Panel C: Simulation (c) with $\beta_{Xj} \sim \mathcal{U}(0.1, 0.3)$. The shape of triangle correspond to the sign of the mean of $\hat{r}$ across 1000 replications.

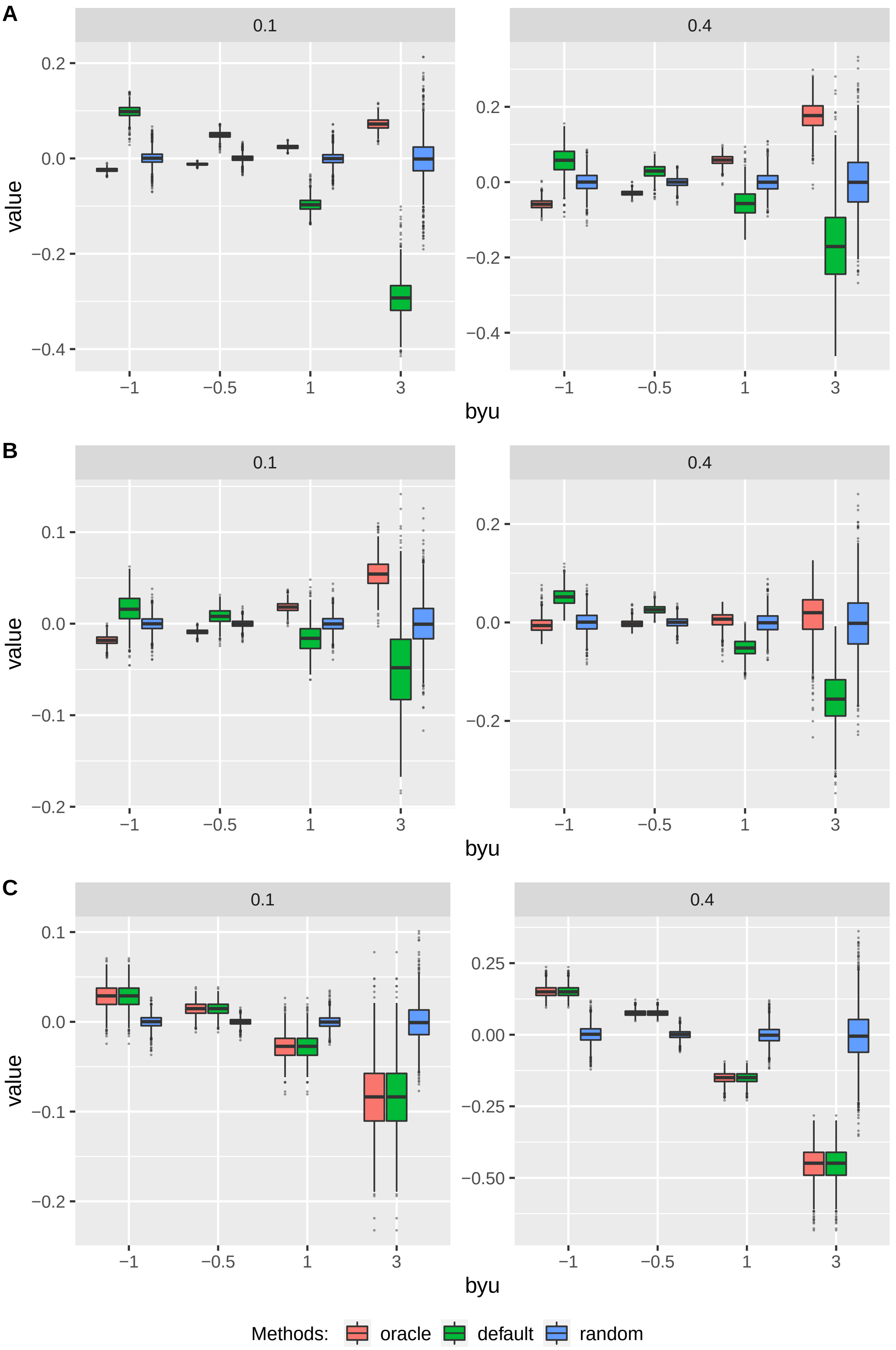

Fig W. Boxplots of estimated intercept ($\hat{r}$) across 1000 replications in the presence of invalid IVs with $\alpha_j = 0$ and $\phi_j \sim \mathcal{U}(0, b)$. From left to right, $b = 0, 0.1, 0.4$. Panel A: Simulation (a). Panel B: Simulation (b). Panel C: Simulation (c).

In summary, we could draw two main conclusions regarding the the intercept testing in MR-Egger. First, when there was no invalid IV with pleiotropy, the intercept in the MR-Egger regression model was expected to be zero, no matter what coding scheme was used; using the default coding was able to control the type-I error in the intercept test. Second, using the default coding tended to be more powerful than using a random coding scheme in detecting a non-zero intercept, implying the violation of IV assumptions (ii) and/or (iii), though it could be less or more powerful than using the 'oracle' coding in our simulation study, depending on the true data generating distribution. From this perspective, we could still stick to the default coding in practice as the intercept test could provide useful information about whether there is any invalid IV. However, it is also important to keep in mind that a non-zero intercept in MR-Egger could mean several different things. As shown in the simulation, it could be due to the presence of correlated pleiotropy, or uncorrelated (directional) pleiotropy, or both. In addition, the power of testing the intercept could be low.

References

1. Bowden J, Del Greco M F, Minelli C, Davey Smith G, Sheehan NA, Thompson JR. Assessing the suitability of summary data for two-sample Mendelian randomization analyses using MR-Egger regression: the role of the I2 statistic. International journal of epidemiology. 2016;45(6):1961–1974.

2. Yuan Z, Zhu H, Zeng P, Yang S, Sun S, Yang C, et al. Testing and controlling for horizontal pleiotropy with probabilistic Mendelian randomization in transcriptome-wide association studies. Nature communications. 2020;11(1):1–14.

3. Burgess S, Thompson SG. Interpreting findings from Mendelian randomization using the MR-Egger method. European journal of epidemiology. 2017;32(5):377–389.
